# Palladium-Based Nanocomposites Remodel Osteoporotic Microenvironment by Bone-Targeted Hydrogen Enrichment and Zincum Repletion

**DOI:** 10.34133/research.0540

**Published:** 2024-12-17

**Authors:** Lubing Liu, Huiying Liu, Xiaoya Lu, Zhengshuai Yin, Wei Zhang, Jing Ye, Yingying Xu, Zhenzhen Weng, Jun Luo, Xiaolei Wang

**Affiliations:** ^1^ The Department of Rehabilitation Medicine, the 2^nd^ Affiliated Hospital, Jiangxi Medical College, Nanchang University, Nanchang 330006, China.; ^2^ The Jiangxi Province Key Laboratory of Precision Cell Therapy, the 2^nd^ Affiliated Hospital, Jiangxi Medical College, Nanchang University, Nanchang 330006, China.; ^3^ The National Engineering Research Center for Bioengineering Drugs and the Technologies, Institute of Translational Medicine, Nanchang University, Nanchang 330088, China.

## Abstract

Osteoporosis presents a marked global public health challenge, characterized by deficient osteogenesis and a deteriorating immune microenvironment. Conventional clinical interventions primarily target osteoclast-mediated bone damage, yet lack a comprehensive therapeutic approach that balances bone formation and resorption. Herein, we introduce a bone-targeted nanocomposite, A-Z@Pd(H), designed to address these challenges by integrating diverse functional components. The nanocomposite incorporates internal hydrogen-carrying nanozymes, which effectively scavenge multiple reactive oxygen species (ROS) and synergistically engage the autophagy–lysosome pathway to accelerate endogenous ROS degradation in macrophages. This mechanism disrupts the vicious cycle of autophagic dysfunction–ROS accumulation–macrophage inflammation. In addition, external metal–organic frameworks release zinc ions (Zn^2+^) in response to the acidic osteoporotic environment, thereby promoting osteogenesis. In a murine model of osteoporosis, intravenous administration of A-Z@Pd(H) leads to preferential accumulation in the femur, thereby remodeling the osteoporotic microenvironment through immune regulation, osteogenesis promotion, and osteoclast inhibition. These findings suggest that this system composed of hydrogen therapy and ion therapy may be a promising candidate for bone-targeted comprehensive therapy in osteoporosis.

## Introduction

Osteoporosis (OP), characterized by chronic bone demineralization and immune microenvironment deterioration, significantly impacts patients’ quality of life and imposes a substantial financial burden. With over 200 million individuals affected globally, especially postmenopausal women and the elderly, its prevalence is projected to rise in the coming years [1,2]. The persistent imbalance between bone formation and resorption underlies OP, and osteoimmunomodulation for the bone balance by mediating reciprocal interactions between immune and bone cells has gained increased attention [3,4]. However, current clinical interventions primarily target to inhibit osteoclast (OC) maturation to mitigate bone resorption, often using antiresorptive medications like bisphosphonates and denosumab [5,6]. In addition, monoclonal antibody biotherapy poses risks of immune microenvironment disruption [7,8]. Overall, existing treatments lack a comprehensive strategy to maintain bone homeostasis, especially in immune regulation throughout the progression of OP.

As pivotal members of the bone-resident immune cells, macrophages play dual roles: They induce progenitor cells toward an osteoblast fate and serve as precursors to OCs in bone resorption processes [9,10]. Macrophage polarization critically regulates the balance between the 2 processes of bone marrow stromal cells–osteoblasts and monocytes–macrophages–OCs [11,12], often linked to intracellular autophagic activity, an evolutionarily well-conserved recycling process responding to stress conditions [13,14]. Reactive oxygen species (ROS) are involved in autophagy regulation, thereby impacting macrophage function [15,16]. ROS have been demonstrated to accelerate M1-phenotype (pro-inflammatory phase) polarization and OC differentiation of bone macrophages, and autophagy may participate in the M2-phenotype (anti-inflammatory phase) polarization of macrophages [17–20]. Collectively, autophagy dysfunction, ROS accumulation, and macrophage inflammation form a reinforcing cycle, leading to abnormal OC activity and impaired osteogenesis. Thus, maintaining bone homeostasis in OP requires a systemic therapy that encompasses microscopic ROS clearance and restoration of autophagic flux to modulate macrophage function, as well as macroscopic reconstruction of the bone tissue.

In recent years, ROS-targeted antioxidant therapy has garnered considerable attention in the OP research community [21–24]. Compared to conventional antioxidants, hydrogen molecules (H_2_) have emerged as a safe, effective, and broad-spectrum anti-inflammatory agent owing to their anti-oxidation ability for selectively scavenging highly oxidative/toxic radicals such as hydroxyl radicals (•OH) [25–27]. While data specifically addressing hydrogen therapy in bone studies are limited, emerging evidence suggests its efficacy in immune modulation and OC suppression [27,28]. The previously reported H_2_ administration routes mainly include oral uptake of hydrogen-rich water (HRW), inhalation of mixed gases, and intravenous administration of hydrogen-rich saline. However, these methods face challenges in maintaining high H_2_ concentrations at the target site over extended periods due to the low solubility and high dispersibility of H_2_. Moreover, these methods lack precision in delivering hydrogen to the systemic bone microenvironment. Recent studies involving osteoporotic zebrafish treated with HRW have highlighted its ability to selectively impact OC activity without considerable effects on osteoblasts [29]. Although promising, replicating this targeted approach in mammalian models poses practical challenges. To date, achieving precise targeting of hydrogen therapy to bone tissue remains an unattainable goal, presenting major challenges to its advancement for OP treatment. In addition, antioxidant studies in OP treatment often overlook the concomitant supplementation with osteogenesis promoters.

**Fig. 1.**
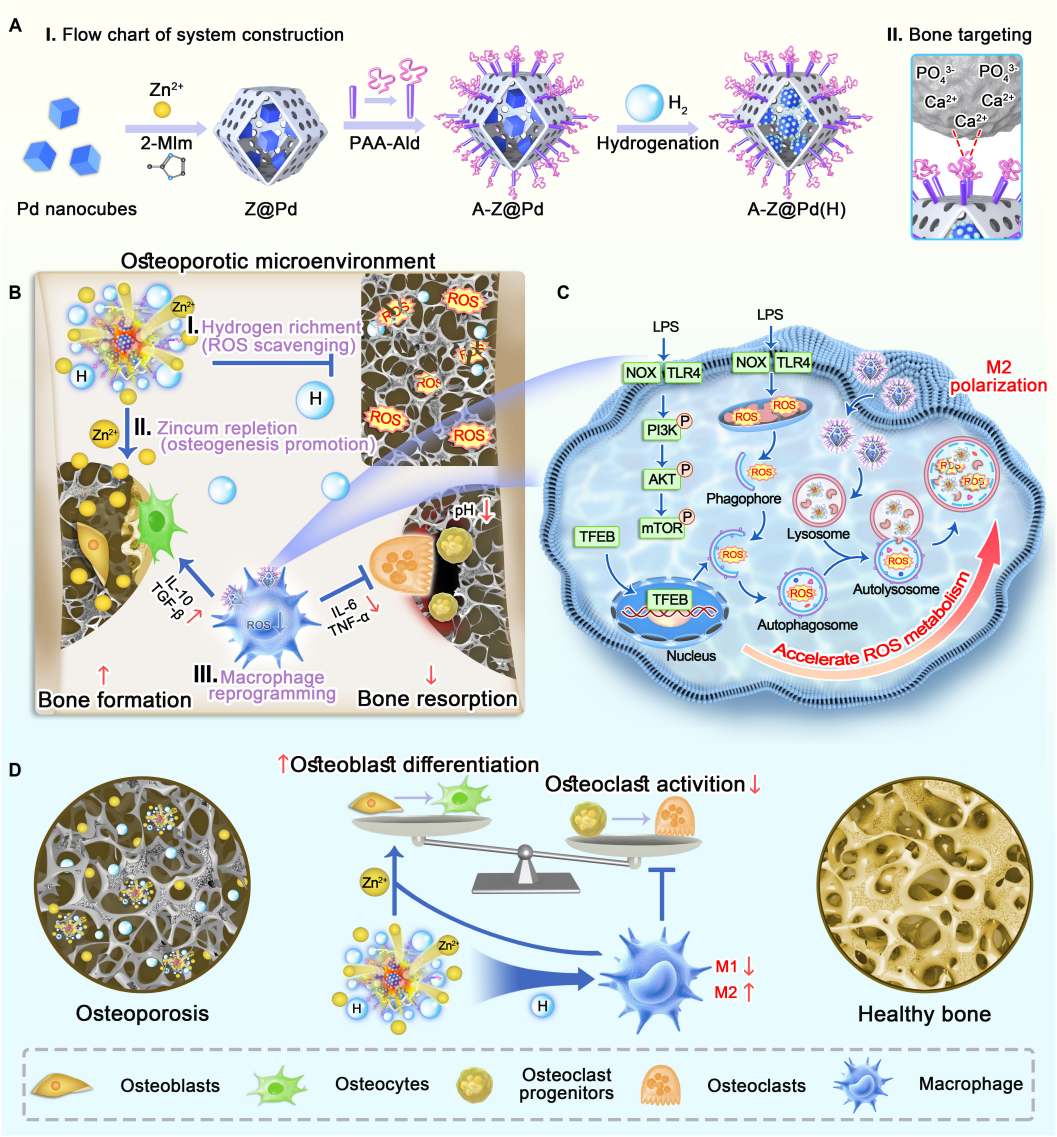
Schematic illustration of a bone-targeted nanocomposite for hydrogen enrichment and zincum repletion in OP. (A) Schematic diagram of A-Z@Pd(H). (A-I) Synthesis process. (A-II) Bone-targeting mechanism. (B) Treatment mechanism of A-Z@Pd(H) in the osteoporotic microenvironment. (B-I) Efficient removal of multiple ROS by A-Z@Pd(H). (B-II) Release of Zn^2+^ in response to the acidic osteoporotic environment, enhancing osteogenic behavior. (B-III) Transformation of macrophages from M1 to M2 phenotype by A-Z@Pd(H), facilitating osteoblast promotion and OC inhibition via immunomodulatory effects. (C) Synergistic effect of A-Z@Pd(H) with the autophagy–lysosome pathway to accelerate intracellular ROS metabolism. (D) Bone homeostasis benefits from osteogenesis promotion by Zn^2+^ and macrophage regulation by reductive hydrogen.

In this work, we have developed a nanocomposite named A-Z@Pd(H) for systemic hydrogen therapy and zinc repletion targeting bone tissue (Fig. 1A). Palladium (Pd) nanozymes have received growing interest due to their high biocompatibility and good antioxidative activity [30,31]. In addition, H_2_ can be embedded into the lattice of Pd nanoparticles [32], forming reductive hydrogen, which can selectively enhance the elimination of superoxide anions (O_2_^•−^) and hydroxyl radicals (•OH) [33,34]. The encapsulation of zeolitic imidazolate framework-8 (ZIF-8) could improve hydrogen storage capacity [35], and further modification with alendronate (Ald) could enable the accumulation of the nanocomposite in bone tissue (Fig. 1A-II). Reductive hydrogen released from A-Z@Pd(H) exhibited potent scavenging of multiple ROS within the bone microenvironment (Fig. 1B-I), while zinc ions (Zn^2+^) released in response to the acidic osteoporotic environment enhanced osteogenic behavior (Fig. 1B-II) [36–38], thereby compensating for the absence of direct hydrogen effects on osteogenesis [29]. Furthermore, A-Z@Pd(H) was found to ameliorate autophagy–lysosome system impairment in macrophages under inflammatory conditions. This effect might be attributed to the suppression of the phosphatidylinositol 3-kinase/protein kinase b/mammalian target of rapamycin (PI3K/AKT/mTOR) signaling pathway, resulting in the transcriptional activation of the downstream target TFEB and facilitating the degradation of endogenous pro-inflammatory mediators such as ROS, consequently facilitating the transformation of M1 macrophages to M2 macrophages (Fig. 1C). Reprogrammed macrophages played active immunomodulatory effects on promoting osteoblast function and inhibiting OC activity (Fig. 1B-III). In conclusion, A-Z@Pd(H) constructed in this work integrated multiple functional components to precisely target bone tissue, thereby remodeling the osteoporotic microenvironment through immune regulation, osteogenesis promotion, and OC inhibition (Fig. 1D). This represented the first achievement of targeted hydrogen delivery throughout the bone microenvironment, with concurrent investigation of potential molecular mechanisms involved.

## Results and Discussion

### Synthesis and characterization of A-Z@Pd(H)

**Fig. 2.**
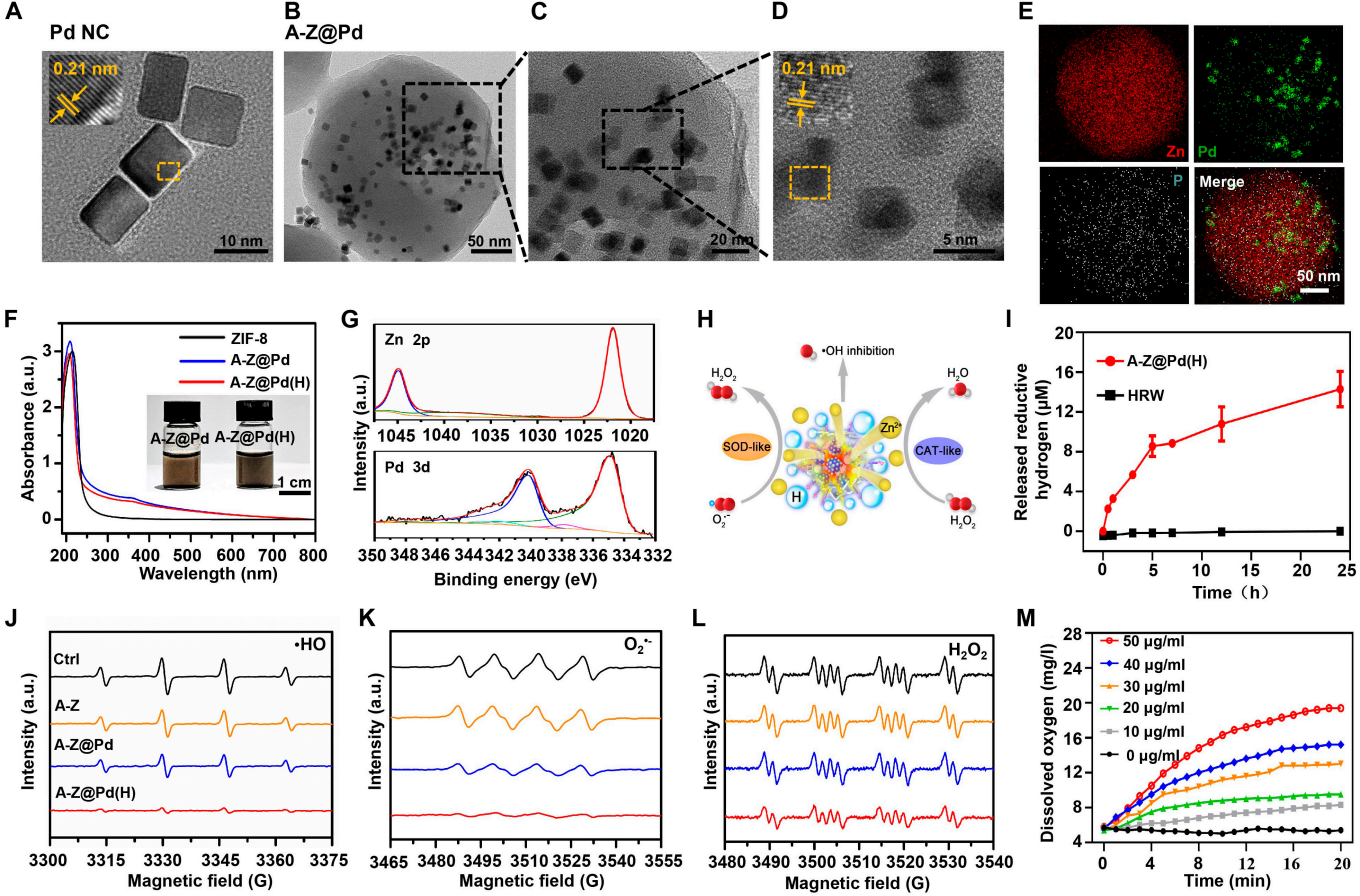
Construction and characterization of A-Z@Pd(H), as well as its reductive hydrogen-releasing and ROS-scavenging capacities. (A) HRTEM image and lattice spacing of Pd NCs. (B) TEM and (C) HRTEM images of A-Z@Pd. (D) Pd NCs inside A-Z@Pd and lattice spacing of Pd NCs. (E) Elemental mapping images of A-Z@Pd including Zn, Pd, and P. (F) UV–vis absorption spectra of ZIF-8, A-Z@Pd, and A-Z@Pd(H). Inset: Optical pictures of A-Z@Pd and A-Z@Pd(H) solutions. (G) High-resolution XPS spectra of Zn 2p and Pd 3d in A-Z@Pd(H). (H) ROS-eliminating mechanism of A-Z@Pd(H). (I) Hydrogen release behavior of A-Z@Pd(H) and HRW measured using an MB probe. (J to L) ESR spectra of A-Z, A-Z@Pd, and A-Z@Pd(H) to analyze their effect on ROS clearance including (J) •OH, (K) O_2_^•−^, and (L) H_2_O_2_. (M) Levels of dissolved oxygen catalyzed by varying concentrations of A-Z@Pd(H). Data are means ± SD (*n* ≥ 3).

Pd nanocubes (NCs) were synthesized following a previously established protocol [39]. Transmission electron microscopy (TEM) and high-resolution TEM (HRTEM) images showed that the Pd NCs had a well-defined structure with sizes of about 10 nm and lattice sizes of about 0.21 nm (Fig. 2A and Fig. S1A). Upon hydrogen loading (PdH NCs), the solution color shifted from brownish yellow to black, resulting in diminished absorption in the ultraviolet (UV) zone and enhanced absorption in the visible–near infrared (vis–NIR) zone (Fig. S1B), which aligned with earlier findings [39]. Pd NCs were added during the synthesis process of ZIF-8 to obtain the core-shell composite (Z@Pd) [35], characterized by scanning electron microscopy (SEM) to have a dodecahedral morphology similar to ZIF-8, with an approximate particle size of 150 nm (Fig. S2A to C). Subsequently, using a previously described method [40,41], the bone-targeted drug (Ald), modified covalently by poly(acrylic acid) (PAA), was electrostatically adsorbed onto the surface of Z@Pd, forming A-Z@Pd. This modification conferred bone-targeting properties to the gas nanoplatform. Fourier transform infrared (FTIR) spectroscopy revealed the successful bonding of PAA and Ald, as evidenced by the peaks representing amide groups at 1,553 cm^−1^ and phosphorus-oxygen bonds (O─P─O) at 1,076 and 568 cm^−1^ in PAA-Ald (Fig. S3A). Moreover, the presence of the O─P─O peak in A-Z@Pd verified that PAA-Ald had been successfully modified on Z@Pd (Fig. S3B). TEM (Fig. 2B) and HRTEM (Fig. 2C) images confirmed the core-shell nanostructure of the composite A-Z@Pd, with the well-encapsulated Pd NCs maintaining their original shape, size, and lattice size (Fig. 2D). TEM elemental mapping images displayed the Zn element from ZIF-8, the Pd element from Pd NCs, and the phosphorus (P) element in bone-targeting groups (Fig. 2E). X-ray diffraction (XRD) patterns (Fig. S4) showed that the pattern of A-Z@Pd was consistent with those of ZIF-8 and Pd. Zeta potential analyses revealed that PAA-Ald modification altered Z@Pd’s zeta potential from positive to approximately −20 mV (Fig. S5). These results confirmed the successful synthesis of A-Z@Pd. The nitrogen (N_2_) adsorption–desorption curve (Fig. S6A) and pore size distribution (Fig. S6B) were determined by the Brunauer–Emmett–Teller method. The results demonstrated that after the PAA-Ald modification, A-Z@Pd exhibited smaller micropores compared with Z@Pd and ZIF-8, with a minimum micropore size of about 0.51 nm, allowing the passage of hydrogen molecules. Subsequently, A-Z@Pd(H) was synthesized by introducing hydrogen into the A-Z@Pd nanoparticle solution using a hydrogen generation device. The changes in the UV–visible (UV–vis) spectra (Fig. 2F) were similar to previous observations during the hydrogen loading of Pd NCs (Fig. S1B), accompanied by a color change from brownish yellow to black, implying that the encapsulation of ZIF-8 and the modification of PAA-Ald did not interfere with hydrogen storage in Pd NCs. Additionally, x-ray photoelectron spectroscopy (XPS) analyses (Fig. 2G) certified that the main Pd species in A-Z@Pd(H) were Pd^0^ (Pd^0^3d_3/2_: 341.1 eV and Pd^0^3d_5/2_: 335.8 eV), indicating that hydrogen molecules might exist in the lattice gap of Pd NCs without forming a stable chemical bond. For the high-resolution Zn 2p XPS segment, the binding energies at 1,044.85 and 1,021.91 eV originated from Zn 2p_1/2_ and Zn 2p_3/2_, showing that the bivalent Zn was the majority in the A-Z@Pd(H), which has been demonstrated to have a promoting effect on osteogenesis [36–38].

### ROS-scavenging capacity of A-Z@Pd(H)

The encapsulation of Pd NCs inside porous materials to enhance catalytic efficiency by H_2_ enrichment has been previously reported [35]. The hydrogen released from Pd NCs has enhanced reducibility compared with free hydrogen gas, as it exists in the form of active hydrogen atoms [39], which might accelerate the removal of ROS including •OH, O_2_^•−^, and hydrogen peroxide (H_2_O_2_) (Fig. 2H) [33,34,39]. First, the effective release of reductive hydrogen was detected. Reductive hydrogen could reduce methylene blue (MB) to colorless leuco-methylene blue (LMB), leading to a decrease in UV–vis absorption peaks [33,34]. This reduction was measured by the change in absorbance of the characteristic absorption peak at 664 nm and quantified using Beer–Lambert’s law with a standard curve (Fig. S7). Figure S8 shows that the UV–vis absorption peak of MB significantly decreased in the A-Z@Pd(H) group, and the releasing of hydrogen reached the peak after 24 h (Fig. 2I), while there was almost no change in the HRW group. After that, a series of experiments were carried out to assess the ROS-scavenging performance of A-Z@Pd(H). The typical 2,2-diphenyl-1-picrylhydrazyl (DPPH) (Fig. S9A) and 2,2′-azino-bis(3-ethylbenzothiazoline-6-sulfonic acid) diammonium salt (ABTS) (Fig. S9B) assays were used to test the total antioxidant capacity. The results showed that A-Z@Pd(H) exhibited potent antioxidant activity in a concentration-dependent manner, with the composite scavenging nearly 35% of DPPH and 30% of ABTS at a low concentration of 12.5 μg/ml.

To evaluate the catalytic selectivity of A-Z@Pd(H), 3 representative ROS, including •OH, O_2_^•−^, and H_2_O_2_, were selected for detection. Initially, the Fenton reaction between ferrous ion (Fe^2+^) and H_2_O_2_ was utilized to generate •OH radicals and evaluate the scavenging efficacy of A-Z@Pd and A-Z@Pd(H). It was observed that the electron spin resonance (ESR) signal intensity significantly decreased following the A-Z@Pd(H) treatment, while no noticeable change was observed in the A-Z@Pd group (Fig. 2J). This finding demonstrated the efficient elimination of •OH radicals by A-Z@Pd(H), potentially attributed to the reductive hydrogen. ESR spectroscopy analyses of O_2_^•−^ signals bore out that both A-Z@Pd(H) and A-Z@Pd exhibited superoxide dismutase (SOD)-like activity referring to the clearance of O_2_^•−^ to generate H_2_O_2_ and O_2_, and A-Z@Pd(H) appeared to have a stronger clearance ability for O_2_^•−^ than A-Z@Pd (Fig. 2K). For the detection of H_2_O_2_ signals, the A-Z@Pd(H) and A-Z@Pd groups showed nearly identical intensities (Fig. 2L). Subsequently, the specific clearance rates of A-Z@Pd(H) and A-Z@Pd were experimentally compared. Both A-Z@Pd(H) and A-Z@Pd eliminated O_2_^•−^ radicals in a concentration-dependent manner, with approximately 50% of O_2_^•−^ radicals being removed by 200 μg/ml A-Z@Pd(H), 38% higher than that by A-Z@Pd (Fig. S10). It was also found that A-Z@Pd(H) and A-Z@Pd featured a comparable catalase (CAT)-like property referring to the decomposition of H_2_O_2_ to generate O_2_ in a dose-dependent pattern (Fig. S11A). Nearly 52% of H_2_O_2_ was scavenged when the A-Z@Pd(H) concentration was 100 μg/ml, and this inhibition rate did not change significantly over 3 cycles, indicating that A-Z@Pd(H) possessed prolonged H_2_O_2_ scavenging capability (Fig. S11B). The production of oxygen (O_2_) was observed during the catalytic process of H_2_O_2_ and increased with increasing concentrations of A-Z@Pd(H) and H_2_O_2_ (Fig. 2M and Fig. S12). Taken together, A-Z@Pd(H) could simulate the SOD and CAT cascade process, facilitating the conversion of O_2_^•−^ to H_2_O_2_ and further decomposing H_2_O_2_ into O_2_ and H_2_O [42,43]. Of note, the weaker ROS-scavenging capability of ZIF-8@Pd and the near absence of ROS-scavenging ability in ZIF-8 indicated that the observed catalytic effect of A-Z@Pd(H) was achieved through reductive hydrogen.

### In vitro cytotoxicity assessment

The biocompatibility is crucial for in vivo application of biomaterials. Through cell live/dead staining (Fig. S13) and cell counting kit-8 (CCK-8) assays (Fig. S14A to C), it was determined that A-Z@Pd(H) at concentrations up to 20 μg/ml exhibited good biocompatibility with human umbilical vein endothelial cells (HUVECs), mouse embryo osteoblast precursor cells (MC3T3-E1 cells), and mouse leukemia cells of monocyte macrophage (RAW264.7 cells), with cell viability exceeding 90% for 3 d. The majority of cells remained viable (green), with only a few apoptotic cells observed (red). Furthermore, hemolytic experiments demonstrated that A-Z@Pd(H) exhibited excellent compatibility with red blood cells (RBCs), as no significant hemolysis was observed even at concentrations up to 1,000 μg/ml, with hemolysis rates less than 5% (Fig. S14D). For subsequent cell experiments, the nanoparticle concentration was set at 20 μg/ml.

### Macrophage internalization and ROS metabolism via A-Z@Pd(H)

**Fig. 3.**
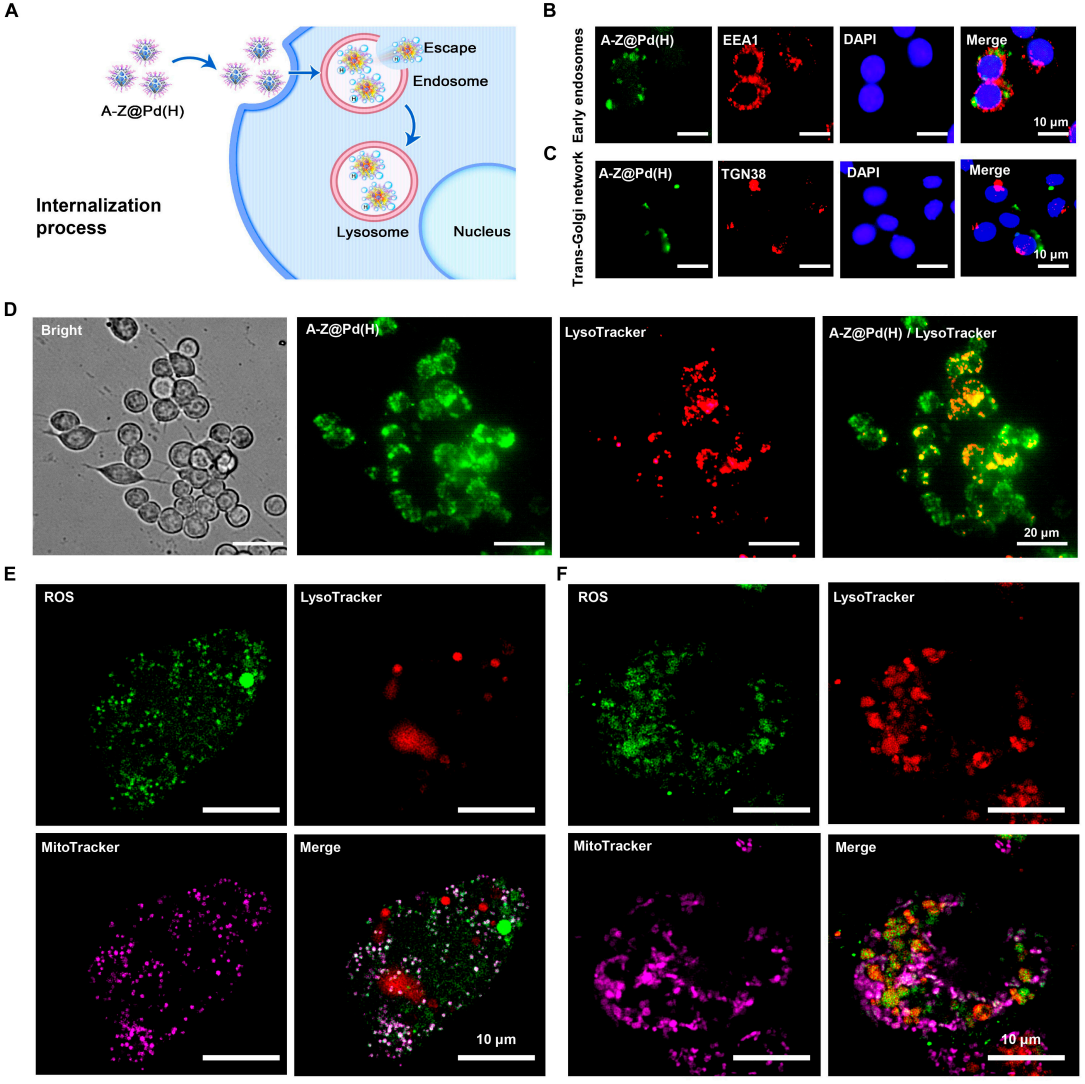
Transport of A-Z@Pd(H) within macrophages and their regulation of cells. (A) Schematic diagram of the internalization of A-Z@Pd(H) in RAW264.7 cells. (B to D) Observation by live cell imaging system showing intracellular trafficking of FITC-labeled A-Z@Pd(H) after incubation with RAW264.7 cells and colocalization with (B) early endosomal markers (EEA1), (C) trans-Golgi network markers (TGN38), and (D) lysosomal markers (LysoTracker). (E and F) Simultaneous localization images of ROS (green), lysosomes (red), and mitochondria (purple) under confocal microscopy in the (E) LPS group and (F) LPS + A-Z@Pd(H) group.

To investigate the effect of A-Z@Pd(H) on ROS metabolism in macrophages, the endocytic pathway of A-Z@Pd(H) and the localization of ROS in macrophages were explored. Fluorescein isothiocyanate (FITC)-labeled A-Z@Pd(H) was used to observe its intracellular uptake in RAW264.7 cells. The results of colocalization with the markers for early endosomes (EEA1), late endosomes (CD63), and lysosomes (LysoTracker) at different time points indicated that A-Z@Pd(H) was primarily taken up and metabolized by RAW264.7 cells through endosomal trafficking (Fig. 3A and B and Fig. S15A), while minimal colocalization with trans-Golgi network (TGN38) was observed (Fig. 3C). Endocytosed A-Z@Pd(H) displayed a time-dependent increase within 12 h, while most of the green fluorescence colocalized with the red fluorescence of LysoTracker (Fig. 3D and Fig. S15B), but some separated green fluorescence was also observed, indicating some endolysomal escape during the internalization of A-Z@Pd(H) (Fig. 3A).

Since lysosomes are essential cellular organelles for breaking down various exogenous and endogenous macromolecules [44], it is imperative to explore the effects of A-Z@Pd(H) on the production and localization of intracellular ROS. 2,7-Dichlorodihydrofluorescein diacetate (DCFH-DA) was used to explore the localization and degradation of ROS in RAW264.7 cells. Figure S16 shows that A-Z@Pd(H) itself does not induce intracellular ROS production and significantly reduces the level of ROS at 24 h induced by lipopolysaccharide (LPS) stimulation. To further analyze the dynamic fate of ROS, we simultaneously used lysosomal (LysoTracker) and mitochondrial (MitoTracker) probes to treat cells for observing and comparing the localization of ROS. The confocal microscopy observations revealed that cells stimulated solely with LPS exhibited pronounced mitochondrial shrinkage, reduced lysosome abundance, and heightened intracellular ROS levels primarily localized to mitochondria (Fig. 3E). In contrast, cells treated with A-Z@Pd(H) maintained rod-shaped mitochondrial morphology, exhibited enhanced lysosomal signal intensity, and showed significant colocalization of ROS signals with lysosomes (Fig. 3F). These findings implied that A-Z@Pd(H) potentially promoted the translocation of ROS from mitochondria to lysosomes for degradation. Although the modes and biological functions of mitochondria–lysosome communication are still largely unknown, their interaction to regulate cell metabolism is widely acknowledged [44]. In conclusion, the above results implied that there was a certain overlap between the endocytic pathway and the ROS metabolism process in the cells, which triggered the eventual low expression of ROS in an inflammatory environment.

### Effects of A-Z@Pd(H) on autophagy–lysosomal function in macrophages

**Fig. 4.**
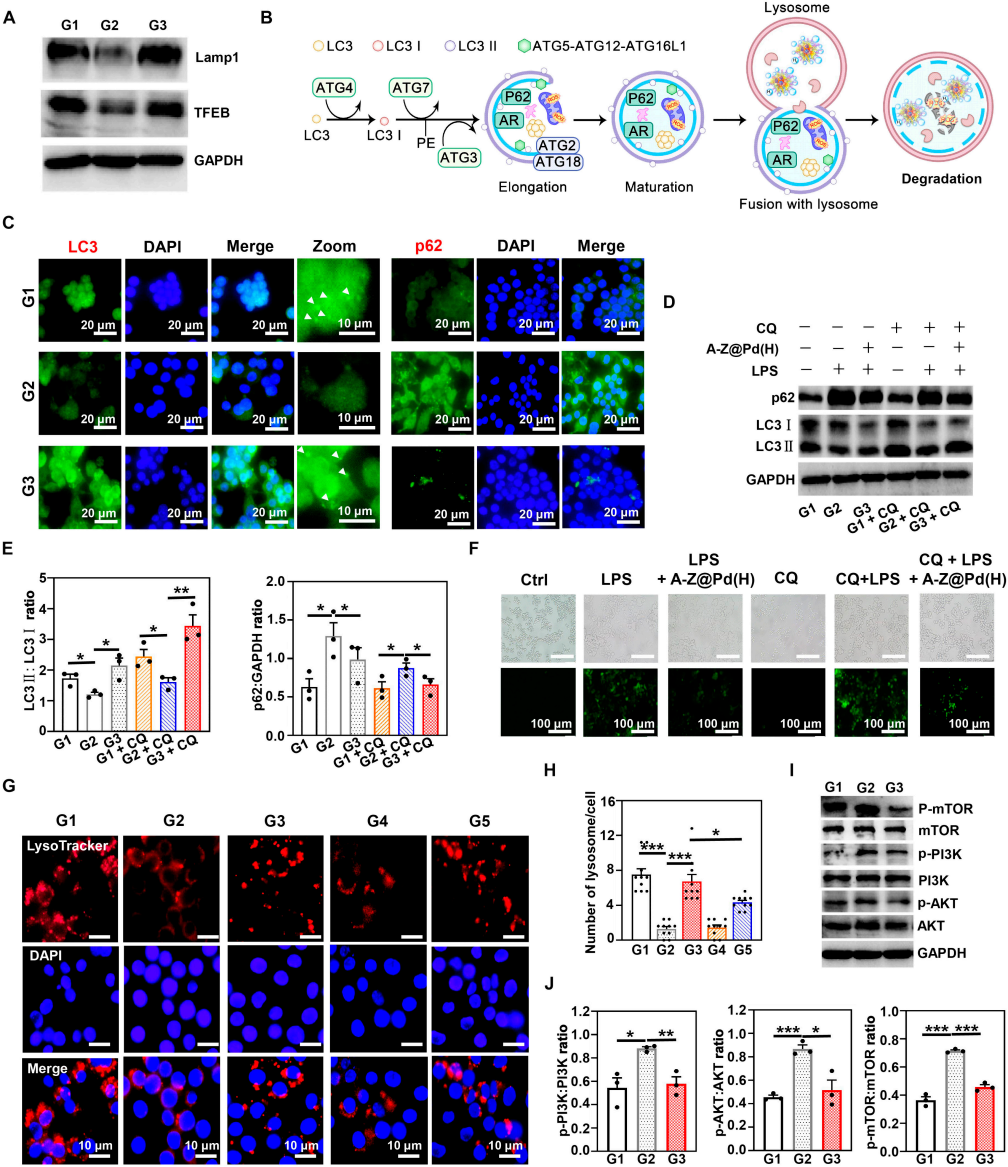
The enhancement effect of A-Z@Pd(H) on ROS degradation by autophagy–lysosome pathway in macrophages. (A) Expression level of TFEB and Lamp1 determined by WB. (B) Schematic diagram of the autophagy–lysosomal working system. (C) Effect of A-Z@Pd(H) on autophagy behavior of RAW264.7 cells detected by immunofluorescence staining (LC3 and p62). (D) WB detection of p62 and LC3 in RAW264.7 cells to explore the effect of A-Z@Pd(H) on autophagy behavior under the dual influence of LPS stimulation and lysosomal inhibition. (E) Quantitative analyses of the LC3II:LC3I ratio and p62 protein levels in (D). (F) ROS fluorescence images of RAW264.7 cells after different treatments. (G) Fluorescence images of lysosomes in different groups and (H) the lysosomal counting. (I) Protein levels in the PI3K/AKT/mTOR signaling cascade detected by WB and (J) the relative intensity quantification. G1: Ctrl; G2: LPS; G3: LPS + A-Z@Pd(H); G4: LPS + A-Z; G5: LPS + A-Z@Pd. Data are means ± SD (*n* ≥ 3). *P < 0.05, **P < 0.01, ***P < 0.001.

Autophagy is a lysosome-mediated degradation process that functions as an intrinsic cellular defense mechanism, facilitating the elimination of damaged or superfluous components in response to various stressors such as hypoxia and inflammation [45]. In an inflammatory environment, the excessive accumulation of ROS contributes to the impairment of cellular autophagy–lysosomal function, ultimately leading to cell death [46]. Western blot (WB) analyses of the LPS + A-Z@Pd(H) group demonstrated the increased protein expression of TFEB and Lamp1, indicating that A-Z@Pd(H) enhanced autophagy initiation and lysosomal function of RAW264.7 cells (Fig. 4A and Fig. S17) [47], thereby protecting the autophagy–lysosome pathway under inflammatory conditions. LC3 protein, known as MAP1LC3, plays a crucial role throughout the autophagic process and is a widely recognized autophagic marker [48]. p62 is a well-studied autophagic substrate, which acts as a bridge between LC3 and ubiquitinated proteins, eventually being enveloped together into autophagosomes for subsequent degradation by proteases (Fig. 4B) [49]. Therefore, the expression of the p62 protein is inversely correlated with autophagic activity. Immunofluorescence (Fig. 4C) and WB (Fig. S18) analyses revealed a decrease in LC3II expression and an increase in p62 expression of macrophages following LPS stimulation, bespeaking the autophagy dysfunction led by LPS. Lysosome inhibitor chloroquine (CQ), which changes the pH value of lysosomes, prevents the binding of autophagosomes and lysosomes [50]. The data indicated minimal differences in autophagy-related protein levels between the presence and absence of CQ, suggesting unobstructed intracellular autophagic flux (Fig. 4D and E). Treatment with A-Z@Pd(H) brought about a rescue of changes in LC3II and p62 expression levels, unveiling that A-Z@Pd(H) promoted cellular autophagic flux and could restore impaired lysosomal functions (Fig. 4C to E and Fig. S19). At the same time, the obstruction of ROS degradation caused by LPS and CQ was reversed by A-Z@Pd(H), demonstrating the possibility of A-Z@Pd(H) restoring intracellular ROS metabolism in an inflammatory environment (Fig. 4F). We further used a lysosomal red fluorescence probe to mark lysosomes within cells. As evidenced in Fig. 4G, the fluorescence intensity of lysosomes in macrophages stimulated with LPS decreased to some extent, while the LPS + A-Z@Pd(H) and LPS + A-Z@Pd groups had increased fluorescence intensity, with A-Z@Pd(H) showing more noticeable therapeutic effects (Fig. 4H). Finally, the PI3K/AKT/mTOR signaling cascade was explored, which was known as a negative feedback regulatory pathway for autophagy [51,52]. WB analyses of signaling molecules in this pathway showed that compared with the LPS group, the LPS + A-Z@Pd(H) group had reduced phosphorylation levels of PI3K, AKT, and mTOR proteins. These findings indicated that A-Z@Pd(H) treatment inhibited the PI3K/AKT/mTOR signaling cascade, consequently mitigating autophagy–lysosomal dysfunction in inflammatory conditions (Fig. 4I and J). Furthermore, TEM was employed to assess the cellular autophagic status (Fig. S20). Our findings indicated a significant increase in autophagolysosomes (single-membrane structures) in LPS-treated RAW264.7 cells, accompanied by the absence of autophagosomes (double-membrane structures), suggesting the impaired lysosomal degradation and blocked autophagic flux. However, following treatment with A-Z@Pd(H), autophagosomes were observed within the cells. Moreover, the reduced quantity of autolysosomes suggested an improvement in autophagic flux due to the alleviation of lysosomal dysfunction.

Lysosomal dysfunction has been implicated in various immune disorders, prompting the exploration of lysosomal modulation as a therapeutic avenue [53]. In inflammatory settings, lysosomes can become overwhelmed by the process of oxidative metabolites, impeding autophagic flux. We hypothesized that the location of A-Z@Pd(H) in the lysosome may help alleviate lysosomal stress. The results above demonstrated that A-Z@Pd(H) could restore impaired lysosomal function and promote autophagy–lysosomal metabolism to alleviate the excessive accumulation of intracellular ROS in the inflammatory environment.

### Effects of A-Z@Pd(H) on macrophage polarization

**Fig. 5.**
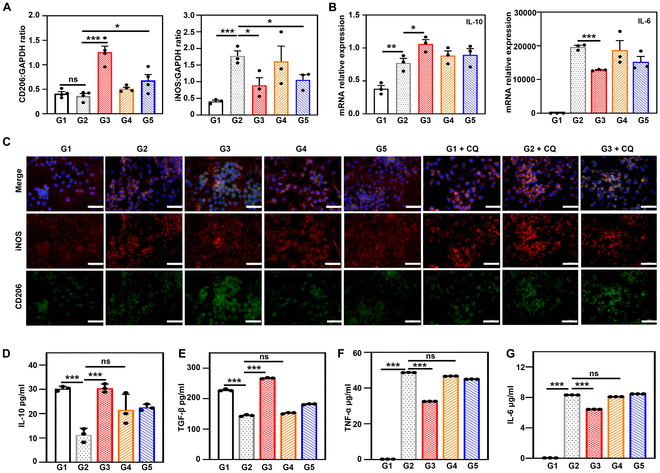
The regulation of macrophage polarization via A-Z@Pd(H). (A to C) Expression of inflammatory cytokines in RAW264.7 cells determined by (A) WB (CD206 and iNOS), (B) qRT-PCR (IL-10 and IL-6), and (C) immunofluorescence staining (CD206 and iNOS). Scale bar, 50 μm. (D to G) (D and E) Anti-inflammatory factors (IL-10 and TGF-β) and (F and G) inflammatory factors (TNF-α and IL-6) in the supernatants collected from different groups (G1 to G5 groups) assessed by ELISA. G1: Ctrl; G2: LPS; G3: LPS + A-Z@Pd(H); G4: LPS + A-Z; G5: LPS + A-Z@Pd. Data are means ± SD (*n* ≥ 3). *P < 0.05, **P < 0.01, ***P < 0.001. ns, no significance.

As an important link in macrophage resistance against inflammation, intracellular degradation of ROS affects the macrophage function [54,55]. An inflammatory microenvironment was effectively induced by LPS. Figure S21A shows the morphology of RAW264.7 cells in each group under an optical microscope. The quantitative real-time polymerase chain reaction (qRT-PCR), WB, and immunofluorescence methods were used to analyze the polarization state of RAW264.7 cells (Fig. 5A to C and Fig. S21B and C). All results demonstrated that compared with the LPS and LPS + A-Z@Pd groups, the LPS + A-Z@Pd(H) group exhibited down-regulation of M1-related pro-inflammatory proteins [interleukin-6 (IL-6), inducible nitric oxide synthase (iNOS), and tumor necrosis factor-α (TNF-α)] and up-regulation of M2-related anti-inflammatory proteins (IL-10 and CD206). The results indicated that A-Z@Pd(H) intervention promoted macrophage transformation from pro-inflammatory phase (M1 phenotype) to anti-inflammatory phase (M2 phenotype), with reductive hydrogen likely playing a major role [33]. To better understand the regulatory role of A-Z@Pd(H) in M2 polarization of macrophage, we stimulated RAW264.7 cells with IL-4 to induce M2 polarization and subsequently treated the cells with A-Z@Pd(H). Immunofluorescence (Fig. S22A) and qRT-PCR (Fig. S22B) results demonstrated that A-Z@Pd(H) up-regulated the expression of CD206. Furthermore, the expression level of the IL-10 gene was also increased. These findings provided evidence that A-Z@Pd(H) indeed promoted M2 polarization of macrophages. In addition, we observed increased expression of iNOS following CQ treatment (Fig. 5C), particularly in the G2 + CQ group where both LPS and CQ were administered. Subsequent treatment with A-Z@Pd(H) (G3 + CQ group) could decrease iNOS expression and slightly up-regulate CD206. This suggested that lysosomal damage may induce macrophage polarization toward M1 phenotype, which can be mitigated by A-Z@Pd(H) treatment. In addition, we assessed inflammatory and anti-inflammatory factors in macrophage culture medium following various treatments by enzyme-linked immunosorbent assay (ELISA). The LPS + A-Z@Pd(H) group showed higher levels of anti-inflammatory factors [IL-10 and transforming growth factor-β (TGF-β)] (Fig. 5D and E) and lower levels of pro-inflammatory factors (TNF-α and IL-6) (Fig. 5F and G) compared to the LPS group, thereby creating a more favorable osteogenic environment.

### Osteogenesis promotion and OC inhibition by A-Z@Pd(H)

Many studies have reported that ZIF-8 has the ability to direct mesenchymal stem cells toward an osteoblast lineage [36–38], which might be attributed to the release of Zn^2+^. We compared the osteogenic abilities of MC3T3-E1 cells respectively treated with ZIF-8 and A-Z@Pd(H) by alkaline phosphatase (ALP) staining, alizarin red S (ARS) staining, and qRT-PCR methods. qRT-PCR analyses displayed that runt-related transcription factor 2 (RUNX2), ALP, and osteocalcin (OCN) genes were up-regulated in both the ZIF-8 and A-Z@Pd(H) groups (Fig. S23A to C). ARS is the renowned marker for identifying the formation of mineralized nodules, and ALP plays a crucial role in accelerating the hydrolysis of pyrophosphates during mineralization [37,56]. Compared with the Ctrl group, the A-Z@Pd(H) group exhibited significantly enhanced ALP expression (Fig. S23D and E) and increased calcium mineralization nodules (red areas) (Fig. S23F and G) as the ZIF-8 group did. These results proved that A-Z@Pd(H) possessed the same osteogenic-promoting ability as ZIF-8.

**Fig. 6.**
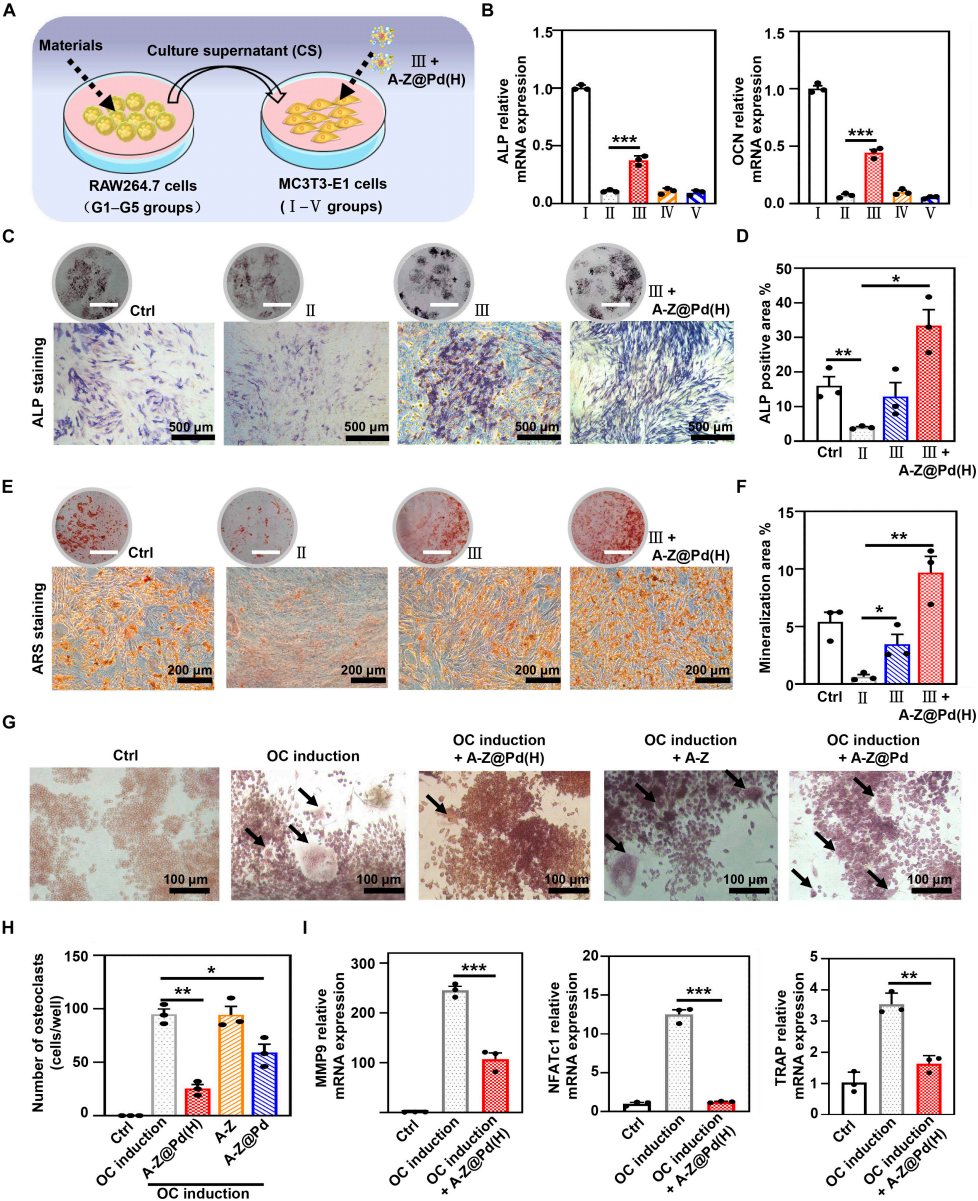
The reversing effect of A-Z@Pd(H) on osteoblast suppression and abnormal OC activation in inflammatory environments. (A) Schematic diagram of cell experiments on the effects of osteogenesis in an inflammatory environment. I, II, III, IV, and V groups represent osteoblasts cultured with osteoblast-inducing conditional medium mixed with different macrophage CSs collected from G1, G2, G3, G4, and G5 groups, respectively. G1: Ctrl; G2: LPS; G3: LPS + A-Z@Pd(H); G4: LPS + A-Z; G5: LPS + A-Z@Pd. (B) Effect of medium collected from different groups (G1 to G5 groups) of macrophages on osteoblast differentiation, detected by qRT-PCR for osteogenesis-related gene analyses including ALP (left) and OCN (right). (C to F) Osteogenesis activity of MC3T3-E1 cells in Ctrl, II, III, and III + A-Z@Pd(H) groups characterized by (C) ALP staining and (E) ARS staining. The circular picture on the upper side is an optical picture of the plate staining. Scale bar, 1 cm. (D) ALP and (F) ARS-positive area quantification. (G and H) (G) TRAP staining images and (H) quantitative statistics of OCs in the Ctrl group and OC induction group with different treatments. (I) qRT-PCR analyses of different OC-induced RAW264.7 cells for OC-specific genes, including MMP9, NFATc1, and TRAP. Data are means ± SD (*n* ≥ 3). *P < 0.05, **P < 0.01, ***P < 0.001.

To further investigate the impact of A-Z@Pd(H) intervention on osteogenesis in an inflammatory environment, the culture supernatant (CS) of LPS-treated macrophages cultured with different materials was collected and further incubated with MC3T3-E1 cells (Fig. 6A). It was found that compared with the LPS supernatant (CS from the LPS group), the A-Z@Pd(H) supernatant [CS from the LPS + A-Z@Pd(H) group] up-regulated the expression of ALP and OCN genes in MC3T3-E1 cells (Fig. 6B), enhanced ALP expression (Fig. S24A and B), and promoted more mineralized nodule formation (Fig. S24C and D). Therefore, A-Z@Pd(H) prevented the reduction of osteogenesis to some extent by alleviating inflammation, which might be related to the fact that macrophages treated with A-Z@Pd(H) predominantly appeared as M2 phenotype under inflammatory conditions. Notably, when A-Z@Pd(H) was further added to the CS from the LPS + A-Z@Pd(H) group and then applied as a component of the osteoblast culture medium [III + A-Z@Pd(H) group], the weakened osteogenic behavior was significantly reversed. ALP staining (Fig. 6C and D) and ARS staining (Fig. 6E and F) results showed that the highest mineralization level was presented in the III + A-Z@Pd(H) group. To further explore the comprehensive therapeutic effects of A-Z@Pd(H) in a multicellular environment, we conducted a Transwell coculture experiment and induced osteogenesis in MC3T3-E1 cells (Fig. S25). After 7 d, ALP staining results revealed that A-Z, A-Z@Pd, and A-Z@Pd(H) all promoted osteogenic differentiation under inflammatory conditions, with the A-Z@Pd(H) treatment group showing particularly notable effects. It was speculated that in addition to the reduction of macrophage inflammation levels by A-Z@Pd(H), the direct osteogenesis promotion of the materials themselves and the relief of ROS stress on osteoblasts were also favorable factors for A-Z@Pd(H) to promote osteogenesis in an inflammatory environment.

OC activity is closely associated with the onset and progression of OP. As important precursors of OCs, macrophages can differentiate into OCs under the stimulation of cytokines including the receptor activator of nuclear factor-κB ligand (RANKL) and macrophage colony-stimulating factor (M-CSF), which are up-regulated in the inflammatory environment [24,57]. As shown in Fig. 6G, many macrophages differentiated into large and multinucleated OCs under culturing of OC induction medium (OC induction, containing RANKL and M-CSF), whose cytoplasm was stained red by the tartrate-resistant acid phosphatase (TRAP) staining. In contrast, few OCs were in the OC induction + A-Z@Pd(H) group. The quantitative results in Fig. 6H validated the advantage of A-Z@Pd(H) in inhibiting OC formation. Furthermore, qRT-PCR analyses testified that OC-related genes, including matrix metalloproteinase 9 (MMP9), nuclear factor of activated T cells c1 (NFATc1), TRAP, c-fos, and cathepsin K (CTSK), were inhibited at the mRNA level by A-Z@Pd(H) (Fig. 6I and Fig. S26). These results indicated that A-Z@Pd(H) exerted an inhibitory effect on OC activity, thereby preventing excessive bone resorption, likely due to the reduced intracellular ROS levels [22–24].

A-Z@Pd(H) was engineered to modulate pivotal cells in the bone tissue microenvironment. Results from the cellular experiments demonstrated that, in addition to its role in regulating macrophage polarization, A-Z@Pd(H) directly impacted osteogenic and osteoclastic differentiation. Hence, this comprehensive therapeutic approach showed potential for effectively treating OP.

### Bone-targeting and Zn^2+^-releasing abilities of A-Z@Pd(H)

**Fig. 7.**
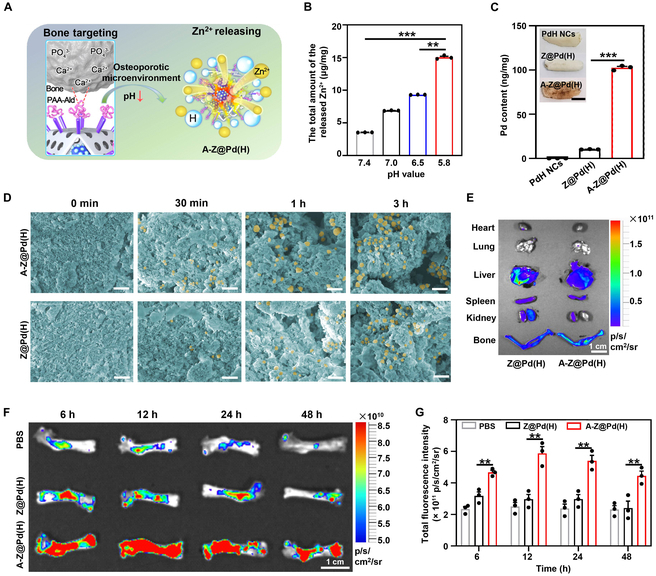
Bone-targeting and Zn^2+^-releasing properties of A-Z@Pd(H). (A) Schematic diagram of bone-targeting and Zn^2+^ releasing of A-Z@Pd(H) in an osteoporotic environment. (B) Amount of Zn^2+^ released by A-Z@Pd in solutions at different pH. (C) Images of mouse tibial bone fragments and their adsorbed Pd content after immersion in PdH NCs, Z@Pd(H), and A-Z@Pd(H) solutions. Scale bar, 1 cm. (D) Representative SEM images (pseudo-color) of A-Z@Pd(H) and Z@Pd(H) surface interactions with HAP after different time intervals. Scale bar, 1 μm. (E) Fluorescence imaging results of mouse major organs after intravenous injection for 6 h. (F) Fluorescence imaging results of femur after intravenous injection for 6, 12, 24, and 48 h in the different groups and (G) the quantitative results. Data are means ± SD (*n* ≥ 3). *P < 0.05, **P < 0.01, ***P < 0.001.

Efficiently targeting therapeutic nanomaterials to bone tissue is a critical requirement for ensuring precise and effective treatment of OP (Fig. 7A). Abundant hydrogen ions (H^+^) secreted by mature OCs create an acidic environment (pH ~ 4) on the bone surface, which promotes the degradation of the organic matrix by CTSK and TRAP, ultimately leading to OP [56]. As an acid-responsive disintegration material, ZIF-8 could provide targeted and enhanced zinc ion therapy for areas of severe bone destruction [36–38]. Inductively coupled plasma mass spectrometry (ICP-MS) confirmed that as the pH value of the solution decreased, the amount of released Zn^2+^ increased (Fig. 7B), which was expected to promote osteogenesis.

The bone-targeting properties of the materials were investigated both in vitro and in vivo. Ald is a potent and commonly utilized bone-targeting agent due to its strong attraction to calcium ions (Ca^2+^) in the bone microenvironment [22,24,40], which endowed A-Z@Pd(H) with the ability to target systemic bone tissue. Tibias removed from mice were incubated with A-Z@Pd(H), and the bone turned from white to brownish yellow after 12 h, while there was no significant change in the Z@Pd(H) and PdH NC groups (Fig. 7C). Further ICP-MS analyses of the bone samples revealed that a Pd element content in the A-Z@Pd(H) group was as high as 100 ng/mg. Hydroxyapatite (HAP), the predominant mineral in bone tissues, was selected to mimic the bone microenvironment [40]. After co-incubation with different nanomaterials, HAP adsorbed more A-Z@Pd(H) fixed to the surface than Z@Pd(H) at different times (Fig. 7D and Fig. S27). All the above results suggested that A-Z@Pd(H) had a high affinity to bone in vitro. Afterward, we evaluated the in vivo bone-targeting ability of A-Z@Pd(H). Following intravenous administration into the tail vessels of mice, all tissues including hearts, livers, spleens, lungs, kidneys, and bones were extracted at different time points for the in vivo imaging system (ABL X6, Tanon) examination. In Fig. 7E, it could be observed that A-Z@Pd(H) exhibited preferential accumulation in bone tissues instead of other organs. Moreover, the fluorescence intensity of A-Z@Pd(H) in bone tissue was significantly higher than that of Z@Pd(H), peaking at 12 h and gradually diminishing thereafter (Fig. 7F and G). These findings offered insights for determining dosing frequency in subsequent animal studies.

### The comprehensive therapeutic effect of A-Z@Pd(H) in OP

**Fig. 8.**
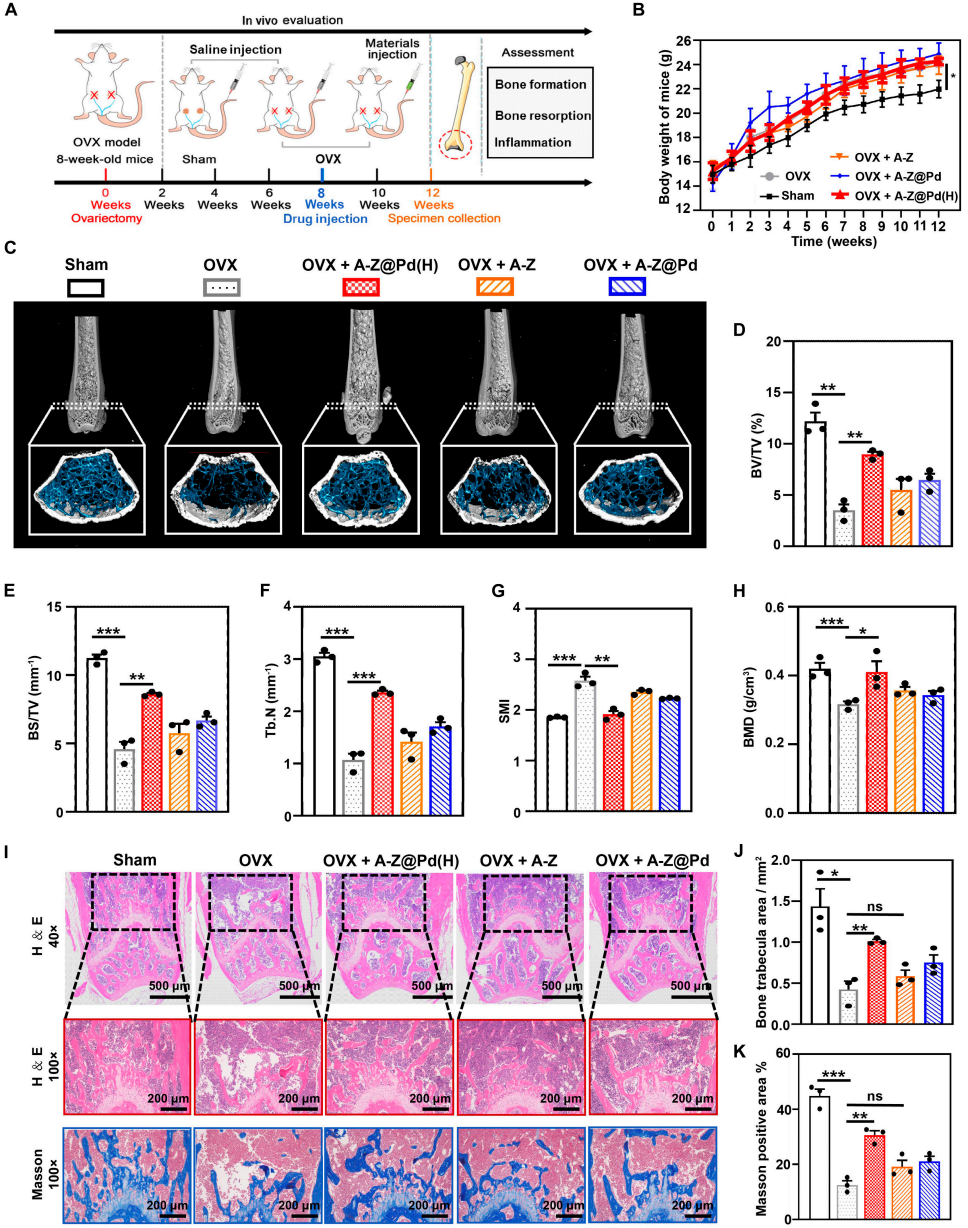
The comprehensive therapeutic effects of A-Z@Pd(H) on the OP mice. (A) Schematic illustration of experimental design in animals to evaluate the anti-OP efficacy of materials. (B) Weight changes of mice treated with different agents during 12 weeks. (C) Micro-CT images of the femora treated with different agents. (D to H) Quantitative analyses of BV/TV, BS/TV, Tb.N, SMI, and BMD of each group. (I) H&E and Masson staining showing the histomorphology change in the femora treated with different agents. (J) Quantifications of bone trabecular area in each group of mice. (K) Quantifications of Masson-positive area in each group of mice. Data are means ± SD (*n* ≥ 3). *P < 0.05, **P < 0.01, ***P < 0.001.

An ovariectomized murine model of OP (OVX model) was established to further investigate the therapeutic role of A-Z@Pd(H) in vivo. The experimental design was depicted in Fig. 8A. Mice with ovaries preserved were established as the Sham group. The OVX mice were divided into 4 groups [OVX group, OVX + A-Z@Pd(H) group, OVX + A-Z group, and OVX + A-Z@Pd group]. Significant weight gain was observed in OVX mice compared to the mice in the Sham group, with no significant differences among the treatment groups (Fig. 8B). All mice were sacrificed at the end of the treatment course, and the comparison of uterine size and weight revealed that OVX mice possessed shrunken and lighter uteruses with no significant changes after treatment (Fig. S28). These observations implied the success of OVX model creation [58].

Parameters including bone volume per tissue volume (BV/TV), bone surface per tissue volume (BS/TV), trabecular number (Tb.N), and bone mineral density (BMD) obtained using microcomputed tomography (micro-CT) were found to decrease in the OVX group compared with parameters in the Sham group, while the structure model index (SMI) parameter increased, indicating evidential bone loss in OVX model mice (Fig. 8C to H). Treatment with A-Z@Pd(H) significantly reduced bone loss, increased bone trabecula density, and preserved trabecular structure in ovariectomized mice. Specifically, BV/TV (8.95 ± 0.46% versus 3.51 ± 1%), BS/TV (8.61 ± 0.16 mm^−1^ versus 4.58 ± 0.93 mm^−1^), Tb.N (2.36 ± 0.06 mm^−1^ versus 1.07 ± 0.20 mm^−1^), and BMD (0.41 ± 0.05 g/cm^3^ versus 0.32 ± 0.01 g/cm^3^) significantly increased in the OVX + A-Z@Pd(H) group compared with the untreated OVX group. On the other hand, A-Z@Pd seemed to have a mild therapeutic efficacy, but its effect was weaker than that of A-Z@Pd(H), suggesting that reductive hydrogen played a pivotal role in the treatment of OP. Consistently, hematoxylin and eosin (H&E) staining and Masson staining designated that A-Z@Pd(H) largely restored bone volume and structure in ovariectomized mice (Fig. 8I to K).

**Fig. 9.**
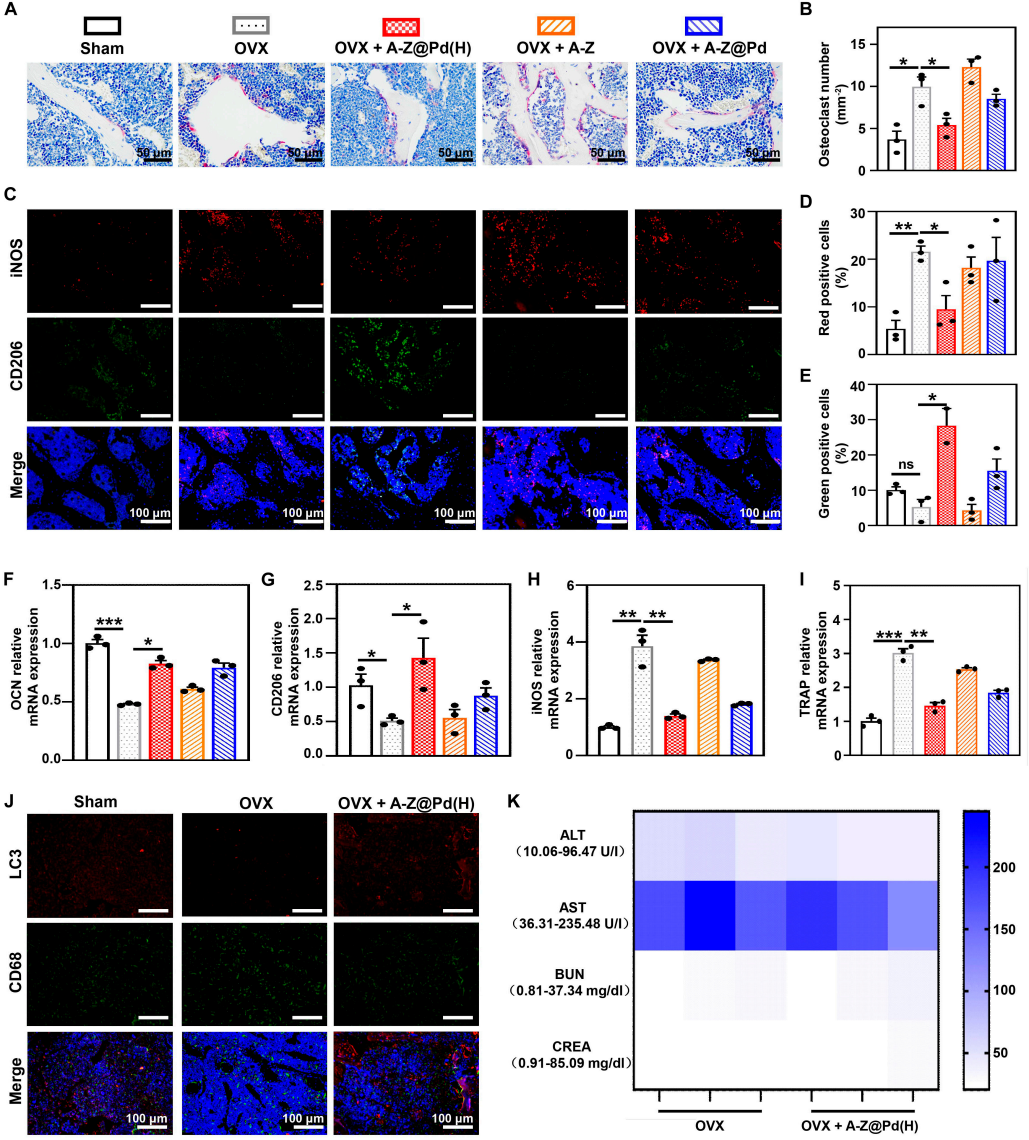
Histological assessment of bone resorption and inflammation levels of bone tissue. (A) TRAP staining of the femur sections collected from different groups. (B) Quantitative statistics of OCs based on the TRAP staining. (C) Immunofluorescence staining images of bone sections stained by iNOS (in red) and CD206 (in green). (D and E) Quantifications of (D) iNOS and (E) CD206-positive cells in femur sections. (F to I) Relative mRNA expression of OCN, TRAP, iNOS, and CD206 in each group. (J) Immunofluorescence staining images of bone sections stained by LC3 (in red) and CD68 (in green). (K) Serum levels of ALT, AST, BUN, and CREA of mice in OVX and OVX + A-Z@Pd(H) groups at postoperative week 12. Data are means ± SD (*n* ≥ 3). *P < 0.05, **P < 0.01.

According to TRAP staining, mice in the OVX group showed a higher count of OCs (TRAP-positive cells), while A-Z@Pd(H) treatment remarkably inhibited OC formation (Fig. 9A and B), which was consistent with the in vitro results. Immunofluorescence staining of bone sections unveiled a significant decrease in iNOS-positive areas in OVX mice that received A-Z@Pd(H) administration compared with the untreated and other treatment groups (Fig. 9C and D), while the immunofluorescence staining and quantification results of the CD206-positive area proved that A-Z@Pd(H) exhibited a significant up-regulation effect of CD206 (Fig. 9C and E), which further illustrated the anti-inflammatory induction capability of A-Z@Pd(H). Subsequently, qRT-PCR analyses of bone tissues indicated the up-regulation of osteogenesis-related gene (OCN) and anti-inflammatory factor (CD206), as well as down-regulation of pro-inflammatory factor (iNOS) and OC-related gene (TRAP) in the A-Z@Pd(H) treatment group (Fig. 9F to I). For the evaluation of autophagy levels in bone tissue, we conducted immunofluorescence double staining experiments targeting LC3 and CD68 (Fig. 9J). Our findings revealed that the LC3 fluorescence intensity in the OVX + A-Z@Pd(H) group exceeded that of the OVX group, alongside an elevated colocalization fluorescence density with CD68 (Fig. S29). These results collectively suggested that treatment with A-Z@Pd(H) augmented cellular autophagic activity, particularly within macrophages.

Overall, in animal experiments, A-Z@Pd(H) was found to reverse OP by promoting osteogenesis, inhibiting OC activity, and reducing inflammation. Additionally, increased autophagic activity at the tissue level was observed. Finally, we assessed the material’s biocompatibility in animal models. H&E staining affirmed no distinct pathological changes in major organs (hearts, livers, spleens, lungs, and kidneys) of mice treated with A-Z@Pd(H) (Fig. S30), and the hematological parameters including alanine transaminase (ALT), aspartate transaminase (AST), blood urea nitrogen (BUN), and creatinine (CREA) were listed among the normal expression levels (Fig. 9K). To gain further insight into the long-term in vivo degradation behavior of A-Z@Pd(H) following extended treatment, we conducted ICP-MS analyses (Table S1). Specifically, we investigated the distribution of noble metal Pd elements in various organs of mice. The results indicated that following treatment, there was a deposition of Pd in low quantities within bone tissue, while Pd content was undetected in other vital organs. The above detection results demonstrated the good compatibility and negligible side effects of A-Z@Pd(H) in the OP treatment.

## Conclusion

In conclusion, we have pioneered a bone-targeted nanocomposite, A-Z@Pd(H), designed for hydrogen delivery and zincum repletion throughout the bone microenvironment. Reductive hydrogen enhances the ROS-scavenging capacity of Pd nanoenzymes, offering a novel approach for ROS-targeted OP treatment. A-Z@Pd(H) alleviates lysosomal stress in inflammation, thereby restoring autophagic flux and balancing intracellular ROS metabolism. This leads to macrophages polarizing toward the M2 phenotype and a decrease in OC differentiation. Additionally, diverging from the singular anti-osteoclastic effect of conventional anti-osteoporotic drugs, this system remodels the osteoporotic microenvironment through immune regulation, osteogenesis promotion, and OC inhibition, thereby providing a comprehensive therapeutic strategy. However, further investigations are needed to assess the A-Z@Pd(H)’s effects on coculture systems involving macrophages, osteoblasts, and OCs. Additionally, there is a need for future research in medical hydrogen storage materials to enhance hydrogen storage capacity, thereby reducing administration frequency. Moreover, the long-term biosafety and metabolism of A-Z@Pd(H) warrant extensive study. To sum up, this system composed of hydrogen therapy and ion therapy provides a comprehensive approach to treating OP, and shows promise for treating other immune-related tissue-damaging diseases such as periodontitis [42], rheumatoid arthritis [59], and various brain degenerative diseases [60,61].

## Materials and Methods

### Materials

Sodium tetrachloropalladate (Na_2_PdCl_4_), l-ascorbic acid (AA), polyvinyl pyrrolidone [PVP; molecular weight (*M*_w_) 58,000], potassium bromide (KBr), 2-methylimidazole (2-mlm), and zinc nitrate hexahydrate [Zn(NO_3_)_2_·6H_2_O] were purchased from Macklin Biochemical Co. Ltd. (Shanghai, China). Sodium borohydride (NaBH_4_) was purchased from Aladdin Biochemical Technology Co. Ltd. (Shanghai, China). Ald sodium trihydrate and PAA (*M*_w_ ≈ 2,000) were purchased from Acros (Belgium). 4-(4,6-Dimethoxy-1,3,5-triazin-2-yl)-4-methylmorpholinium chloride (DMTMM; 97%) was purchased from J&K Scientific Co. Ltd. (Beijing, China). 2,7-DCFH-DA, CCK-8 kit, live/dead cell staining kit, and TRAP staining kit were obtained from Solarbio Technology Co. Ltd. (Beijing, China). ARS and ALP staining kits were purchased from Beyotime Co. Ltd. (Shanghai, China). Fetal bovine serum (FBS) and Dulbecco’s modified Eagle’s medium (DMEM) were bought from HyClone (Beijing, China).

### Synthesis of Z@Pd nanoparticles

Pd NCs were initially prepared according to previously reported methods [39]. In brief, 301.0 mg of KBr, 60.0 mg of AA, and 106.4 mg of PVP were dissolved in 8 ml of deionized (DI) water and stirred in 80 °C water bath for 15 min. Then, 3 ml of solution containing 56.3 mg of Na_2_PdCl_4_ was slowly added and continuously stirred at 80 °C for 3 h. The mixture was washed 3 times using an ultrafiltration centrifuge tube (molecular weight cutoff, 100 KPa). The obtained product (Pd NCs) was dispersed in 10 ml of DI water. Subsequently, 2 ml of Pd NC solution was mixed with 2.8 ml of solution containing 200 mg of Zn(NO_3_)_2_·6H_2_O, and the mixture was magnetically stirred for 10 min. Next, 8 ml of DI water containing 2 g of 2-mlm was quickly added to the above solution and continuously stirred for 10 min. The product was washed 3 times by centrifugation at 8,000 rpm to obtain Z@Pd. ZIF-8 was synthesized in a similar way, except that the Pd NC solution was replaced by DI water.

### Synthesis of A-Z@Pd nanoparticles

For the conjunction of Ald and PAA, 4 mg of PAA and 130 mg of Ald were dissolved in 200 ml of borate buffer (0.1 M, pH 8.5). Following 10 min of stirring, 110 mg of DMTMM in 40 ml of borate buffer solution (0.1 M, pH 8.5) was added, and the pH was adjusted to 7.5 using 2 M NaOH solution. The mixture was stirred for 24 h. The resultant product was purified via dialysis and lyophilized, yielding a white foam-like product (PAA-Ald). To coat Z@Pd with PAA-Ald, 20 mg of PAA-Ald was introduced into 100 ml of DI water containing 100 mg of Z@Pd. After stirring overnight, the mixture underwent centrifugation, followed by washing with DI water and finally vacuum-drying, leading to the formation of a black powder (A-Z@Pd).

### Synthesis of A-Z@Pd(H) nanoparticles

The A-Z@Pd solution was prepared by dissolving 50 mg of A-Z@Pd nanoparticles in 25 ml of DI water and utilizing ultrasonic dispersion. Then, 2 ml of Pd@ZIF-8 solution was injected into a 10-ml Schering bottle and sealed with a rubber plug. NaBH_4_ (1 g) was put into another Schering bottle and sealed with rubber plug. Two Schering bottles were connected with the capillary. The needle was inserted into the first bottle to connect the atmosphere. Then, 1 ml of dilute sulfuric acid solution (pH 3) was injected into the second bottle to produce hydrogen, and then hydrogen was pumped into A-Z@Pd solution for 20 min. The prepared A-Z@Pd(H) solution was sealed and kept at 4 °C for further experiments.

### Characterization

The morphology and elemental composition of all synthesized samples were examined by SEM (Zeiss/Sigma300, Japan), TEM (Tecnai G2 20, Thermo, USA), and x-ray energy-dispersive spectroscopy (EDS). N_2_ adsorption/desorption and pore size distribution were analyzed using a BSD-PM1/2 apparatus. FTIR spectra analyses were conducted on an FTIR spectroscopy (Nicolet 5700, Nicolet, USA). Zeta potential and size distribution of samples were assessed using the Zetasizer equipment (Malvern, UK). UV–vis absorption spectra were collected on a UV-2600 spectrophotometer (Shimadzu, Japan). Absorbance values were measured by the microplate reader of VICTOR Nivo 3S (PerkinElmer, UK). The stained cells were analyzed with live cell imaging system (Olympus IX83, Olympus, Japan).

### Release of reductive hydrogen in A-Z@Pd(H)

MB was used to detect the releasing of reductive hydrogen in A-Z@Pd(H). The standard curve of MB at 625 nm was first determined, which could quantify the release amount of reductive hydrogen. The HRW was set as the comparison group, which was obtained by an intelligent multifunctional water mechanism (V8, China). Then, 20 μl of A-Z@Pd(H) solution (1 mg/ml) and HRW were added to 3 ml of MB solution (10 μg/ml), respectively. The absorption peak of MB was measured in real time by a UV-2600 spectrophotometer.

### ROS-scavenging capability of A-Z@Pd(H) and A-Z@Pd

The total antioxidant ability was assessed through the typical ABTS and DPPH radical-scavenging assays. A-Z@Pd(H) at final concentrations of 0, 12.5, 25, 50, 100, and 200 μg/ml was incubated with DPPH (0.5 mM) in ethanol at 37 °C for 30 min. The absorption of the reaction mixtures at 517 nm was measured for colorimetric monitoring. For the ABTS assay, ABTS solution (7 mM) was pretreated with potassium persulfate (2.45 mM) overnight to generate ABTS radicals. A-Z@Pd(H) at final concentrations of 0, 12.5, 25, 50, 100, and 200 μg/ml was added to the ABTS radical solutions. After 10 min, the absorption of ABTS radicals was measured at 734 nm.

The removal of •OH by ZIF-8, A-Z@Pd, and A-Z@Pd(H) was evaluated using ESR (Bruker EMXplus, Germany). •OH radicals were generated using a TiO_2_/UV system under 340-nm UV light. Various materials were added to the aforementioned mixture and incubated for 15 min, followed by capture of the remaining •OH using dimethyl pyroline oxide (DMPO).

O_2_^•−^ scavenging capacity was assessed through nitrotetrazolium blue chloride (NBT) photoreduction assay. A solution was prepared containing 75 μM NBT, 12.5 mM methionine, 20 μM riboflavin, and various concentrations of samples (0, 25, 50, 100, and 200 μg/ml) in 25 mM phosphate-buffered saline (PBS; pH 7.4). The mixtures were subsequently subjected to 15 min of UV irradiation. After illumination, the reduction of NBT by O_2_^•−^, with an absorption peak at 560 nm, was quantified using UV–vis absorption spectroscopy. Negative and positive controls were included without and with UV illumination, respectively. All experiments were conducted in the dark. The inhibition ratio of O_2_^•−^ was calculated by the following formula:

$$\text{Inhibition ratio}\;\left(\%\right)=\frac{A_{p}\;-\;A_{0}}{A_{p}\;-\;A_{n}}\times 100\%$$ (1)

where *A*_*0*_, *A*_*n*_, and *A*_*p*_ represent the absorbances of the treated samples, negative control, and positive control, respectively. Additionally, ESR signals were employed to assess the scavenging activity of ZIF-8, A-Z@Pd, and A-Z@Pd(H) toward O_2_^•−^.

For the evaluation of H_2_O_2_ scavenging capability, samples at final concentrations of 0, 25, 50, 100, and 200 μg/ml were incubated with 20 mM H_2_O_2_ at 37 °C for 20 min. The residual H_2_O_2_ concentration after reaction was determined using a hydrogen peroxide assay kit (Solarbio, China). In addition, ESR signals were recorded to detect H_2_O_2_ scavenging activity of ZIF-8, A-Z@Pd, and A-Z@Pd(H). O_2_ generated during the process was visually captured using a digital camera and measured quantitatively by a dissolved oxygen meter (AR8406, Xima, China).

### Cytotoxicity evaluation of A-Z@Pd(H)

To comprehensively assess biocompatibility, the effects of A-Z@Pd(H) on the viability of mouse embryo osteoblast precursor cells (MC3T3-E1 cells), mouse leukemia cells of monocyte macrophage (RAW264.7 cells), and HUVECs were investigated by CCK-8 assay and live/dead cell staining. Briefly, cells were plated in 96-well plates (2 × 10^3^ cells per well) and incubated for 24 h. Subsequently, they were exposed to varying concentrations of A-Z@Pd(H) (0, 2.5, 5, 10, and 20 μg/ml). Cell viability was then assessed for 1, 2, and 3 d by CCK-8 assays. Additionally, living/dead cell staining experiment was also carried out. Cells subjected to different treatments were stained with calcein-acetoxymethyl ester/propidium iodide (calcein-AM/PI), and fluorescent microscopy was employed to capture images of the cells.

### Hemolysis assay

To isolate RBCs, fresh rat blood underwent centrifugation at 1,500 rpm for 15 min, followed by gentle washing and dilution with saline solution. Subsequently, a 100-μl solution of RBCs was added into 1.1 ml of A-Z@Pd(H) solution at concentrations of 62.5, 125, 250, 500, and 1,000 μg/ml. Another 100 μl of RBCs solution was added into 1.1 ml of saline solution as the negative control and DI water as the positive control. Following a 3-h incubation at 37 °C, supernatants were obtained by centrifuging at 1,500 rpm for 15 min. Then, the absorbance of each sample was measured at 540 nm using a microplate reader. The hemolysis rate was calculated using the following equation:

$$\text{Hemolysis ratio}\;\left(\%\right)=\frac{OD_{x}\;-\;OD_{s}}{OD_{w}\;-\;OD_{s}}\times 100\%$$ (2)

where *OD*_*x*_ is the optical density (OD) value of the A-Z@Pd(H) group, *OD*_*s*_ is the OD value of the negative control group, and *OD*_*w*_ is the OD value of the positive control group.

### Cellular internalization of A-Z@Pd(H)

RAW264.7 cells were cultured in 12-well plates for 12 h. Following this, 20 μg/ml FITC-labeled A-Z@Pd(H) was incubated with cells for 0, 0.5, 3, and 12 h, respectively. Afterward, the cells were treated with 4% paraformaldehyde for 15 min, followed by permeabilization with 0.1% Triton X-100 for 15 min. The early endosome marker (EEA1), late endosome marker (CD63), and Golgi marker (TGN38) were incubated. After 3 times of PBS cleaning, CoraLite594-conjugated Goat Anti-Mouse IgG(H+L) was incubated. Finally, after 3 times of PBS cleaning, cells were stained with 4′,6-diamidino-2-phenylindole (DAPI), sealed with coverslips, and shot by a microscope. Lysosomal fluorescent probes (LysoTracker) were used to locate and count lysosomes, which were then imaged by a live cell imaging system.

### Effects on macrophage polarization

To investigate the modulation effects of A-Z@Pd(H) on macrophage polarization, LPS-stimulated RAW264.7 cells were cultured with various nanomaterials. The polarization status (M1/M2) of RAW264.7 cells was measured through qRT-PCR, WB, and immunofluorescence methods. RAW264.7 cells were plated in 6-well plates (1 × 10^6^ cells per well) and cultured for 24 h. Subsequently, cells were treated with 20 μg/ml of different samples along with 1 μg/ml of LPS for 24 h. Total RNA was extracted using Trizol reagent (Beyotime, China), followed by reverse transcription to obtain cDNA. The expression levels of relevant inflammatory genes (TNF-α, IL-6, and IL-10) were quantified by qRT-PCR. Primer sequences were provided in Table S2. Meanwhile, the culture medium was collected and centrifuged (2,000 rpm, 20 min) to obtain supernatant for ELISA to measure TNF-α, IL-6, IL-10, and TGF-β levels. The protein expression levels of macrophage polarization markers (iNOS and CD206) were evaluated using immunofluorescence and WB methods. Additionally, IL-4 (20 ng/ml) was employed to stimulate the M2 polarization of macrophages, for further investigating the material’s role in this process. Specifically, RAW264.7 cells were stimulated with 20 ng/ml IL-4 for 24 h for M2 macrophage polarization. During polarization, macrophages were further administered with 20 μg/ml A-Z@Pd(H) to assess A-Z@Pd(H) treatment on M2 polarization.

### Intracellular ROS-scavenging activity

RAW264.7 cells were plated in 24-well plates (5 × 10^5^ cells per well) and cultured for 24 h. Subsequently, they were exposed to a high-glucose medium supplemented with LPS (1 μg/ml) and different nanomaterials [A-Z@Pd(H), A-Z, and A-Z@Pd]. To observe the effects of the material itself, a group with only A-Z@Pd(H) treatment and without LPS stimulation was set up. What is more, a group of lysosomal inhibitors (CQ) was established to evaluate the nanomaterials’ potential to restore lysosomal function and achieve ROS clearance. Subsequent to a 24-h incubation period, the culture medium was substituted with serum-free medium containing DCFH-DA, which reacts with ROS to produce fluorescent 2,7-dichlorofluorescein. This mixture was incubated with the cells for 30 min, after which cellular fluorescence was examined using an inverted fluorescence microscope (excitation: 488 nm, emission: 525 nm). Additionally, for the early (12 h) generation of ROS and organelle colocalization, we simultaneously employed mitochondrial and lysosomal probes to investigate organelle localization and observed them under confocal microscopy.

### Effects on autophagy–lysosomal function in macrophages

RAW264.7 cells were plated in 6-well plates (5 × 10^6^ cells per well) and cultured for 24 h. In order to determine the effect of LPS (1 μg/ml) on the autophagy function of macrophages, the cells were stimulated with LPS for different times (1, 3, 6, 12, and 24 h). After that, the expressions of autophagy-associated proteins (TFEB, P62, and LC3) and inflammatory factor (iNOS) in cells were analyzed by WB. After further intervention with A-Z@Pd(H) (20 μg/ml), autophagy–lysosome associated proteins (Lamp1, TFEB, LC3, and P62) in cells were detected by WB and immunofluorescence. To learn more about the intervening role of materials in the state of lysosomal function inhibition, some cells were pretreated by CQ (10 μM) for 2 h. Whereafter, a lysosome probe was used to confirm the lysosomal situation of each group. Briefly, the cells were incubated with LysoTracker working solution at 37 °C for 1 h and photographed by a live cell imaging system after the unbound probe was removed. At last, the expression of related proteins in PI3K/AKT/mTOR signaling cascade and their phosphorylation levels were detected through the WB method. Furthermore, TEM was employed to investigate the regulatory effects of A-Z@Pd(H) on autophagy.

### Evaluations of osteogenic activity

To investigate osteogenic differentiation, MC3T3-E1 cells were incubated with different nanoparticles [A-Z@Pd(H) and ZIF-8] in a standard medium supplemented with 10 mM sodium β-glycerophosphate, 50 ng/ml AA, and 10 nM dexamethasone for 7 and 14 d. The osteogenic induction medium was refreshed every 3 d during the experiment. After 7-d coculture, cells were fixed with 4% paraformaldehyde, washed with PBS, and stained using ALP staining kit. Images were captured under an inverted microscope, and the area of ALP-positive staining was quantified using ImageJ software (Bethesda, USA). Following 14 d of treatments, cells were stained using ARS staining solution. Images were taken with a microscope, and semiquantitative analyses of osteogenic effectiveness were conducted using ImageJ software. Additionally, cells from different treatments were collected to assess the expression levels of osteogenetic genes, including RUNX2, ALP, and OCN by qRT-PCR. Primer sequences were detailed in Table S2.

To investigate the impact of the inflammatory environment on osteogenesis, an osteogenic differentiation medium containing pro-inflammatory factors was prepared as previously described. The medium supernatant from LPS-stimulated RAW264.7 cells with or without nanomaterial treatment was collected by centrifugation (2,000 rpm, 20 min) and subjected to further high-speed centrifugation (10,000 rpm, 20 min) to eliminate the nanomaterials, cell debris, and impurities, resulting in the acquisition of the final supernatant. Subsequently, the supernatant was mixed with the normal medium at a ratio of 1:1 and supplemented with osteogenic differentiation induction reagent (50 ng/ml AA, 10 mM sodium β-glycerophosphate, and 10 nM dexamethasone) to prepare the conditioned osteogenic induction medium. MC3T3-E1 cells were cultured in different conditioned medium described above for 10 d, and osteoblastic activity of the cells was also detected by ALP and ARS staining. In addition, the expression levels of osteogenic genes were measured via qRT-PCR. To better illustrate the effect of A-Z@Pd(H) on osteogenic behavior in an inflammatory environment, the culture medium supernatant from LPS-stimulated RAW264.7 cells with A-Z@Pd(H) treatment and 20 μg/ml A-Z@Pd(H) co-intervene in osteogenic differentiation, and then ALP as well as ARS staining methods were used to analyze osteoblastic viability of cells at 10 d.

A Transwell coculture system was employed to further explore the comprehensive therapeutic effects of the materials on a multicellular environment. RAW264.7 cells (4 × 10^4^/ml) were seeded in the upper compartments of the Transwell system, while the MC3T3-E1 cells (4 × 10^4^/ml) were placed in the lower chambers of the system. LPS (1 μg/ml) or the materials (20 μg/ml) were introduced into the coculture system once cells were transferred to osteogenic medium containing 10 mM sodium β-glycerophosphate, 50 ng/ml AA, and 10 nM dexamethasone. The culture medium was refreshed every 2 d, and osteoblastic viability of MC3T3-E1 cells was analyzed at 7 d using ALP staining methods.

### Evaluations of OC activity

RAW264.7 cells were plated in a 96-well plate (2,000 cells per well). Following a 24-h incubation, the medium was replaced with an OC induction medium containing 50 ng/ml RANKL (R&D Systems, USA) and 25 ng/ml M-CSF (R&D Systems, USA). In different groups, the OCs were treated with different medium for 24 h including normal medium, OC induction medium, OC induction medium + A-Z@Pd(H), OC induction medium + A-Z, and OC induction medium + A-Z@Pd. TRAP staining was performed after 5 d of culture. After washing with PBS, the cells were fixed with 4% paraformaldehyde for 30 min, and the operation was performed according to the manufacturer’s instruction of TRAP staining kit. TRAP-positive OCs (with wine-red cytoplasm) were microscopically examined. The expression levels of OC-related genes (MMP9, NFATc1, TRAP, c-fos, and CTSK) were measured using the qRT-PCR method.

### In vitro and in vivo bone targeting

The binding capability of A-Z@Pd(H) to bone mineral was compared to that of nontargeted Z@Pd(H). Solutions of 1 mg/ml A-Z@Pd(H) and Z@Pd(H) were incubated with 20 mg of HAP microparticles in a 5-ml solution and stirred for varying durations (0 min, 30 min, 1 h, and 3 h). Following the incubation period, the solution was centrifuged to separate the HAP particles and the nanoparticles that bound to them. The precipitate was rinsed repeatedly with DI water and dried in an oven for SEM analyses. The binding ability of A-Z@Pd(H) to bone fragments was further assessed using the same method for the tibia of mice. The tibia was removed from the healthy mice, and then the tibial surface muscle and fascia were removed. The tibia was incubated with 2 ml of A-Z@Pd(H), Z@Pd(H), or PdH NC solution for 24 h. After that, the tibia was washed and then air dried. Finally, the Pd content in each group of tibia was measured by ICP-MS.

For in vivo bone targeting, rhodamine B-labeled A-Z@Pd and Z@Pd were injected intravenously in mice. After 12 h, the different organs, including hearts, livers, spleens, lungs, kidneys, and femurs, were taken out for in vivo imaging system examination. After different time intervals (6, 12, 24, and 48 h), femurs of different groups were collected for comparison of fluorescence intensity. Three mice in each group were used for the statistical analyses. The excitation wavelength was set at 555 nm with an emission wavelength of 580 nm.

### In vivo OP inhibition

The in vivo experiments were conducted in strict accordance with laboratory animal care guidelines and approved by the ethics committee of Nanchang University (Nanchang, China, NCULAE-20221031103). Twenty-five female C57BL/6 mice (8 weeks old) were randomly divided into 5 groups (5 mice per group) as follows: (a) Sham group, (b) ovariectomy-induced OP group (OVX group), (c) A-Z@Pd(H) treatment group [OVX + A-Z@Pd(H) group], (d) A-Z treatment group (OVX + A-Z group), and (e) A-Z@Pd treatment group (OVX + A-Z@Pd group). The murine ovariectomy-induced OP model was established as previously described. Mice with preserved ovaries and removed fat were established as the Sham group. Eight weeks after surgery, the treatment groups received intravenous administration of 200 μl of various nanomaterials (1 mg/ml, twice a week for 4 weeks), while animals in the Sham and OVX groups were treated with an equivalent volume of normal saline solution. The body weight of mice in each group was monitored weekly throughout the experimental period. Four weeks after treatment, the mice were euthanized. The weight of the uterus was measured, and femur specimens were harvested for further analysis.

Skeletal analyses were performed using a micro-CT system (Scanco Medical, Zurich, Switzerland). The quantification parameters included BV/TV, BS/TV, Tb.N, SMI, and BMD. Following micro-CT analyses, fixed femur specimens were decalcified in a 10% ethylenediaminetetraacetic acid solution (Sigma-Aldrich) for 4 weeks, cut into 4-μm-thick serial sections, and subjected to histomorphological evaluation using H&E and Masson staining methods. TRAP staining was performed to quantify the number of TRAP-positive OCs in selected areas. Immunofluorescence staining was used to determine the number of CD206- and iNOS-positive cells within the selected area. For the evaluation of autophagy levels in bone tissue, immunofluorescence double staining experiments targeting LC3 and CD68 were conducted. All the above results were semiquantified using ImageJ software. Meanwhile, the bone tissues were collected for qRT-PCR analyses of OCN, CD206, iNOS, and TRAP genes.

To assess the in vivo biosafety of A-Z@Pd(H), major organs dissected from the OVX and OVX + A-Z@Pd(H) groups were subjected to H&E staining. Meanwhile, blood serum samples were collected from the mice for analyses of blood biochemical indexes including ALT, AST, BUN, and CREA. Additionally, major organs from mice in the OVX group and the OVX + A-Z@Pd(H) groups, including hearts, livers, spleens, lungs, kidneys, and bones, were excised. The Pd content was analyzed by ICP-MS to determine its concentration in the tissues.

### Statistical analyses

All experiments were performed at least 3 times. All data were presented as means ± SD. Statistical analyses among groups was performed using one-way analyses of variance (ANOVAs) and *t* test with GraphPad Prism version 9.4.1. Statistical values of *P < 0.05, **P < 0.01, ***P < 0.001, ****P < 0.0001 were regarded statistically significant.

## Data Availability

The data that support the findings of this study are available from the corresponding author upon reasonable request.
