# Supplementary material for: Palladium-Based Nanocomposites Remodel Osteoporotic Microenvironment by Bone-Targeted Hydrogen Enrichment and Zincum Repletion
Source: Research (Wash D C). 2024 Dec 17;7:0540. doi: 10.34133/research.0540 (PMC11651528; doi:10.34133/research.0540)
Supplement: Supplementary 1 — Figs. S1 to S30 Tables S1 and S2 [file research.0540.f1.zip › Supplemental Material-Marked.docx]

**Supporting Information**

**Palladium-Based Nanocomposites Remodel Osteoporotic Microenvironment by Bone-Targeted Hydrogen Enrichment and Zincum Repletion**

Lubing Liu^1,2,3^, Huiying Liu^1,2^, Xiaoya Lu^3^, Zhengshuai Yin^3^, Wei Zhang^3^, Jing Ye^1,2,3^, Yingying Xu^3^, Zhenzhen Weng^3^, Jun Luo^1,2^* and Xiaolei Wang^3^*

^1^The Department of Rehabilitation Medicine, the 2^nd^ Affiliated Hospital, Jiangxi Medical College, Nanchang University, Nanchang 330006, China. ^2^The Institute of Translational Medicine, the 2^nd^ Affiliated Hospital, Jiangxi Medical College, Nanchang University, Nanchang 330006, China. ^3^The National Engineering Research Center for Bioengineering Drugs and the Technologies, Institute of Translational Medicine, Nanchang University, Nanchang 330088, China.

*Address correspondence to: wangxiaolei@ncu.edu.cn (X. Wang); [luojun1786@163.com](mailto:luojun1786@163.com) (J. Luo)

**Keywords:** hydrogen therapy, ZIF-8, osteoporosis, osteoimmunomodulation, autophagy


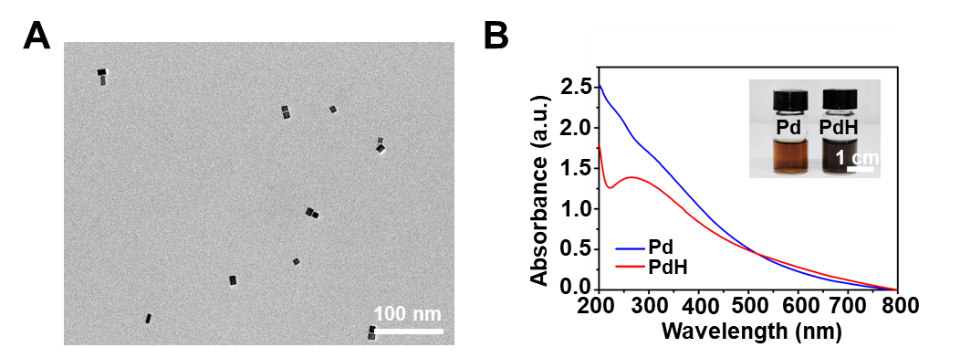


**Figure S1.** A) TEM image of Pd NCs. B) UV-vis spectra of Pd NCs and PdH NCs. Inset: optical pictures of Pd NCs and PdH NCs solutions.


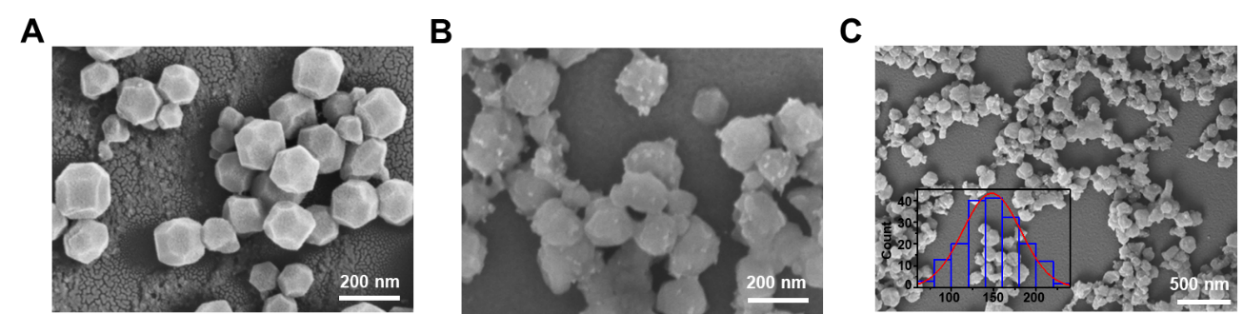


**Figure S2.** SEM images of A) ZIF-8 and B-C) A-Z@Pd. Inset: a statistical map of particle size distribution.


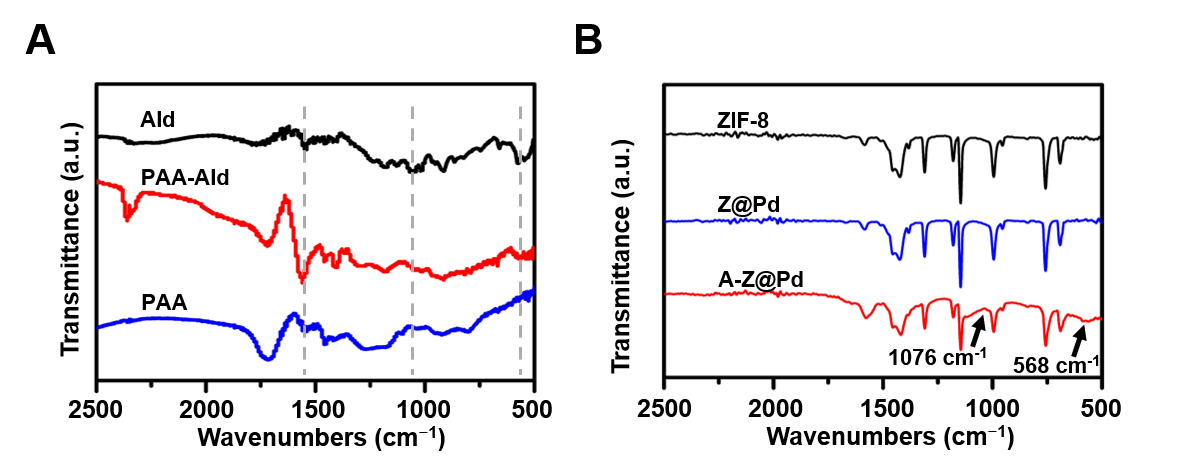


**Figure S3.** A) FTIR transmission spectra of Ald, PAA-Ald and PAA. B) FTIR transmission spectra of ZIF-8, Z@Pd and A-Z@Pd.


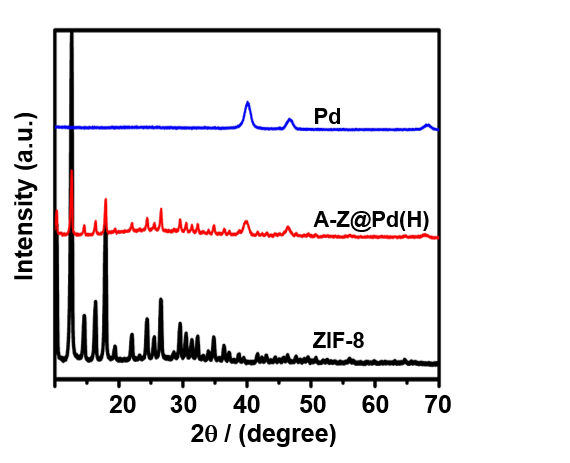


**Figure S4.** XRD patterns of ZIF-8, Pd and A-Z@Pd.


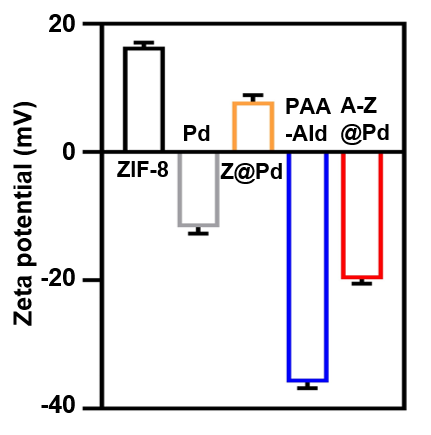


**Figure S5.** Zeta potential of ZIF-8, Pd, Z@Pd, PAA-Ald and A-Z@Pd.


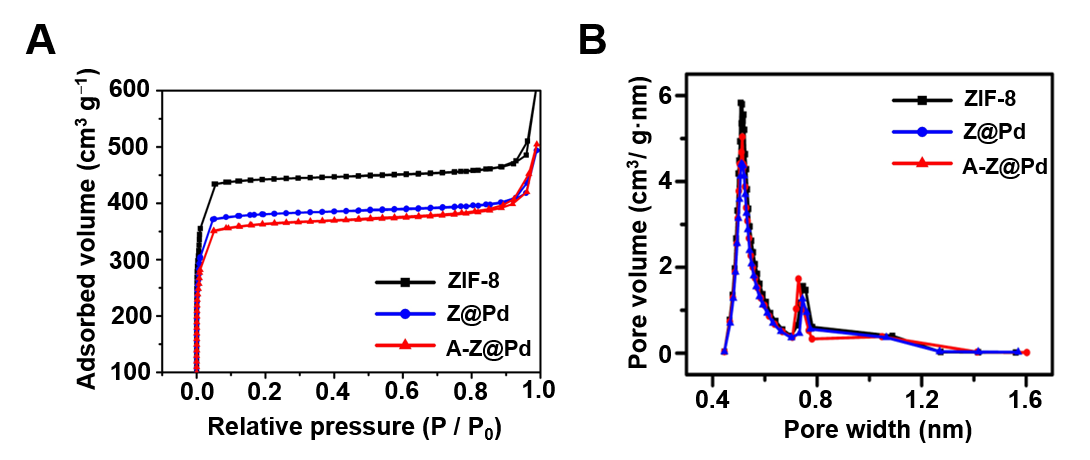


**Figure S6.** A) Nitrogen adsorption-desorption curves of ZIF-8, Z@Pd and A-Z@Pd. B) Pore-size distributions of ZIF-8, Z@Pd and A-Z@Pd.


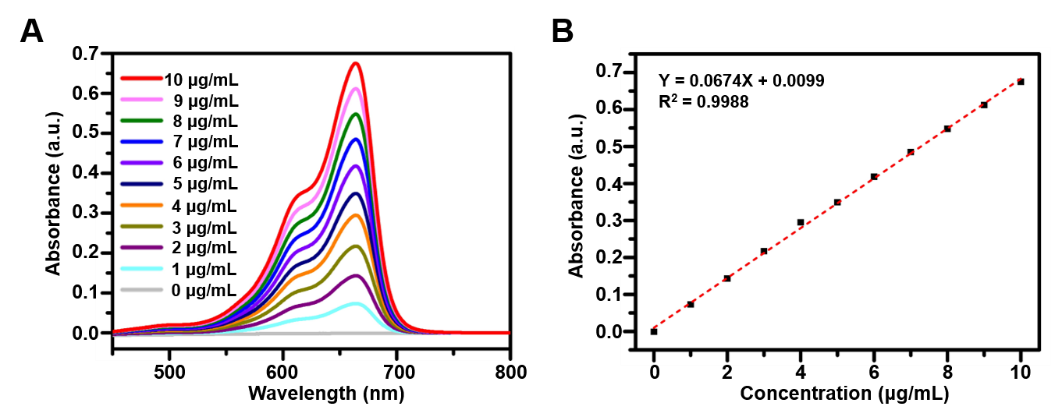


**Figure S7.** A) UV-vis spectra of MB at different concentrations. B) The standard curve of MB linearly fitted between absorption intensity and MB concentration.


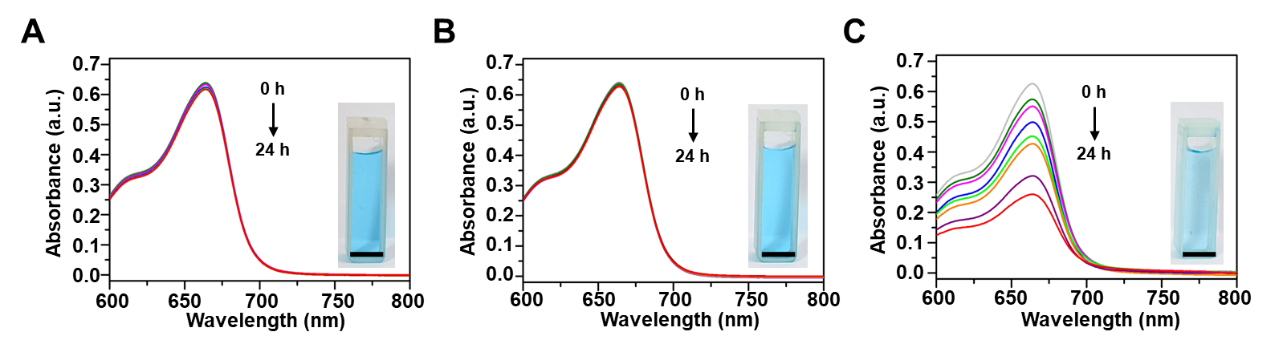


**Figure S8.** A-C) Time-dependent UV-vis-NIR spectra of MB solution after addition of (A) HRW, (B) A-Z@Pd and (C) A-Z@Pd(H). Inset: optical images of MB solutions. (Scale bar = 1 cm).


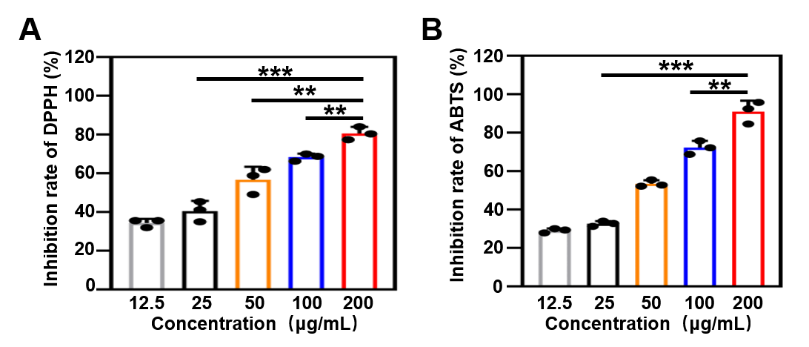


**Figure S9.** A) DPPH and B) ABTS radicals scavenging ability of A-Z@Pd(H) with different concentrations. Data are means ± s.d. (n ≥ 3). ***p* < 0.01, ****p* < 0.001.


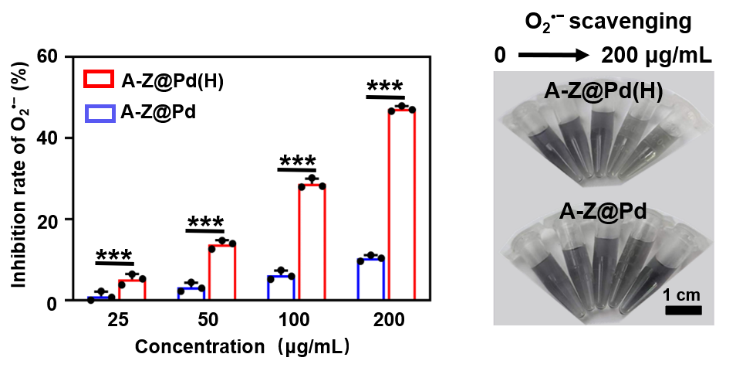


**Figure S10.** The O_2_^•−^ inhibition rate of A-Z@Pd and A-Z@Pd(H) with different concentrations. Inset: corresponding images of the reaction. Data are means ± s.d. (n ≥ 3). ****p* < 0.001.


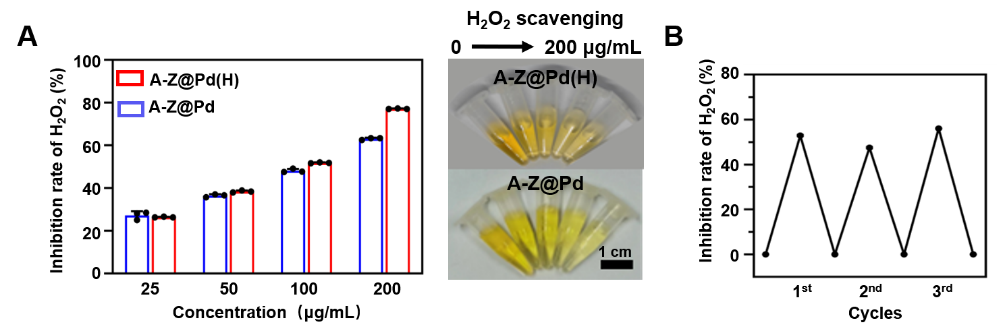


**Figure S11.** A) The H_2_O_2_ inhibition rate of A-Z@Pd and A-Z@Pd(H) with different concentrations. Inset: corresponding images of the reaction. B) Sustainability of the H_2_O_2_ scavenging activities of A-Z@Pd(H). Data are means ± s.d. (n ≥ 3). ****p* < 0.001.


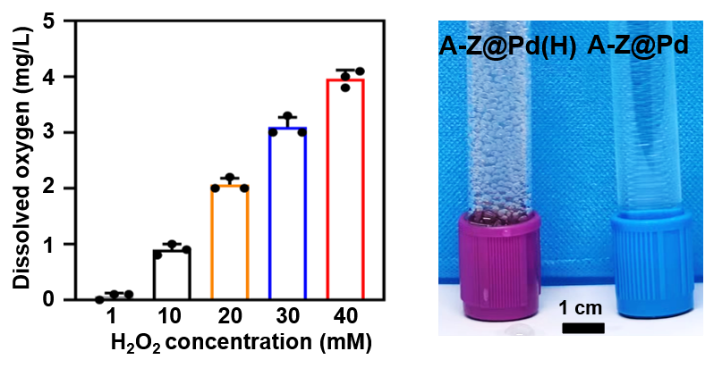


**Figure S12.** The content of dissolved oxygen catalyzed by A-Z@Pd(H) at different concentrations of H_2_O_2_. Inset: images of the bubbles produced in the reaction during H_2_O_2_ scavenging. Data are means ± s.d. (n ≥ 3).


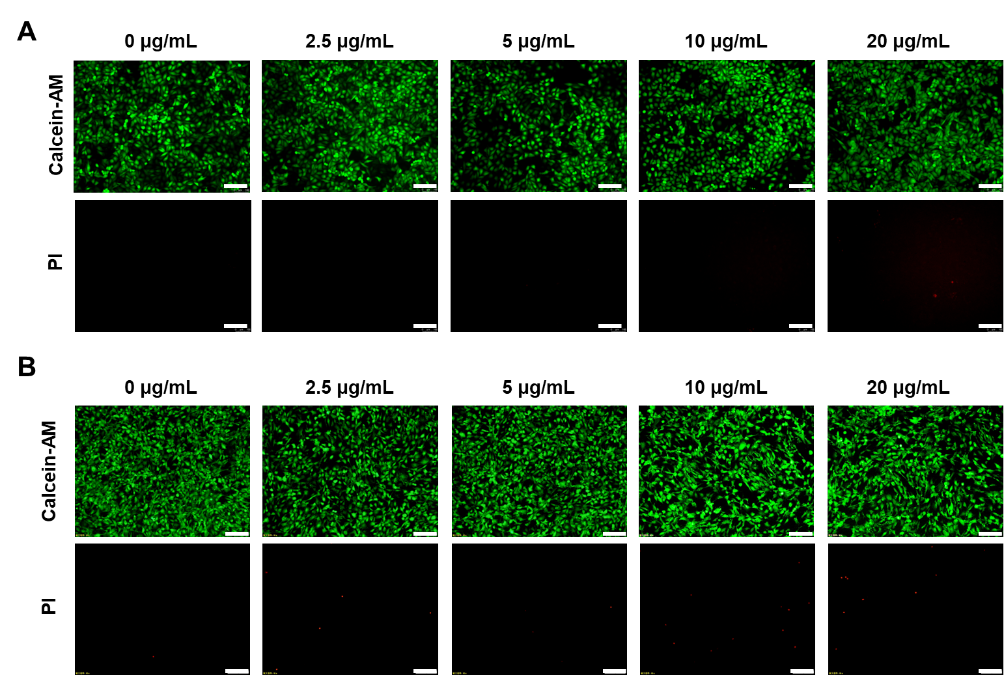


**Figure S13.** A-B) Live/dead staining of (A) HUVECs and (B) MC3T3-E1 cells after treatment with A-Z@Pd(H) of different concentrations. Green fluorescence (Calcein-AM) represented living cells, while red fluorescence (PI) represented dead cells. (Scale bar =200 μm).


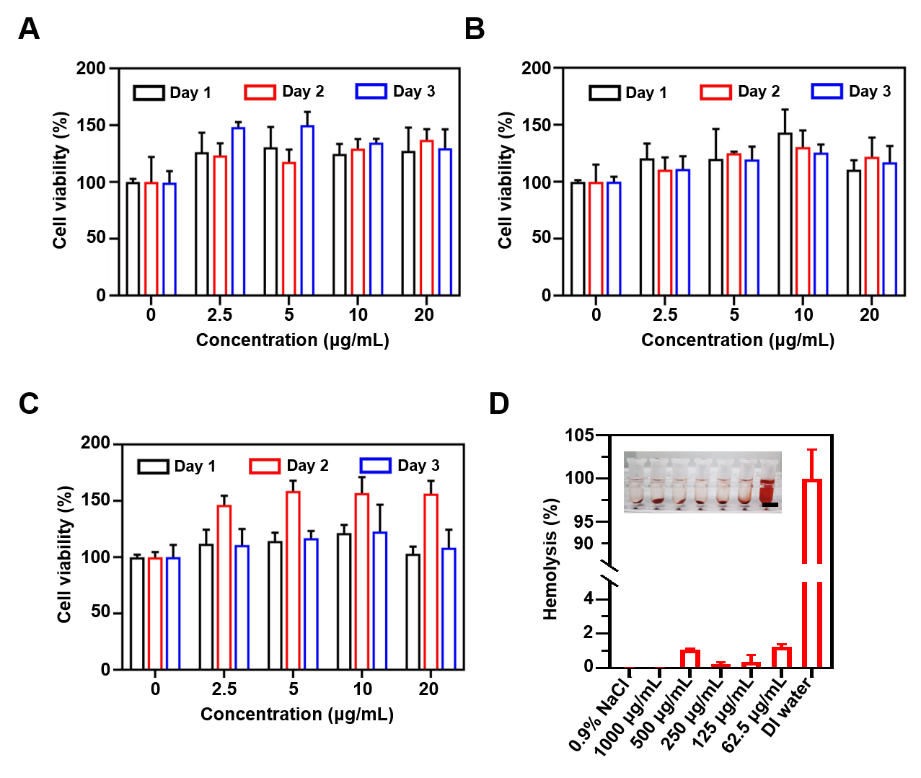


**Figure S14.** A-C) The cytotoxicity of different concentrations of A-Z@Pd(H) cultured with (A) HUVECs, (B) MC3T3-E1 cells and (C) RAW246.7 cells for 1, 2 and 3 days. D) Hemolysis analysis of red blood cells after co-culture with various treatments. Inset: an optical photograph of red blood cells. (Scale bar =1 cm). Data are means ± s.d. (n ≥ 3).


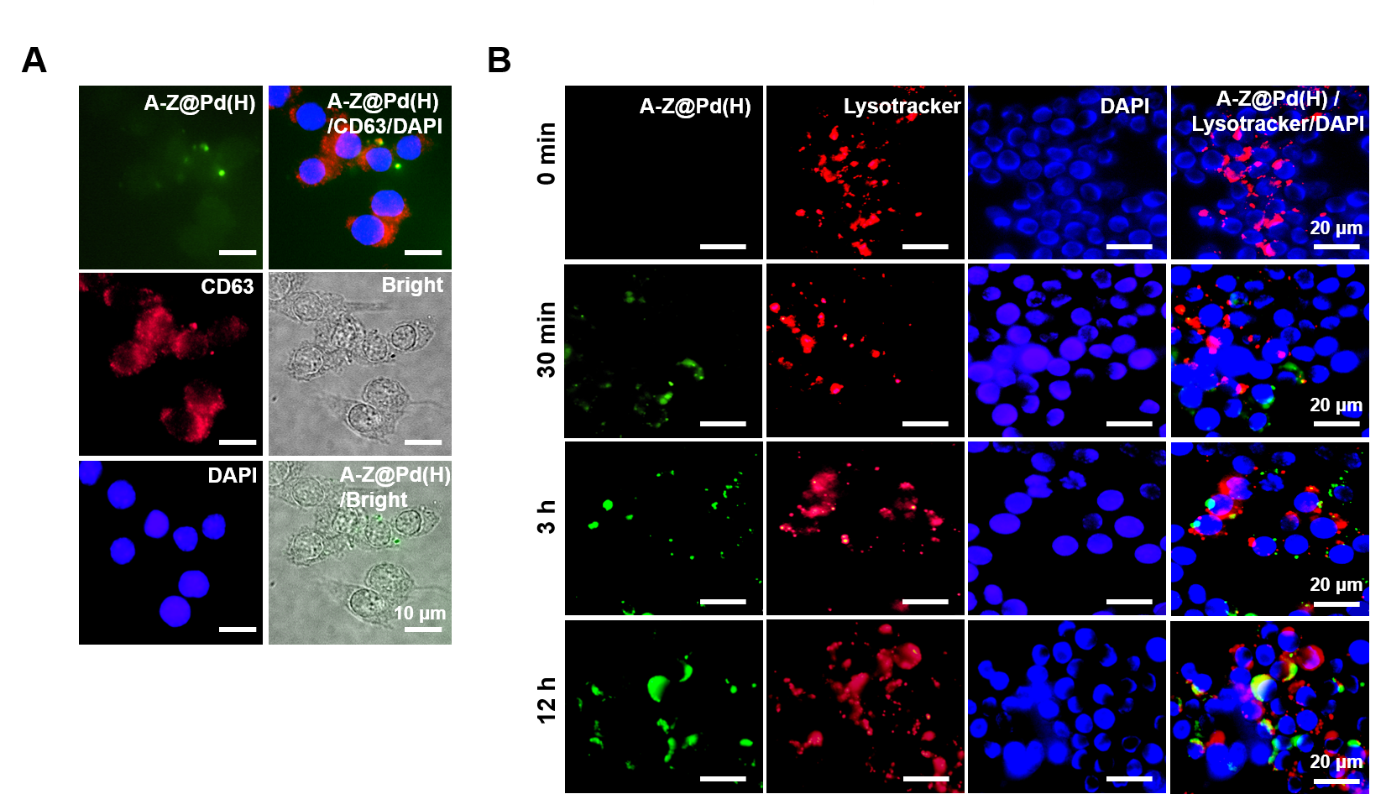


**Figure S15.** A) Live cell imaging system analyses showing intracellular trafficking of FITC-labeled A-Z@Pd(H) after incubation with RAW264.7 cells and the colocalization with CD63. B) Incubation with RAW264.7 cells for various periods and the colocalization with lysosomal markers (Lysotracker).


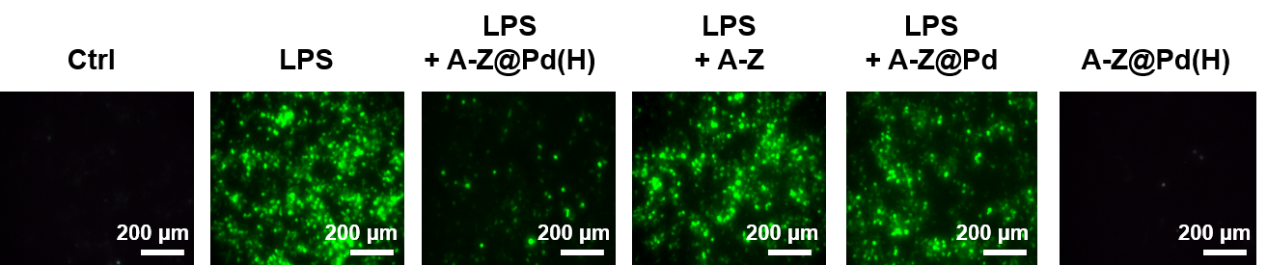


**Figure S16.** Fluorescence staining images of ROS in RAW264.7 cells after different treatments.


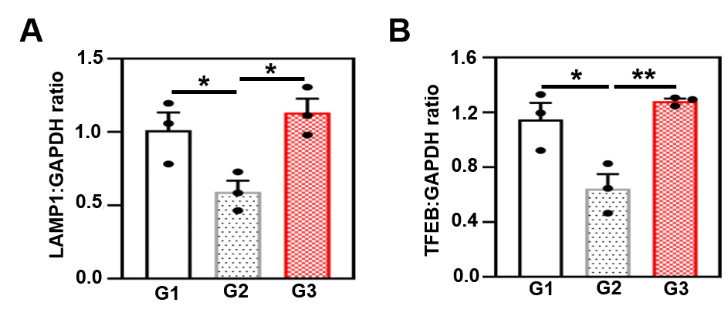


**Figure S17.** A-B) The relative intensity quantification of WB results of A) LAMP1 and B) TFEB proteins. G1: Ctrl; G2: LPS; G3: LPS + A-Z@Pd(H). Data are means ± s.d. (n ≥ 3). **p* < 0.05, ***p* < 0.01.


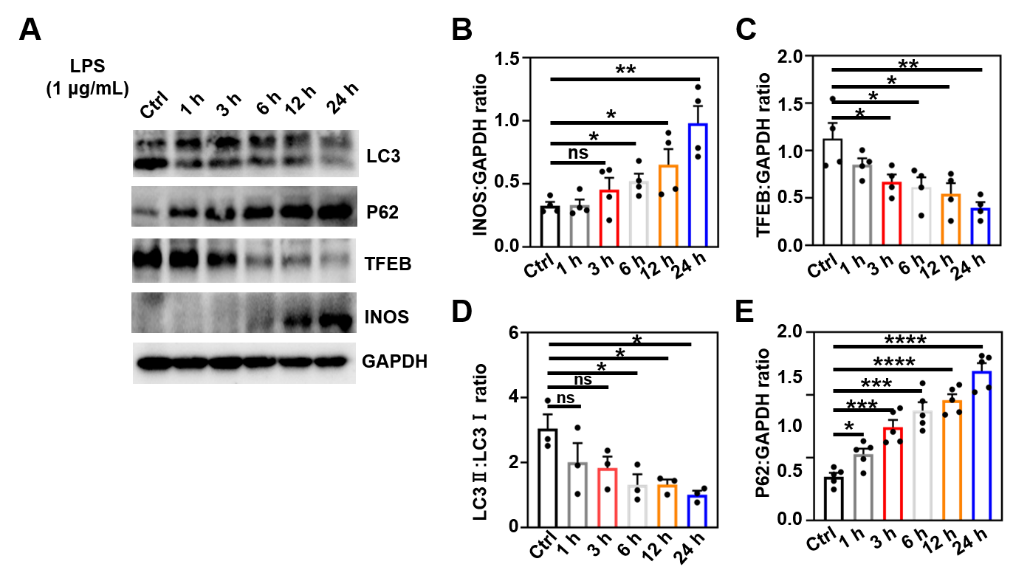


**Figure S18.** A) LC3, p62, TFEB and INOS transcription factor levels analyzed by WB at different times of LPS stimulation. B-E) Relative intensity quantification of different factors. Data are means ± s.d. (n ≥ 3). **p* < 0.05, ***p* < 0.01, ****p* < 0.001, *****p* < 0.0001. ns: no significance.


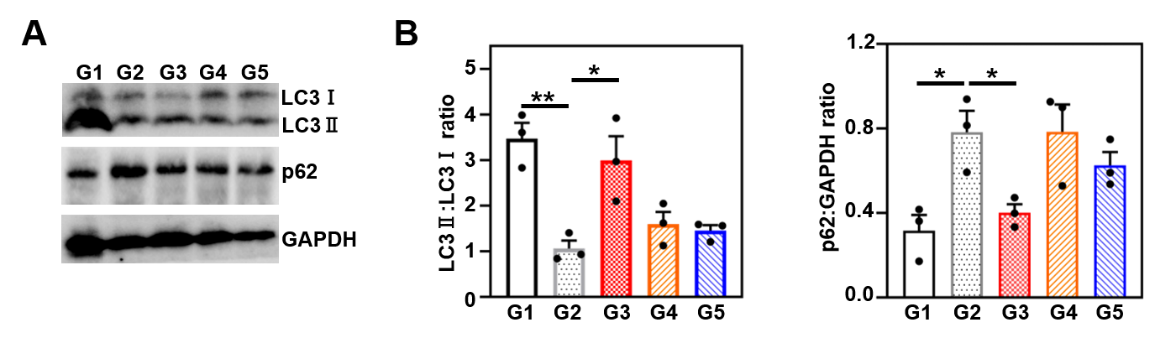


**Figure S19.** A) LC3 and p62 protein levels analyzed by WB in different groups and B) the relative intensity quantification. G1: Ctrl; G2: LPS; G3: LPS + A-Z@Pd(H); G4: LPS + A-Z; G5: LPS + A-Z@Pd. Data are means ± s.d. (n ≥ 3). **p* < 0.05.


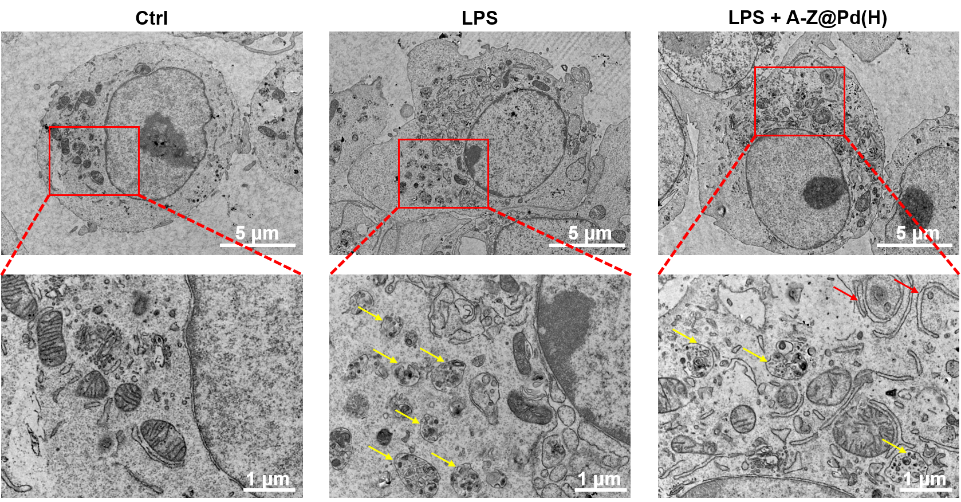


**Figure S20.** Representative electron microscopy images of RAW264.7 cells in different groups. The local high-magnification images are placed in the second line. Autophagolysosomes are indicated by yellow arrows, while autophagosomes are denoted by red arrows.


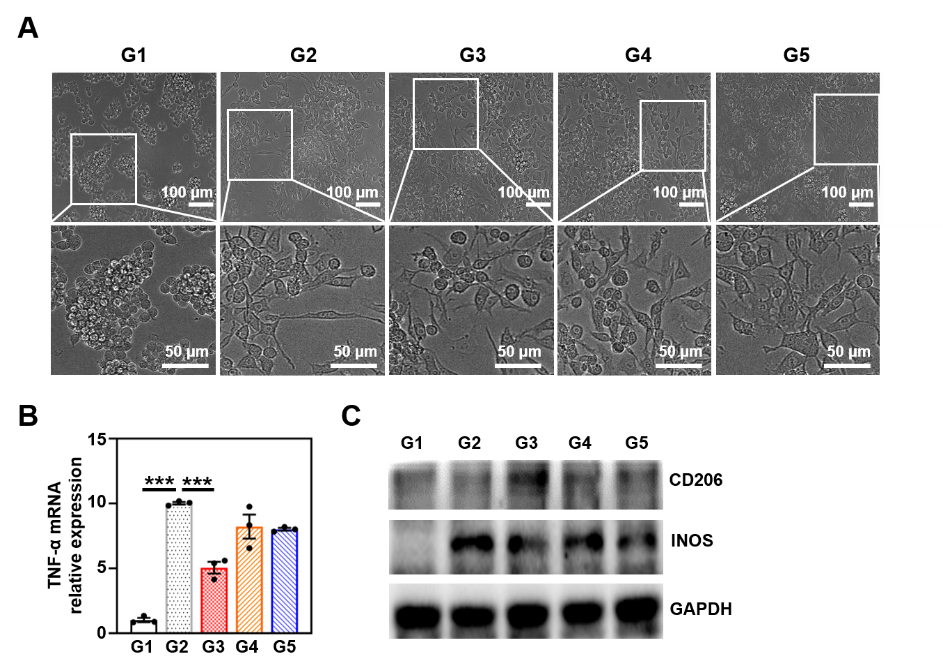


**Figure S21.** A) Light microscopy images showing macrophage morphology in different groups. B) qRT-PCR analyses of TNF-α mRNA levels in RAW264.7 cells after different treatments. C) WB analyses of CD206 and INOS protein levels in RAW264.7 cells after different treatments. G1: Ctrl; G2: LPS; G3: LPS + A-Z@Pd(H); G4: LPS + A-Z; G5: LPS + A-Z@Pd. Data are means ± s.d. (n ≥ 3). ****p* < 0.001.


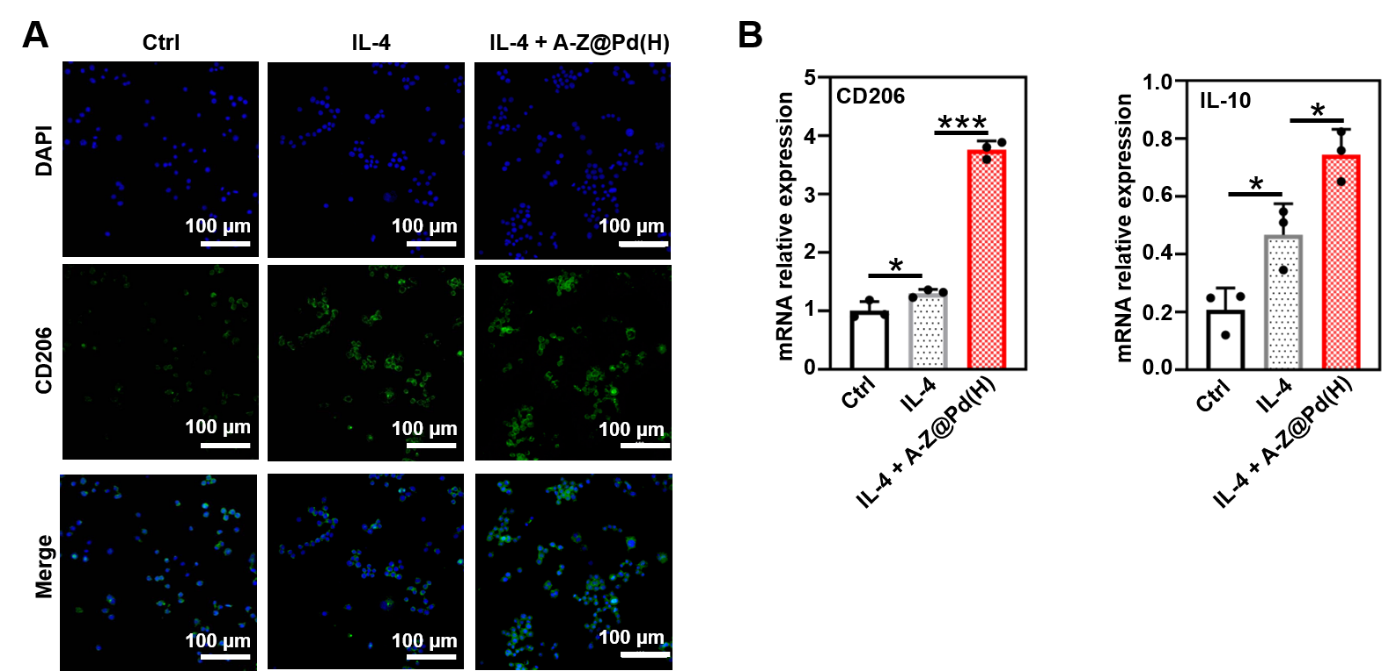


**Figure S22.** A) Representative immunofluorescence staining images of CD206 (green) in different groups. B) qRT-PCR analyses of CD206 and IL-10 expression in RAW264.7 cells among various groups. Data are means ± s.d. (n ≥ 3). **p* < 0.05, ***p* < 0.01, ****p* < 0.001.


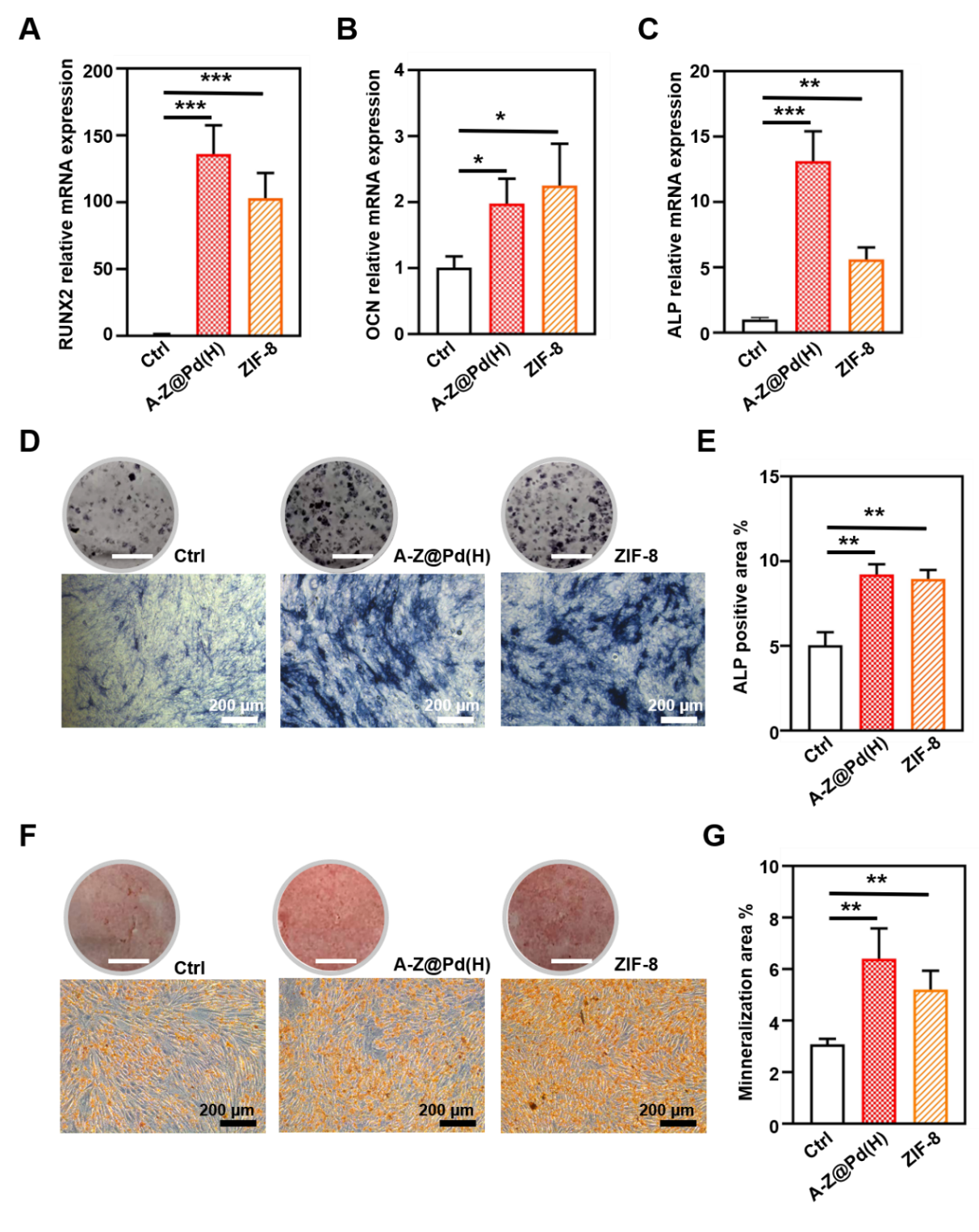


**Figure S23.** A-C) qRT-PCR analysis of (A) RUNX2, (B) OCN and (C) ALP after treatment of ZIF-8 and A-Z@Pd(H), respectively. D) ALP staining of MC3T3-E1 cells in different groups and E) the quantitative analysis. F) Calcium deposition stained with ARS and G) the quantitative analysis. The circular picture on the upper side is an optical picture of the plate staining. (Scale bar = 1 cm). Data are means ± s.d. (n ≥ 3). **p* < 0.05, ***p* < 0.01, ****p* < 0.001.


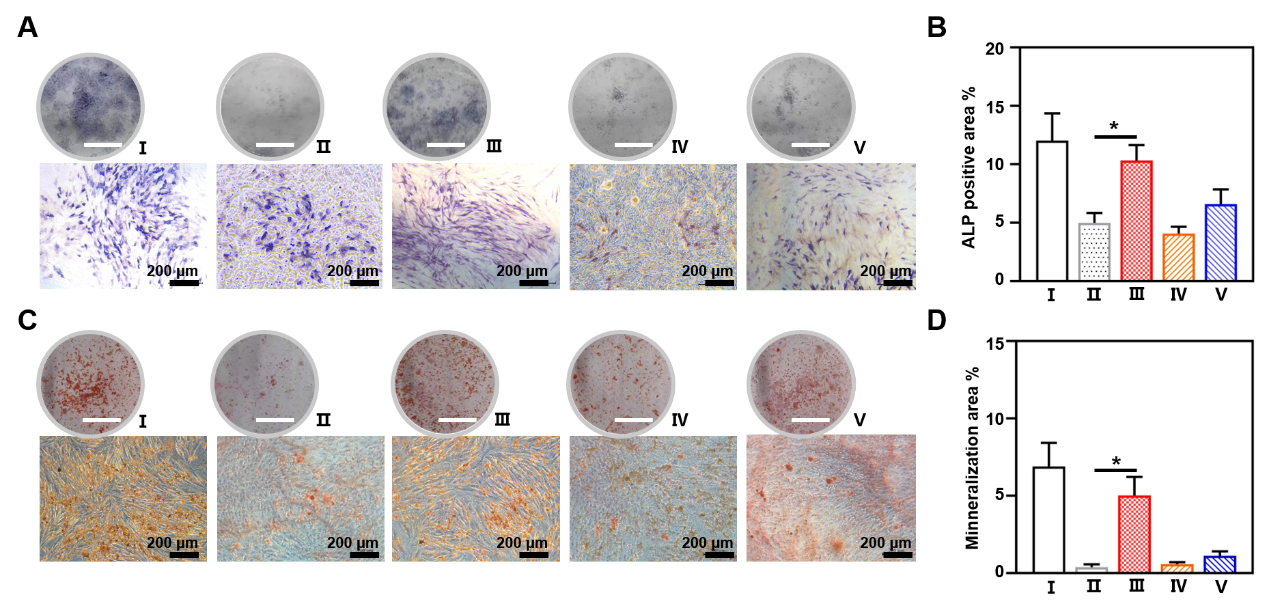


**Figure S24.** A-B) (A) ALP staining of MC3T3-E1 cells in different groups and (B) the quantitative analysis. C-D) (C) Calcium deposition stained with ARS and (D) the quantitative analysis. The circular picture on the upper side is an optical picture of the plate staining. (Scale bar = 1 cm). Ⅰ, Ⅱ, Ⅲ, Ⅳ and Ⅴ groups represent osteoblasts cultured with osteoblast inducing conditional media mixed with different macrophage culture supernatants collected from G1, G2, G3, G4 and G5 groups, respectively. G1: Ctrl; G2: LPS; G3: LPS + A-Z@Pd(H); G4: LPS + A-Z; G5: LPS + A-Z@Pd. Data are means ± s.d. (n ≥ 3). **p* < 0.05.


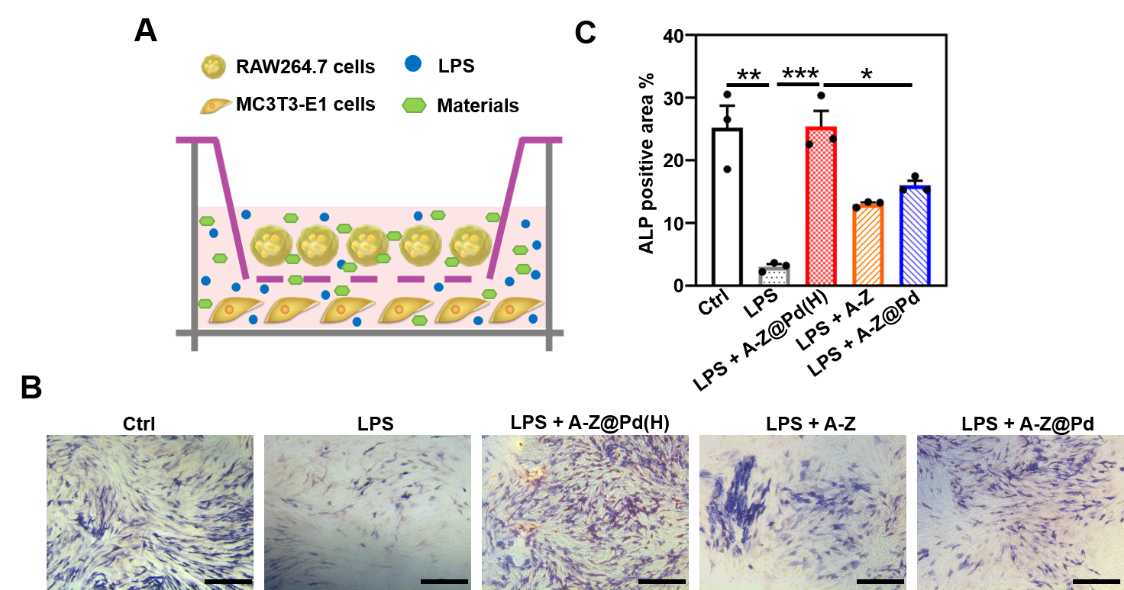


**Figure S25.** A) Schematic diagram of the Transwell co-culture experiment. B) Osteogenesis activity of MC3T3-E1 cells in the Ctrl, LPS, LPS + A-Z@Pd(H), LPS + A-Z, and LPS + A-Z@Pd groups characterized by ALP staining. (Scale bar = 500 μm). C) ALP positive area quantification. Data are means ± s.d. (n ≥ 3). **p* < 0.05, ***p* < 0.01, ****p* < 0.001.


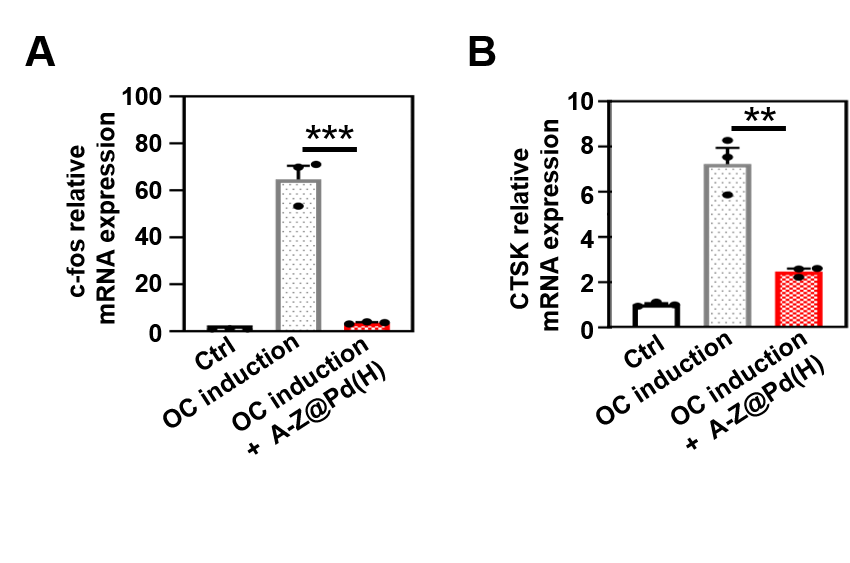


**Figure S26.** A-B) qRT-PCR analysis of different osteoclast-induced RAW264.7 cells for osteoclast-specific genes, including (A) c-fos and (B) CTSK. Data are means ± s.d. (n ≥ 3). ***p* < 0.01, ****p* < 0.001.


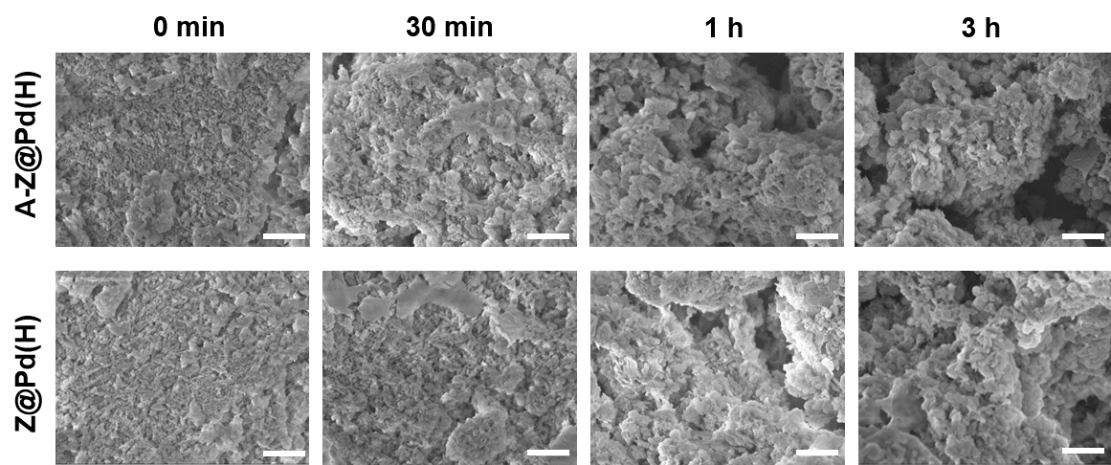


**Figure S27.** Original SEM images of A-Z@Pd(H) and Z@Pd(H) surface interactions with HAP after different time intervals. (Scale bar =1 μm).


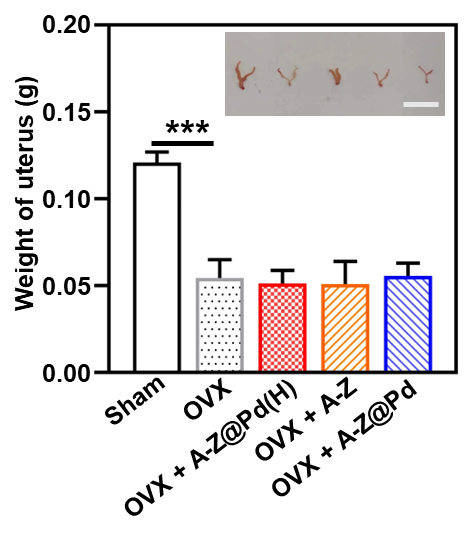


**Figure S28.** Uterine weight in each group of mice. Inset: optical pictures of uteri in different groups. (Scale bar = 1 cm). Data are means ± s.d. (n ≥ 3). ****p* < 0.001.


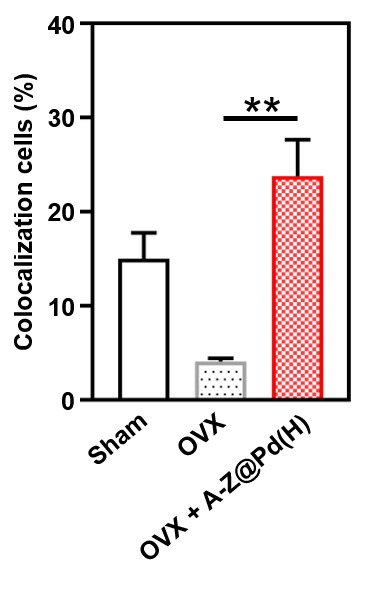


**Figure S29.** Quantification of cells showing colocalization of LC3 and CD68 fluorescence in femur sections. Data are means ± s.d. (n ≥ 3). ***p* < 0.01.


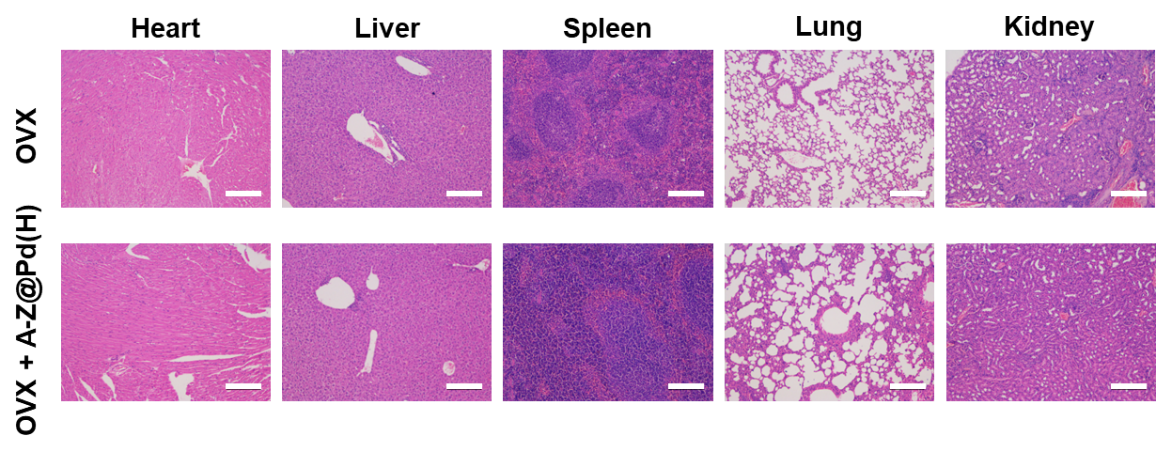


**Figure S30.** H&E staining images of vital organs in OVX and OVX + A-Z@Pd(H) groups at post-operative week 12. (Scale bar = 200 μm).

**TABLES**

**Table S1.** Quantification of Pd content (μg/g) in major organs by ICP-MS analyses.

**Table S2.** Quantitative-polymerase chain reaction primers used in this paper.

| Gene name | | | | Forward primer sequence | | | Reverse primer sequence |  |  |
| --- | --- | --- | --- | --- | --- | --- | --- | --- | --- |
| **IL-6** | 5’-GACAAAGCCAGAGTCCTTCAGA-3’ | | | | | 5’-TGTGACTCCAGCTTATCTCTTGG-3’ | |  |  |
| **IL-10** | | | 5’-TAACTGCACCCACTTCCCAG-3’ | | | 5’-TAGACACCTTGGTCTTGGAGCTTAT-3’ | | | |
| **TNF-α** | | | | | 5’-TATGGCTCAGGGTCCAACTC-3’ | | 5’-GGAAAGCCCATTTGAGTCCT-3’ |  |  |
| **RUNX2** | | 5’-GAACCAAGAAGGCACAGACAG-3’ | | | | 5’-CTGTAATCTGACTCTGTCCTTGTG-3’ | |  |  |
| **OCN** | | | 5’-AGCAGCTTGGCCCAGACCTA-3’ | | | 5’-TAGCGCCGGAGTCTGTTCACTAC-3’ | |  |  |
| **ALP** | | | | | 5’-ATCTTTGGTCTGGCTCCCATG-3’ | | 5’-TTTCCCGTTCACCGTCCAC-3’ |  |  |
| **CTSK** | | 5’-GAAGAAGACTCACCAGAAGCAG-3’ | | | | | 5’-TCCAGGTTATGGGCAGAGATT-3’ |  |  |
| **NFATC1** | | | | 5’-GACCCGGAGTTCGACTTCG-3’ | | 5’-TGACACTAGGGGACACATAACTG-3’ | |  |  |
| **c‐fos** | | | | | 5’-CGGGTTTCAACGCCGACTA-3’ | | 5’-TTGGCACTAGAGACGGACAGA-3’ |  |  |
| **MMP9** | | | | 5’-CTGGACAGCCAGACACTAAAG-3’ | | | 5’-CTCGCGGCAAGTCTTCAGAG-3’ |  |  |
| **TRAP** | | | | 5’-CACTCCCACCCTGAGATTTGT-3’ | | | 5’-CATCGTCTGCACGGTTCTG-3’ |  |  |
| **GAPDH** | | 5’-AGGTCGGTGTGAACGGATTTG-3’ | | | | 5’-TGTAGACCATGTAGTTGAGGTCA-3’ | | |  |
