# Supplementary figures and images for: Palladium-Based Nanocomposites Remodel Osteoporotic Microenvironment by Bone-Targeted Hydrogen Enrichment and Zincum Repletion
Source: Research (Wash D C). 2024 Dec 17;7:0540. doi: 10.34133/research.0540 (PMC11651528; doi:10.34133/research.0540)

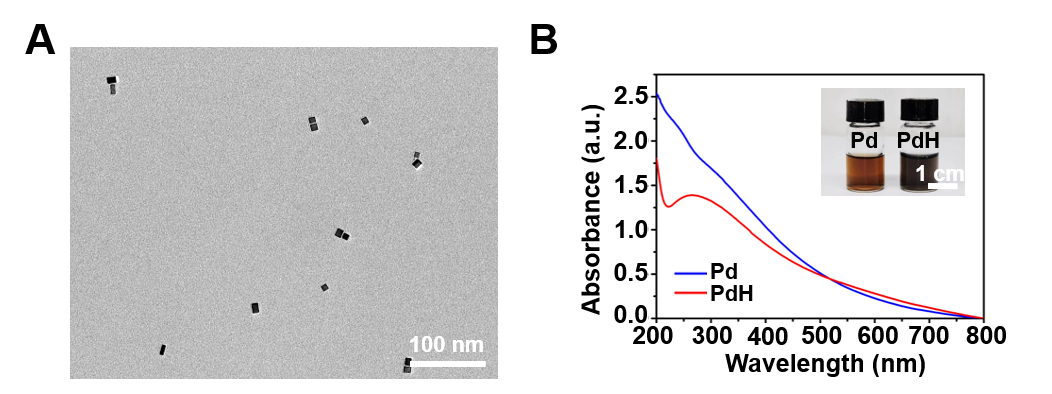

Supplement: Supplementary 1 — Figs. S1 to S30 Tables S1 and S2 [file research.0540.f1.zip › S1.tif]

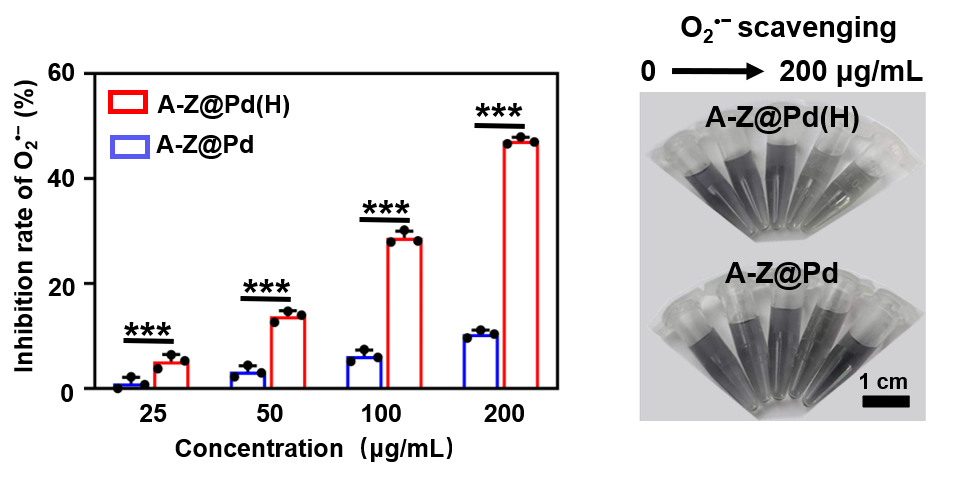

Supplement: Supplementary 1 — Figs. S1 to S30 Tables S1 and S2 [file research.0540.f1.zip › S10.tif]

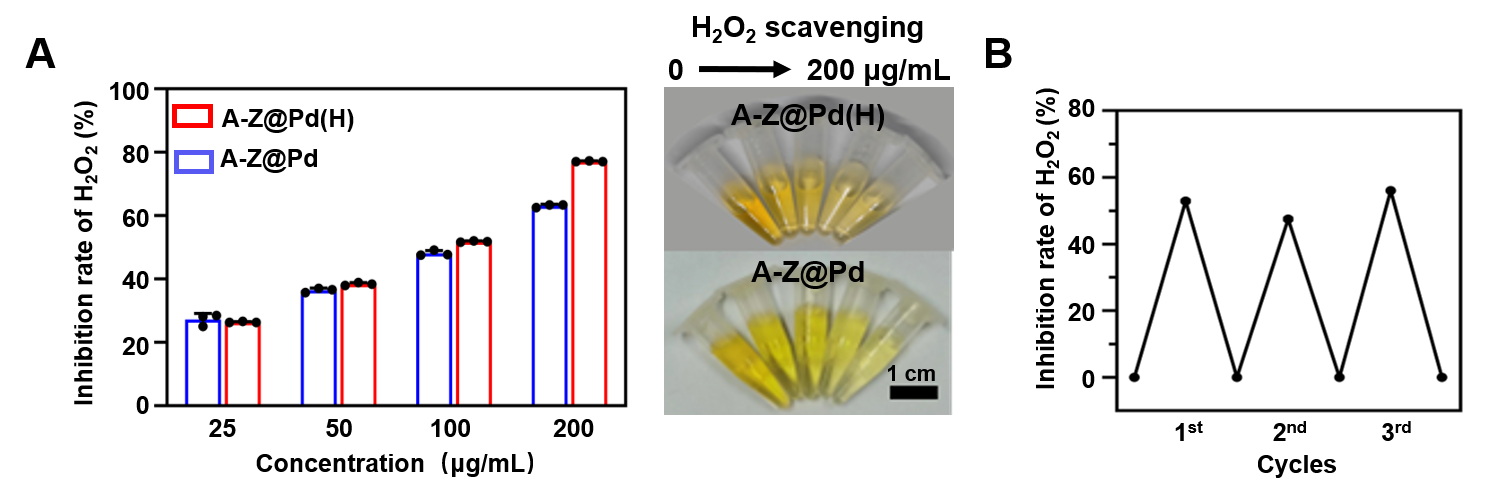

Supplement: Supplementary 1 — Figs. S1 to S30 Tables S1 and S2 [file research.0540.f1.zip › S11.tif]

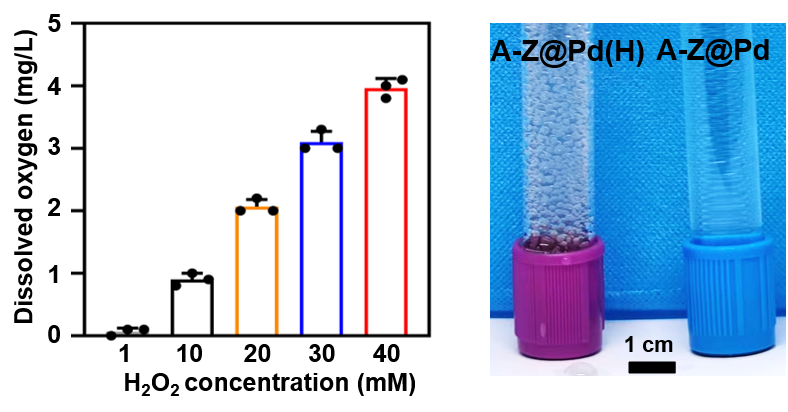

Supplement: Supplementary 1 — Figs. S1 to S30 Tables S1 and S2 [file research.0540.f1.zip › S12.tif]

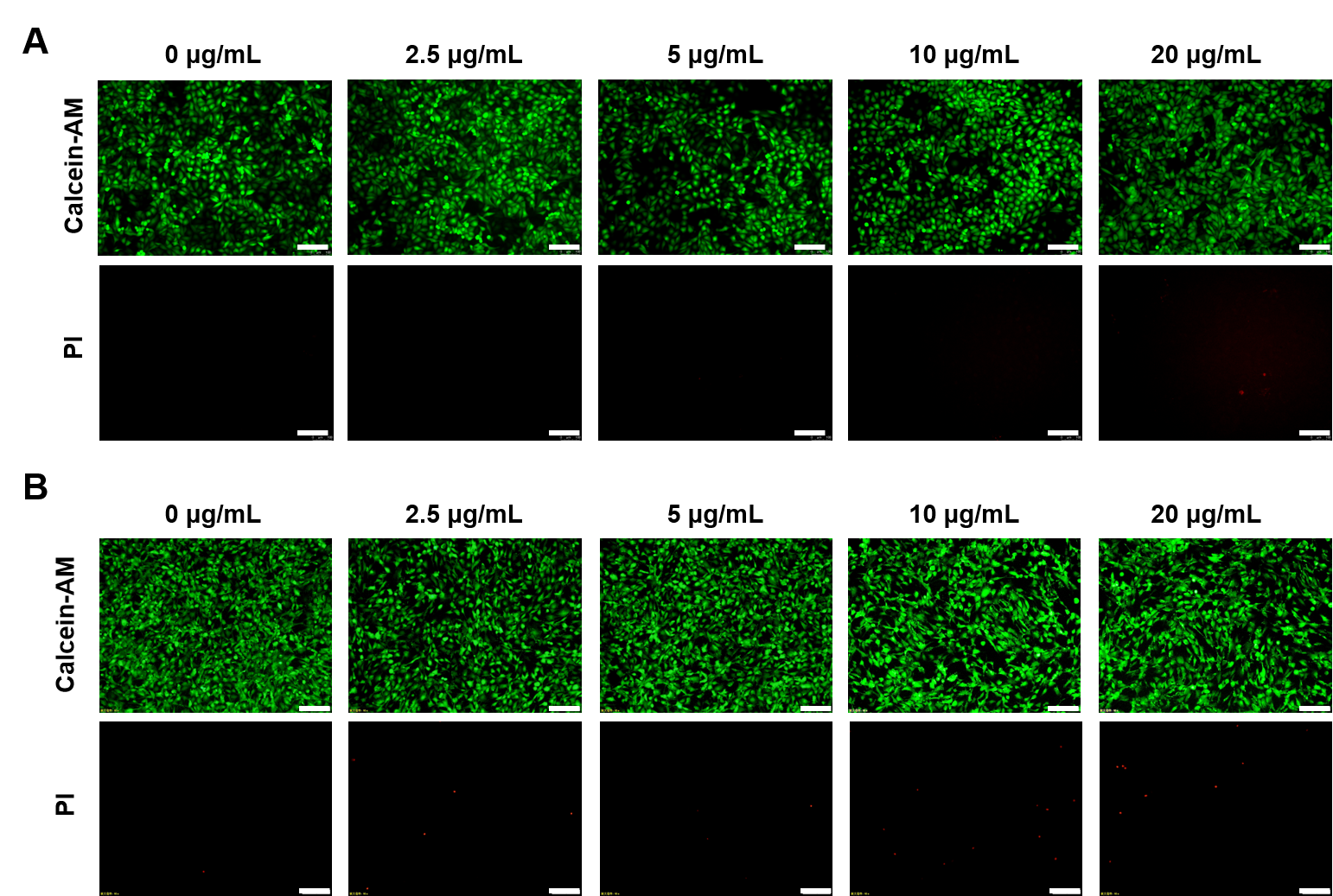

Supplement: Supplementary 1 — Figs. S1 to S30 Tables S1 and S2 [file research.0540.f1.zip › S13.tif]

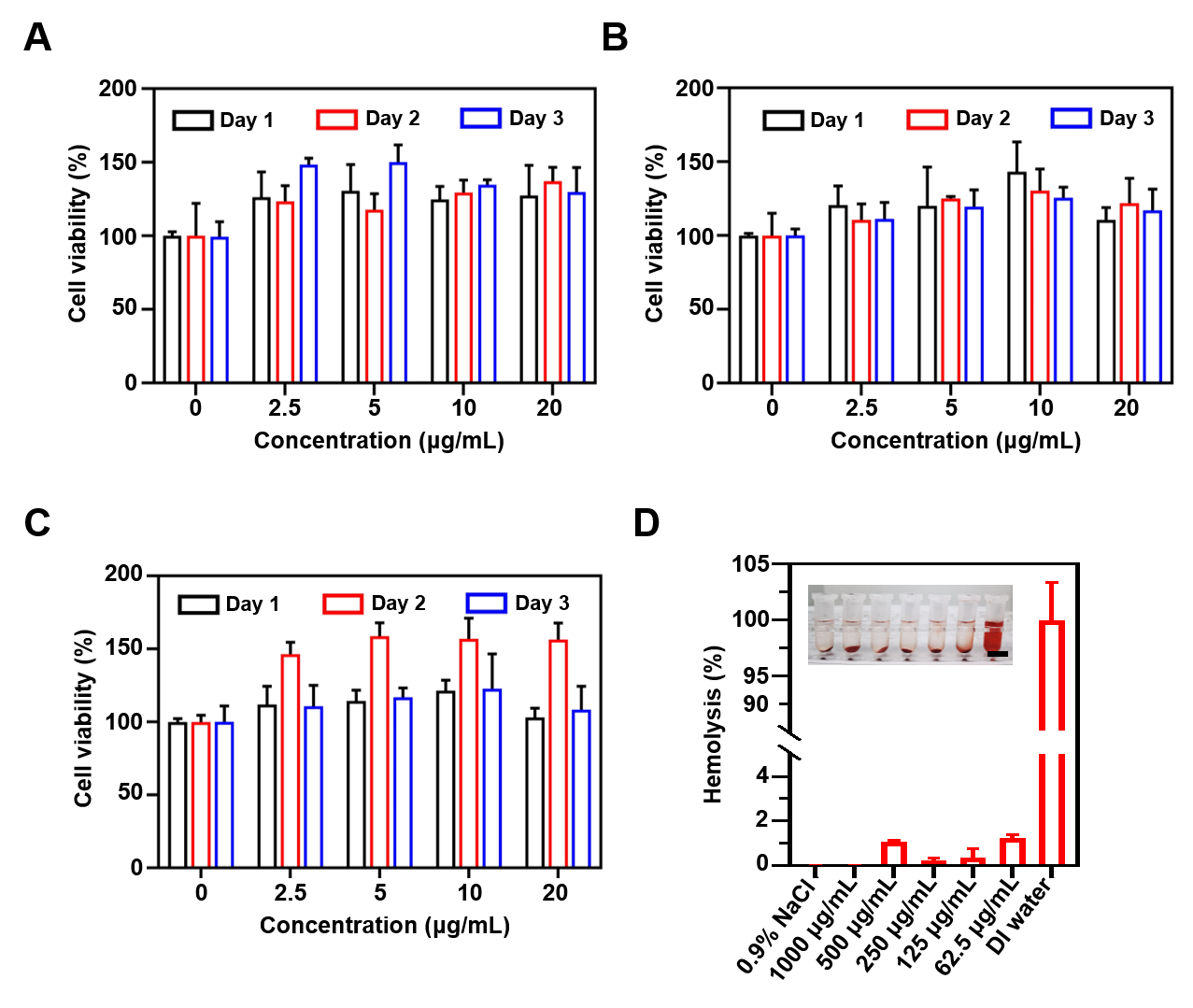

Supplement: Supplementary 1 — Figs. S1 to S30 Tables S1 and S2 [file research.0540.f1.zip › S14.tif]

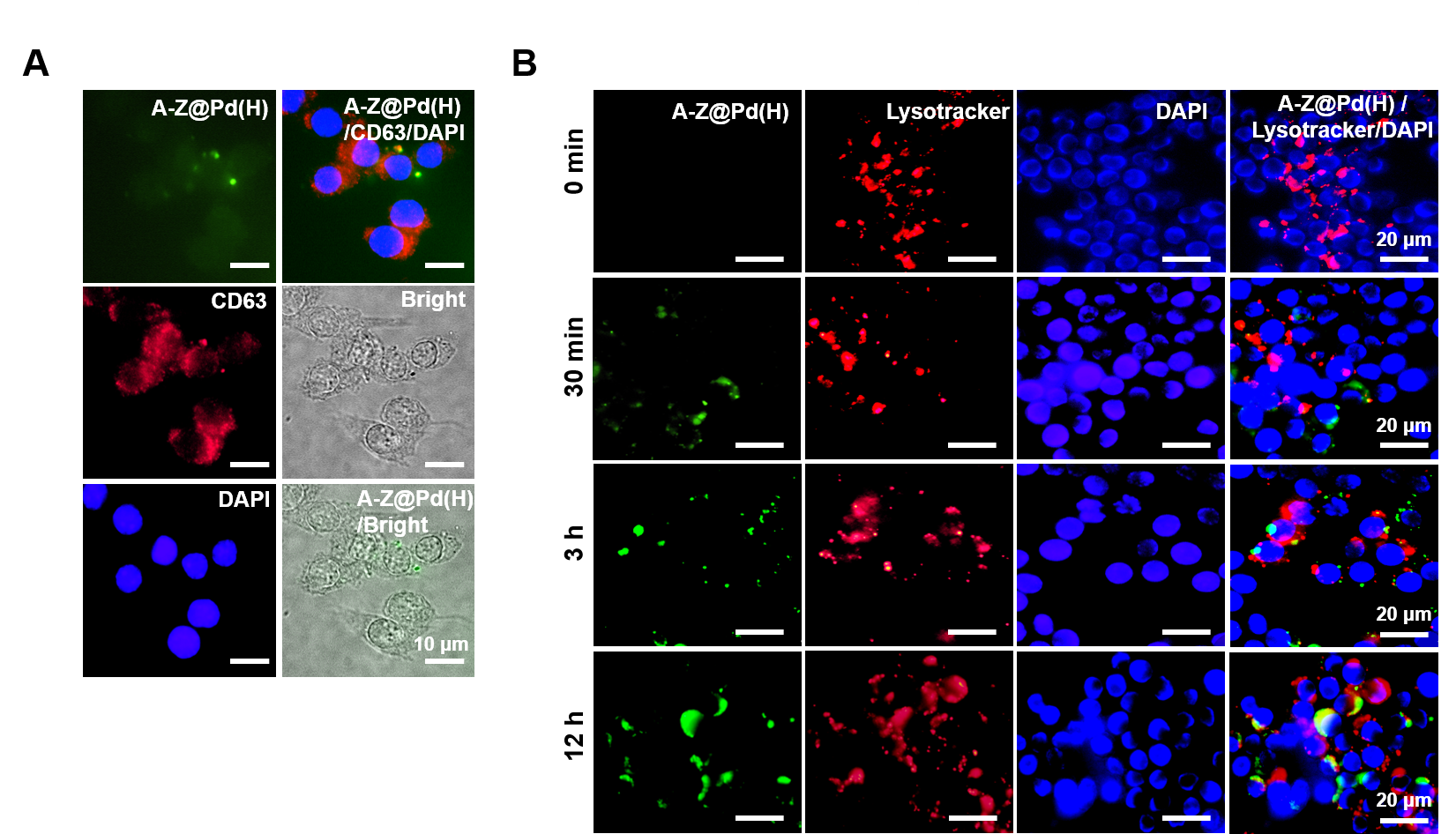

Supplement: Supplementary 1 — Figs. S1 to S30 Tables S1 and S2 [file research.0540.f1.zip › S15.tif]

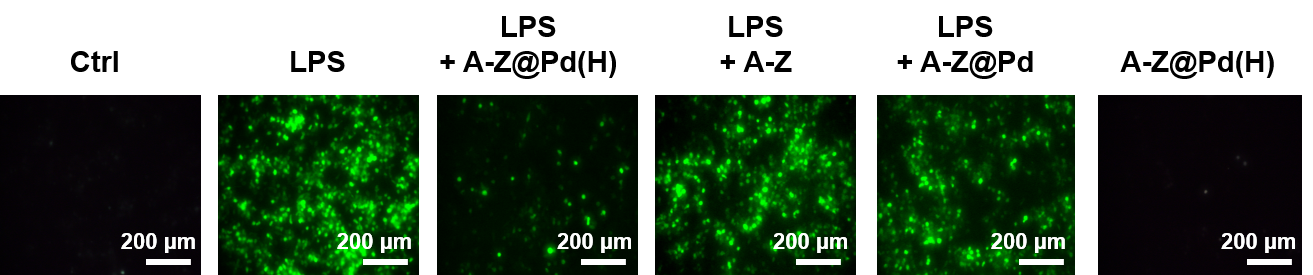

Supplement: Supplementary 1 — Figs. S1 to S30 Tables S1 and S2 [file research.0540.f1.zip › S16.tif]

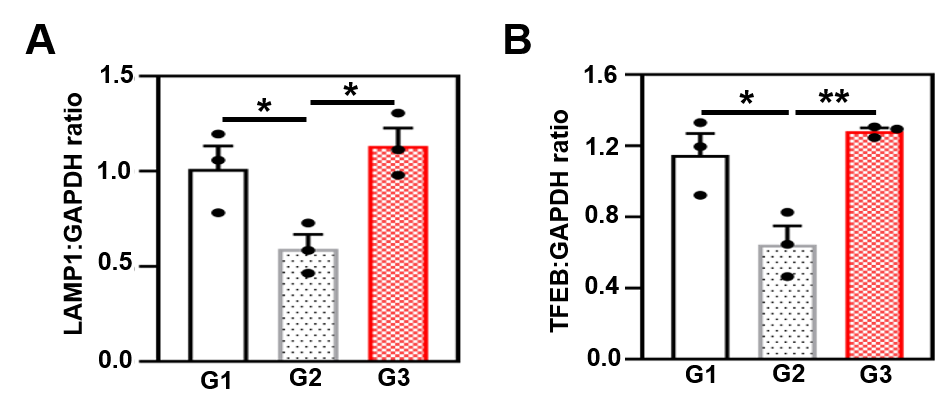

Supplement: Supplementary 1 — Figs. S1 to S30 Tables S1 and S2 [file research.0540.f1.zip › S17.tif]

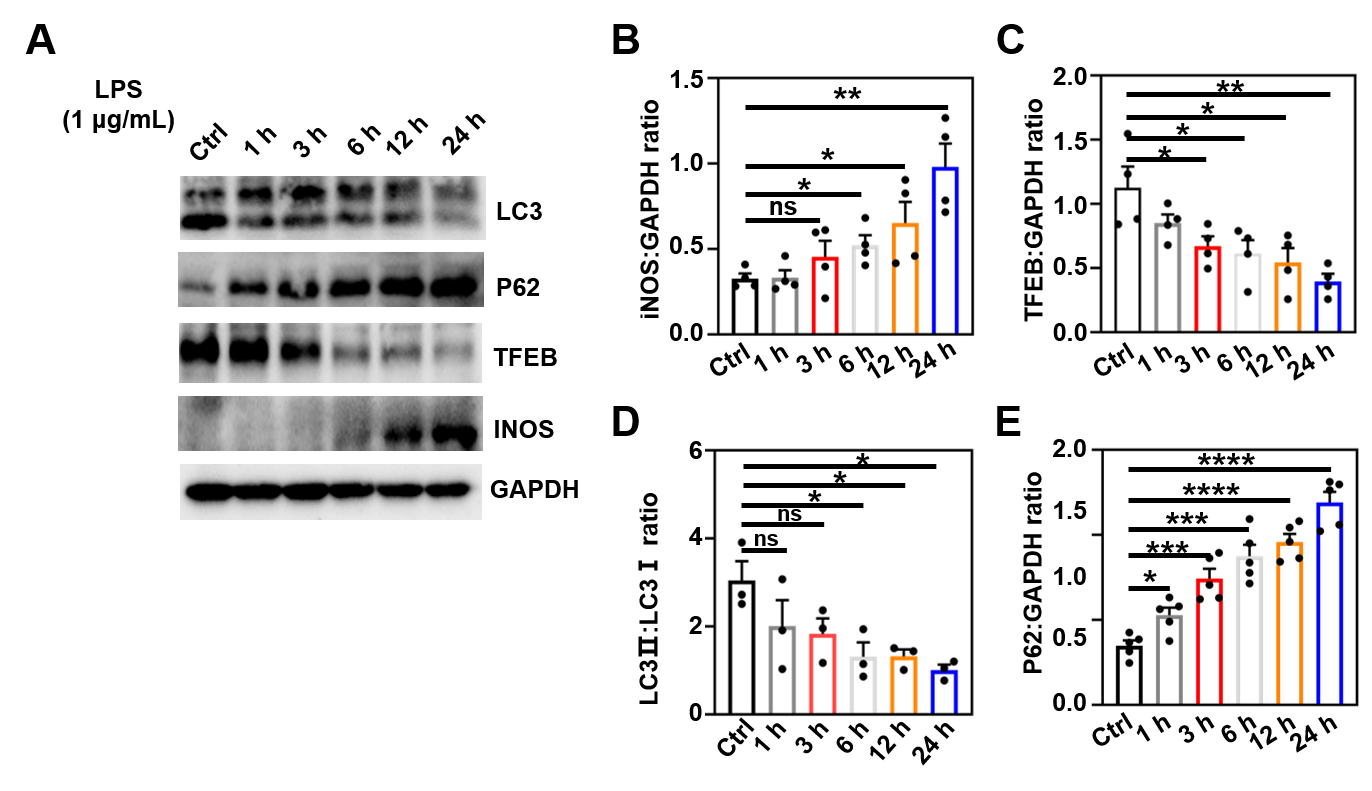

Supplement: Supplementary 1 — Figs. S1 to S30 Tables S1 and S2 [file research.0540.f1.zip › S18.tif]

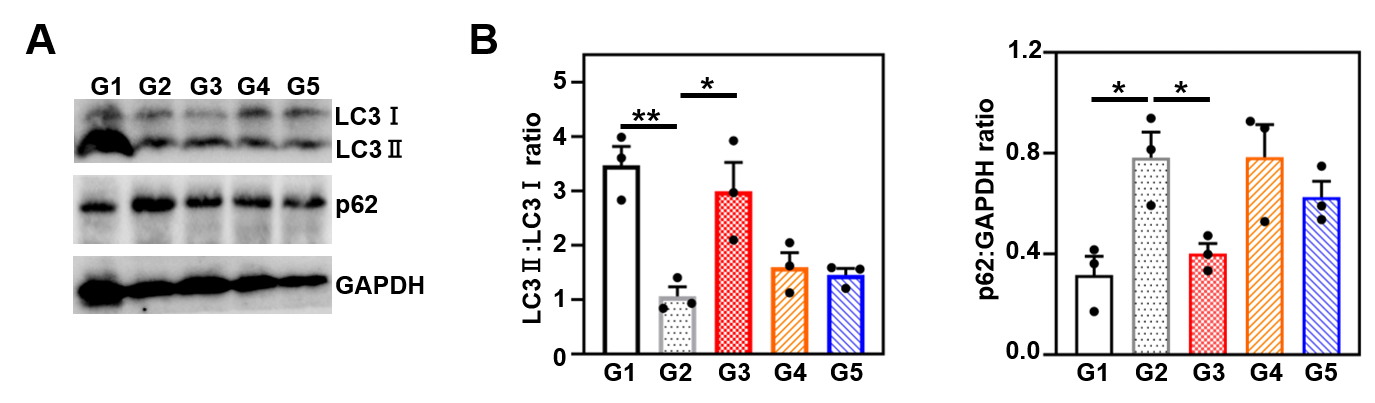

Supplement: Supplementary 1 — Figs. S1 to S30 Tables S1 and S2 [file research.0540.f1.zip › S19.tif]

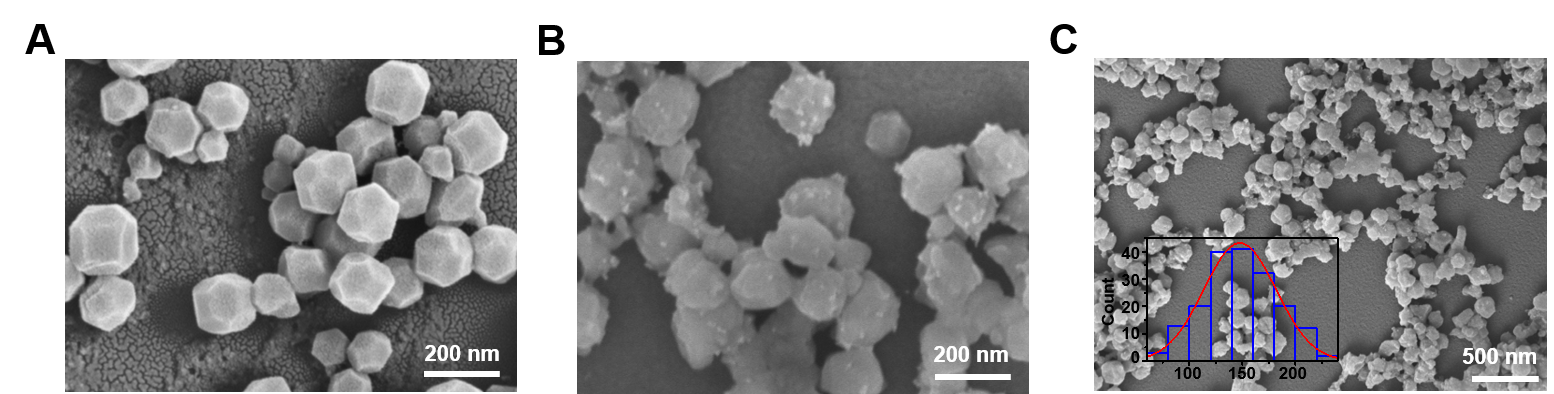

Supplement: Supplementary 1 — Figs. S1 to S30 Tables S1 and S2 [file research.0540.f1.zip › S2.tif]

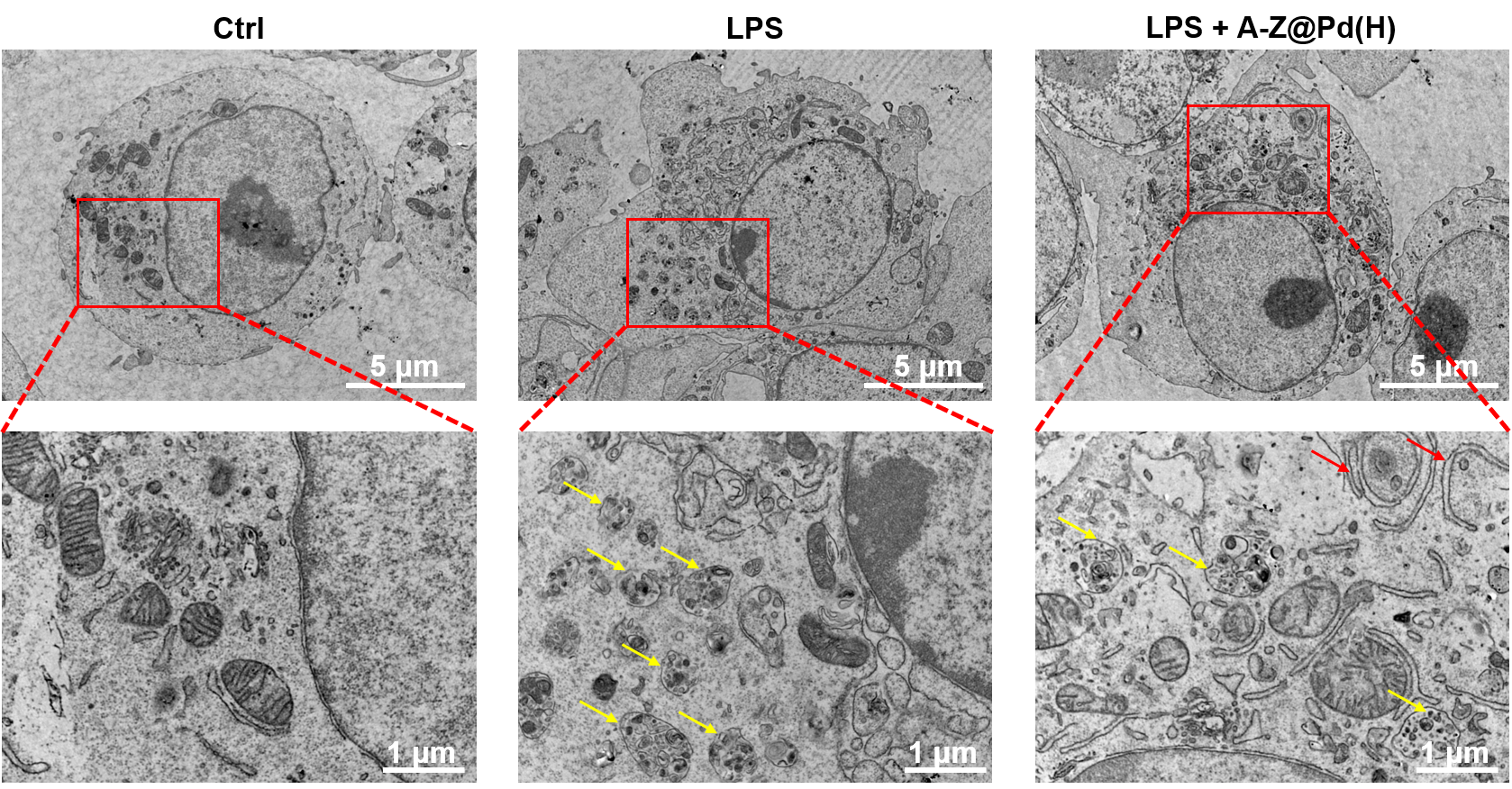

Supplement: Supplementary 1 — Figs. S1 to S30 Tables S1 and S2 [file research.0540.f1.zip › S20.tif]

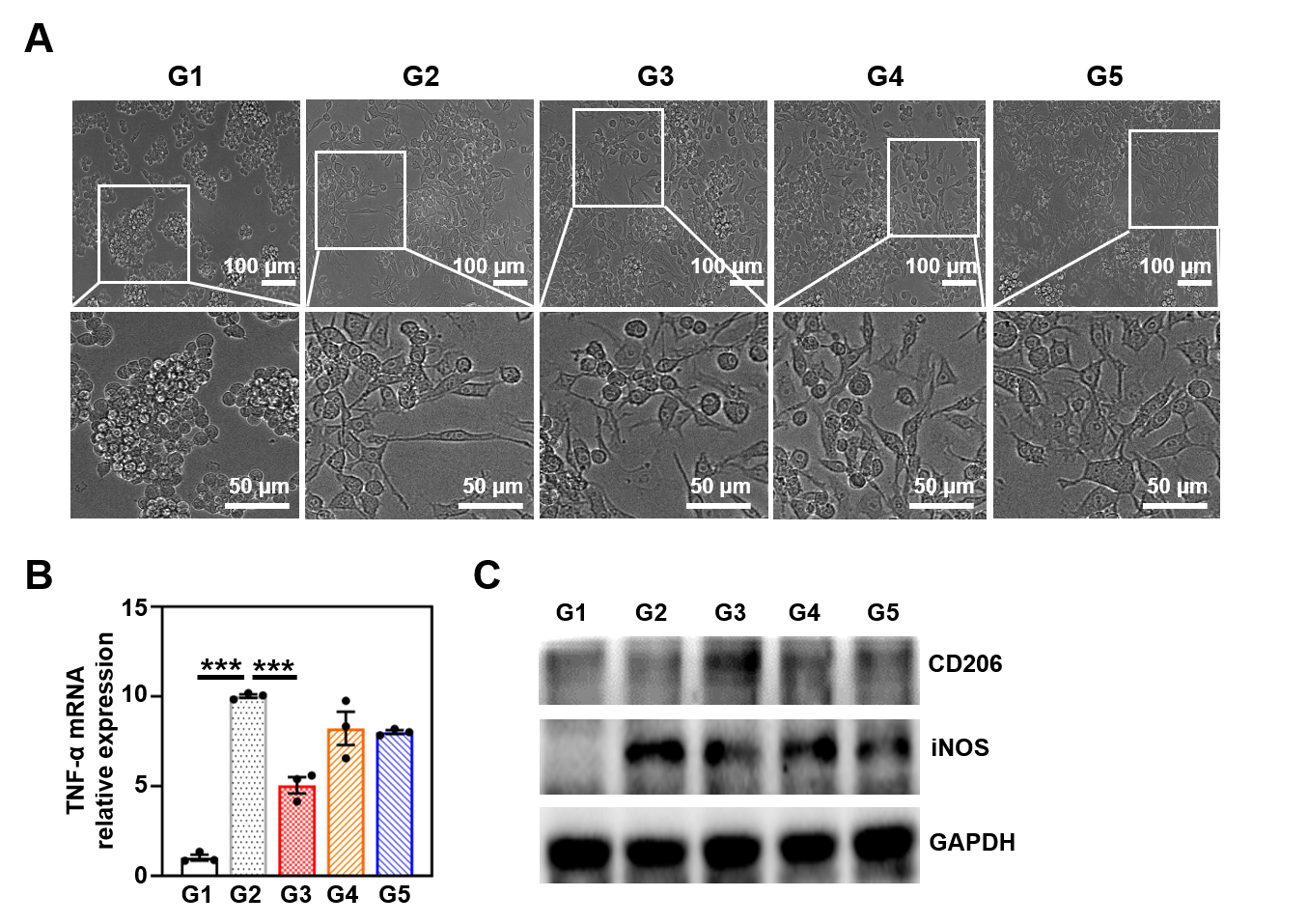

Supplement: Supplementary 1 — Figs. S1 to S30 Tables S1 and S2 [file research.0540.f1.zip › S21.tif]

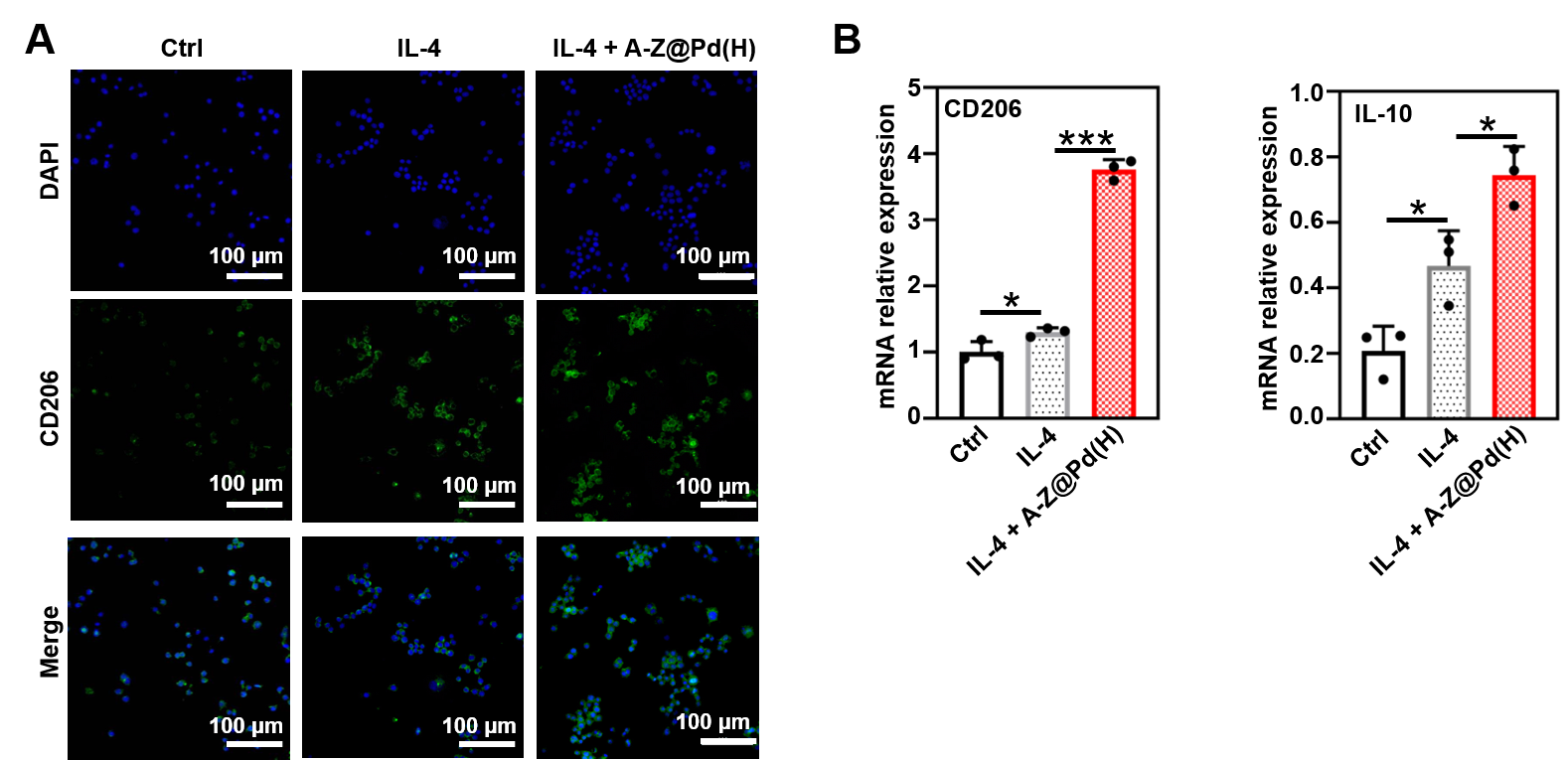

Supplement: Supplementary 1 — Figs. S1 to S30 Tables S1 and S2 [file research.0540.f1.zip › S22.tif]

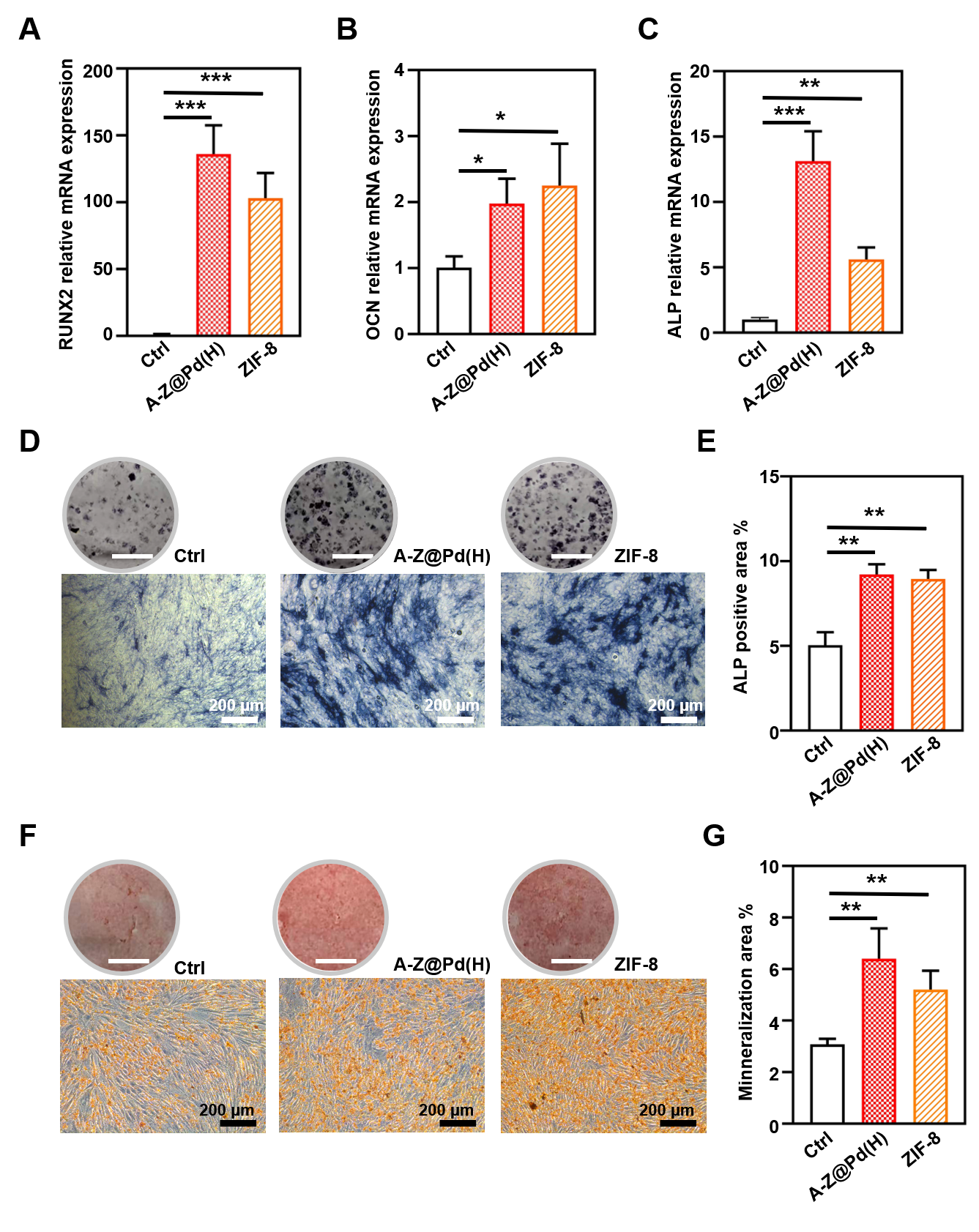

Supplement: Supplementary 1 — Figs. S1 to S30 Tables S1 and S2 [file research.0540.f1.zip › S23.tif]

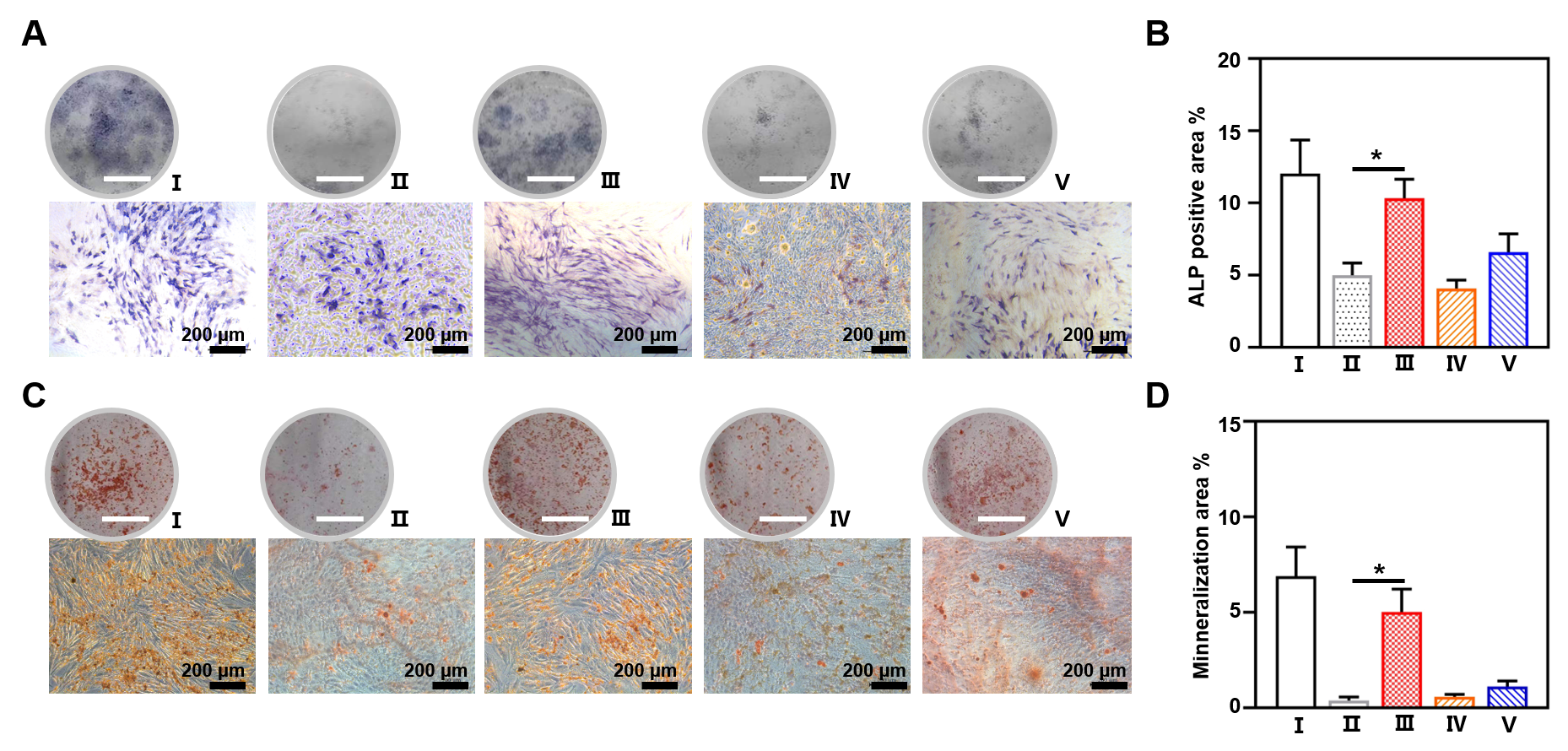

Supplement: Supplementary 1 — Figs. S1 to S30 Tables S1 and S2 [file research.0540.f1.zip › S24.tif]

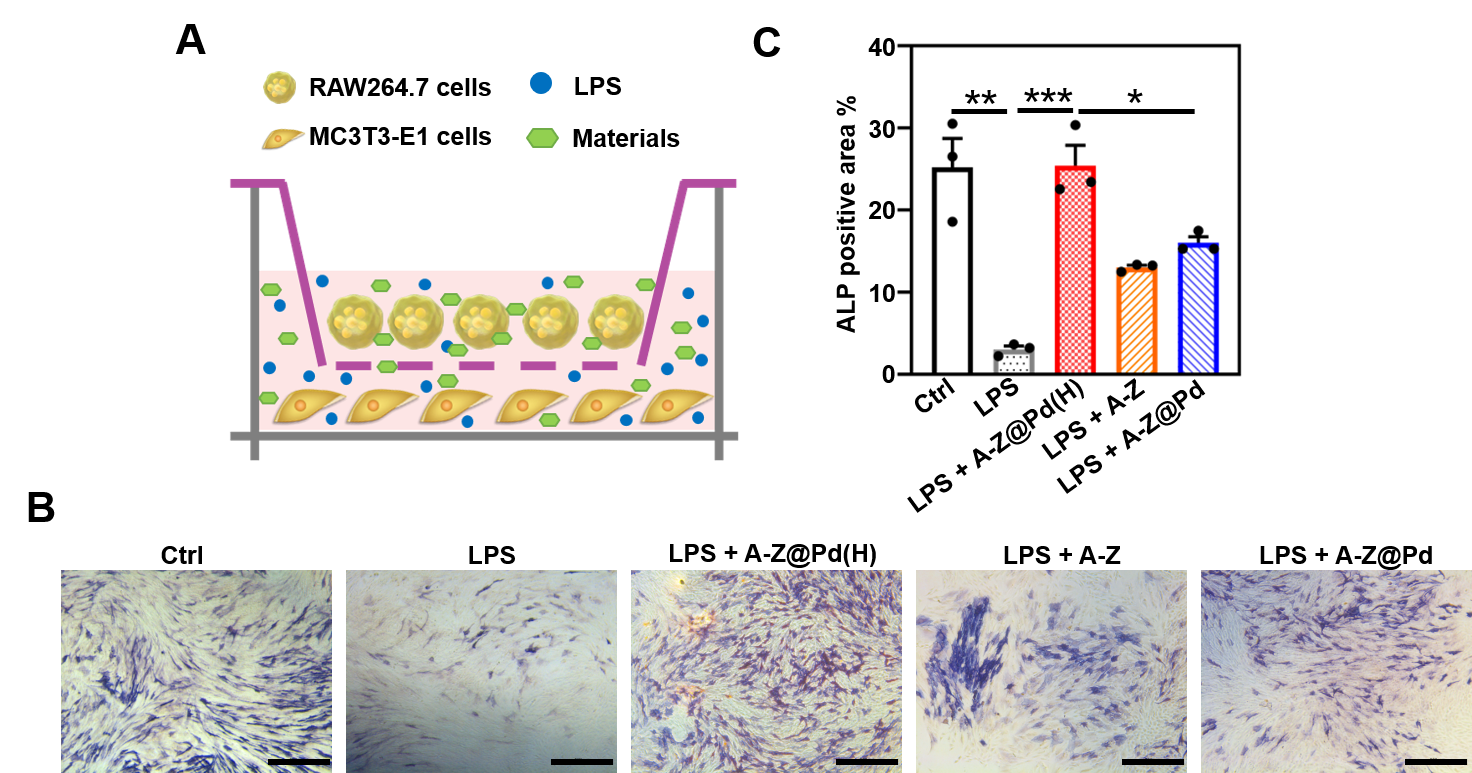

Supplement: Supplementary 1 — Figs. S1 to S30 Tables S1 and S2 [file research.0540.f1.zip › S25.tif]

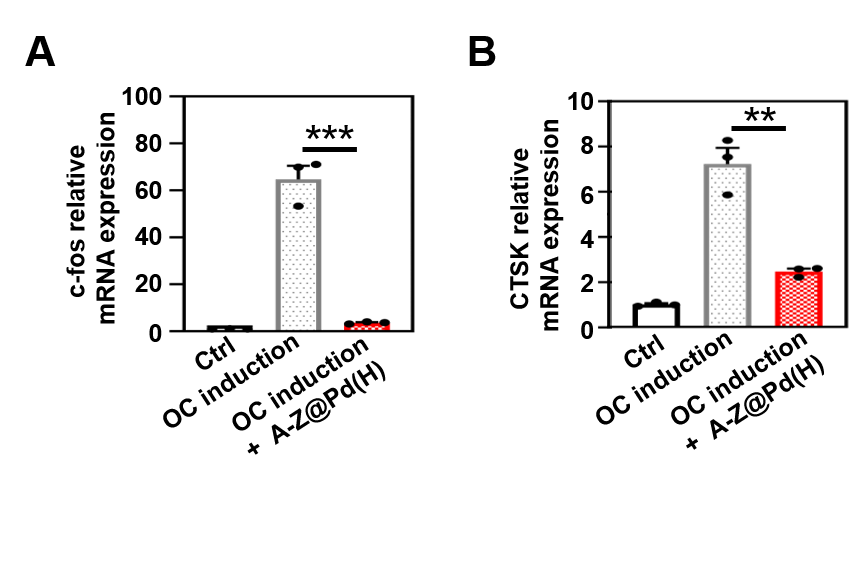

Supplement: Supplementary 1 — Figs. S1 to S30 Tables S1 and S2 [file research.0540.f1.zip › S26.tif]

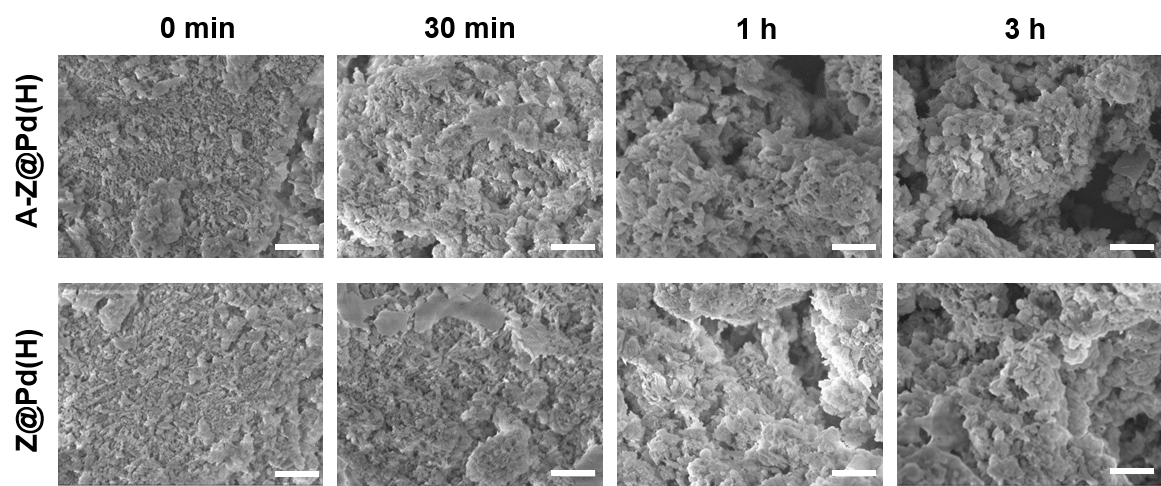

Supplement: Supplementary 1 — Figs. S1 to S30 Tables S1 and S2 [file research.0540.f1.zip › S27.tif]

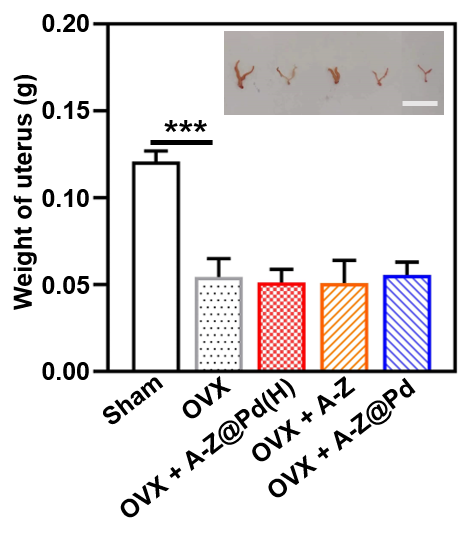

Supplement: Supplementary 1 — Figs. S1 to S30 Tables S1 and S2 [file research.0540.f1.zip › S28.tif]

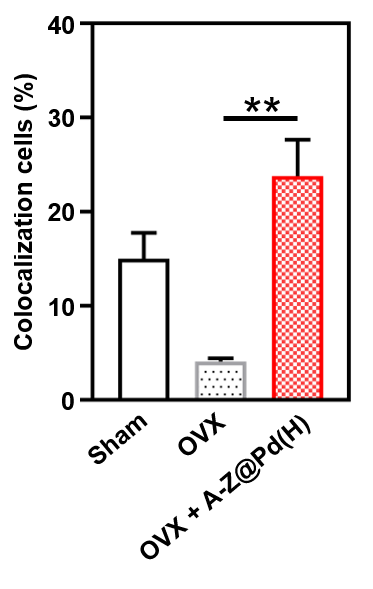

Supplement: Supplementary 1 — Figs. S1 to S30 Tables S1 and S2 [file research.0540.f1.zip › S29.tif]

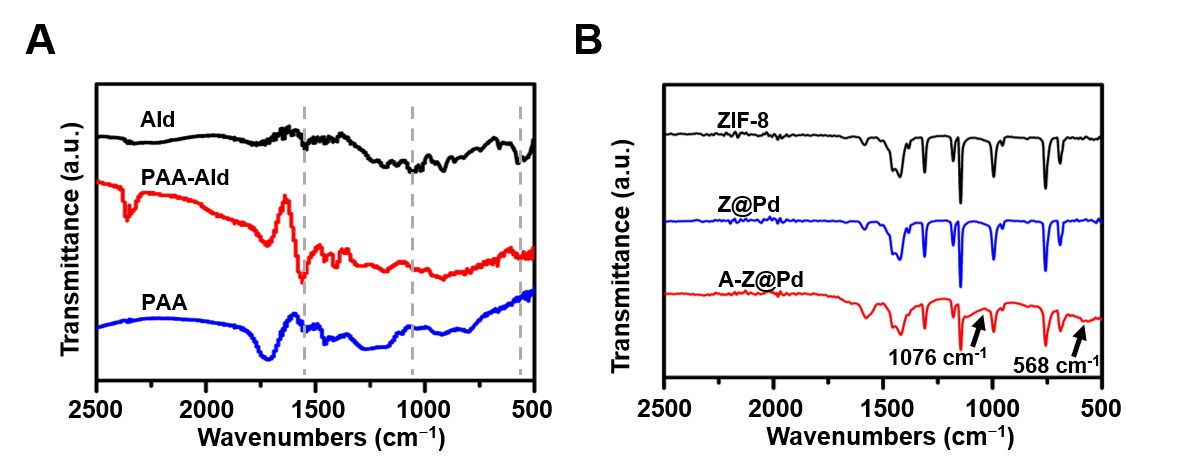

Supplement: Supplementary 1 — Figs. S1 to S30 Tables S1 and S2 [file research.0540.f1.zip › S3.tif]

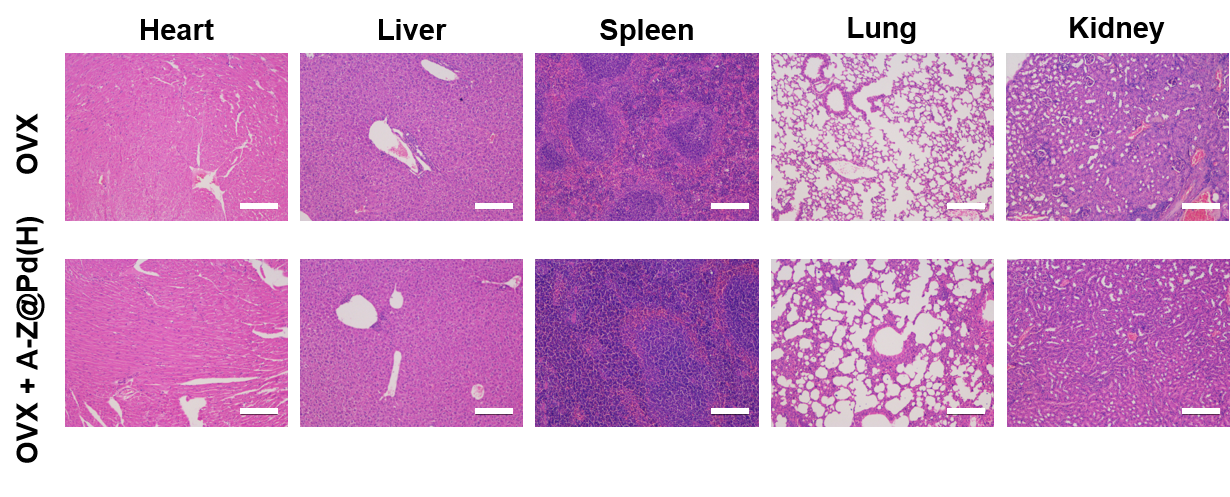

Supplement: Supplementary 1 — Figs. S1 to S30 Tables S1 and S2 [file research.0540.f1.zip › S30.tif]

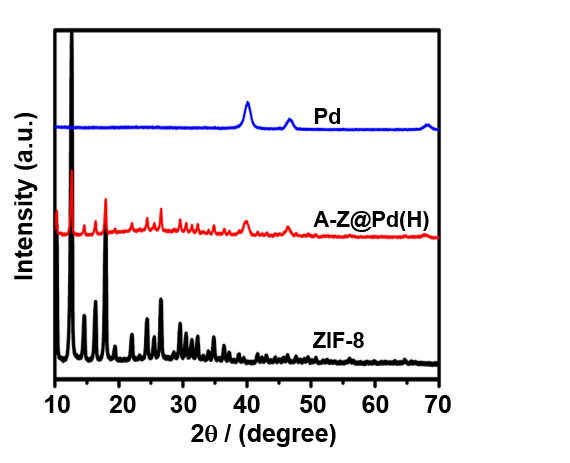

Supplement: Supplementary 1 — Figs. S1 to S30 Tables S1 and S2 [file research.0540.f1.zip › S4.tif]

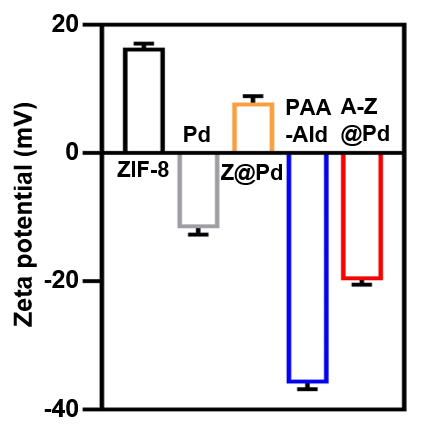

Supplement: Supplementary 1 — Figs. S1 to S30 Tables S1 and S2 [file research.0540.f1.zip › S5.tif]

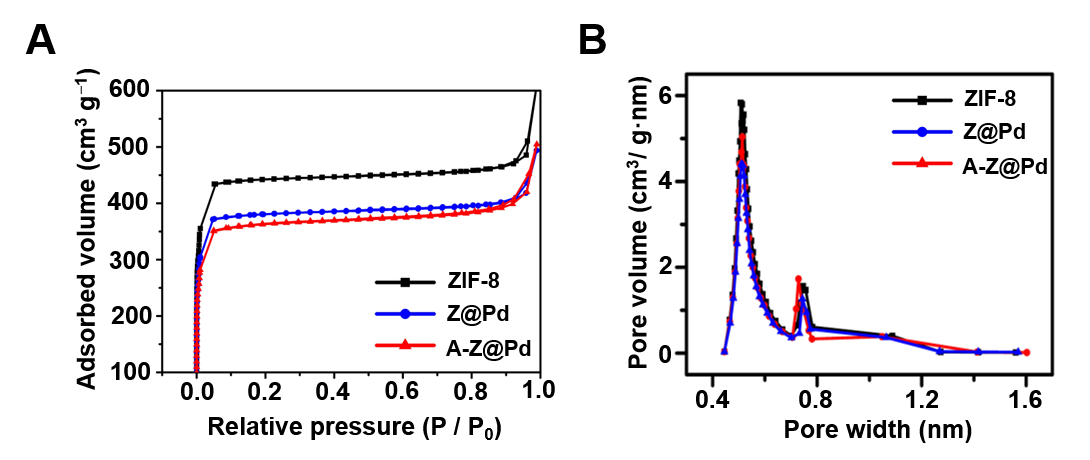

Supplement: Supplementary 1 — Figs. S1 to S30 Tables S1 and S2 [file research.0540.f1.zip › S6.tif]

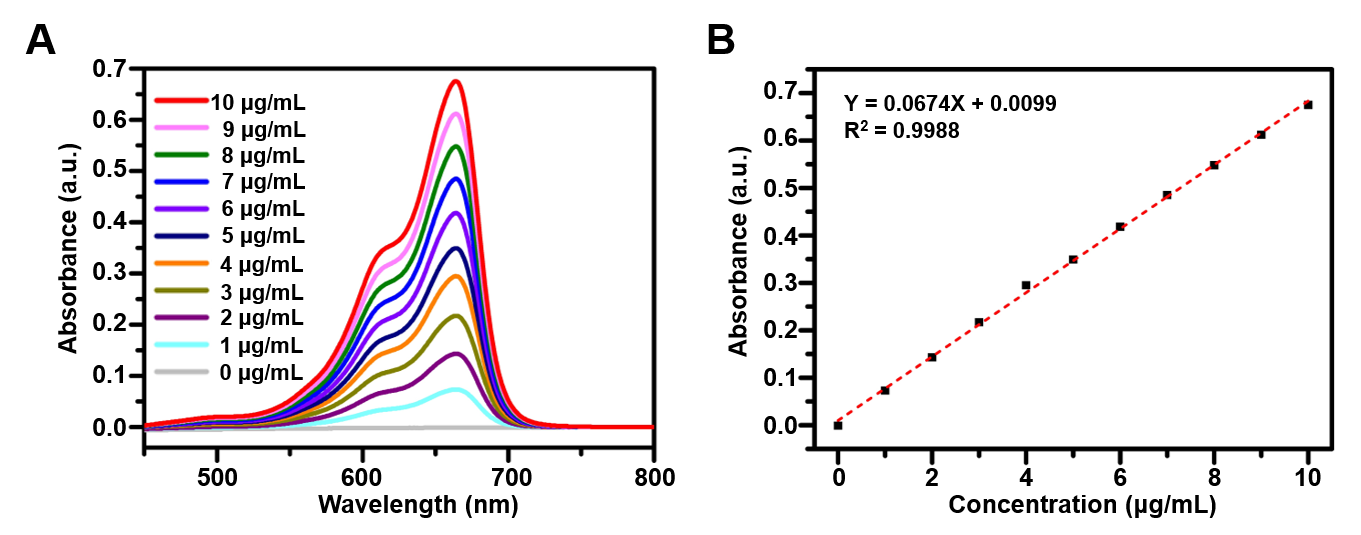

Supplement: Supplementary 1 — Figs. S1 to S30 Tables S1 and S2 [file research.0540.f1.zip › S7.tif]

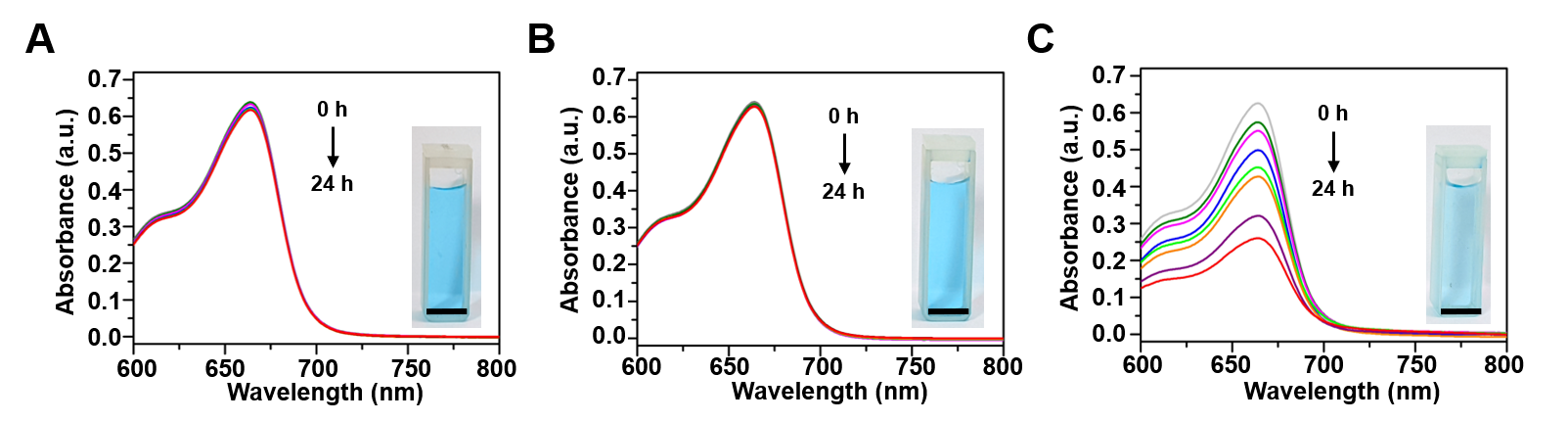

Supplement: Supplementary 1 — Figs. S1 to S30 Tables S1 and S2 [file research.0540.f1.zip › S8.tif]

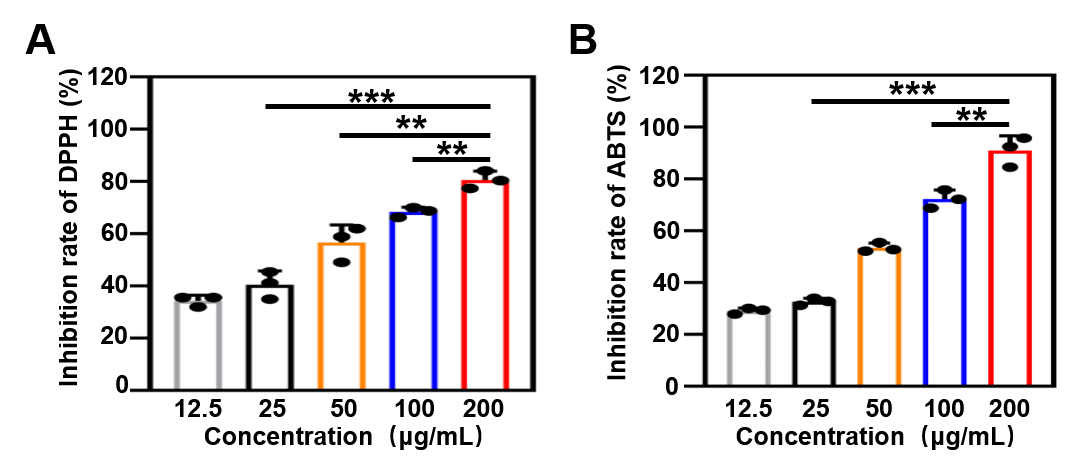

Supplement: Supplementary 1 — Figs. S1 to S30 Tables S1 and S2 [file research.0540.f1.zip › S9.tif]
